# Synthesis of Novel 7-Phenyl-2,3-Dihydropyrrolo[2,1-*b*]Quinazolin-9(1*H*)-ones as Cholinesterase Inhibitors Targeting Alzheimer’s Disease Through Suzuki–Miyaura Cross-Coupling Reaction

**DOI:** 10.3390/molecules30132791

**Published:** 2025-06-28

**Authors:** Davron Turgunov, Lifei Nie, Azizbek Nasrullaev, Zarifa Murtazaeva, Bianlin Wang, Dilafruz Kholmurodova, Rustamkhon Kuryazov, Jiangyu Zhao, Khurshed Bozorov, Haji Akber Aisa

**Affiliations:** 1 Department of Organic Synthesis and Bioorganic Chemistry, Institute of Biochemistry, Samarkand State University, University Blvd. 15, Samarkand 140104, Uzbekistan; 2 State Key Laboratory Basis of Xinjiang Indigenous Míicinal Plants Resource Utilization, Xinjiang Technical Institute of Physics and Chemistry, Chinese Academy of Sciences, South Beijing Rd 40-1, Urumqi 830011, China; 3 Scientific and Practical Center of Immunology, Allergology and Human Genomics, Samarkand State Medical University, Makhdum-i Aʿẓam st. 18, Samarkand 140104, Uzbekistan; 4 Department of Chemistry, Urgench State University, Kh. Olimjon st. 14, Urgench 220100, Uzbekistan

**Keywords:** Acetylcholinesterase (AChE), Alzheimer’s disease, Butyrylcholinesterase (BChE), carbon–carbon bond (C-C), deoxyvasicinone, mackinazolinone, Pd catalyst, quinazoline, Suzuki–Miyaura cross-coupling

## Abstract

An important field of research in medicinal and organic chemistry involves halogen-containing heterocyclic synthones, which form the backbone of more complex organic compounds. This study aimed to design and synthesize 28 novel derivatives of 7-aryl-2,3-dihydropyrrolo[2,1-*b*]quinazolin-9(1*H*)-one. The derivatives were created from 7-bromoquinoline intermediates to evaluate their potential as cholinesterase inhibitors for treating neurodegenerative diseases such as Alzheimer’s disease. The conditions for the Suzuki–Miyaura cross-coupling reaction were optimized to improve yield and purity. The derivatives were evaluated for their anticholinesterase activity using Ellman’s method, revealing that it most effectively inhibited cholinesterase within the micromolar range. 7-(3-Chloro-4-fluorophenyl)-2,3-dihydropyrrolo[2,1-*b*]quinazolin-9(1*H*)-one derivative exhibited the highest inhibitory potency, with an IC_50_ value of 6.084 ± 0.26 μM. Additionally, molecular dynamics simulations provided insight into how this lead compound interacts with the enzyme, suggesting its potential as a drug candidate for Alzheimer’s disease.

## 1. Introduction

The global pharmaceutical industry focuses on developing new drugs and assays for various diseases [1]. However, many conditions, particularly neurodegenerative diseases, lack effective treatments, resulting in millions of deaths each year [2,3,4]. The 2019 pandemic of the novel coronavirus (SARS-CoV-2) also created a significant public health and economic crisis [5,6].

**Figure 1:**
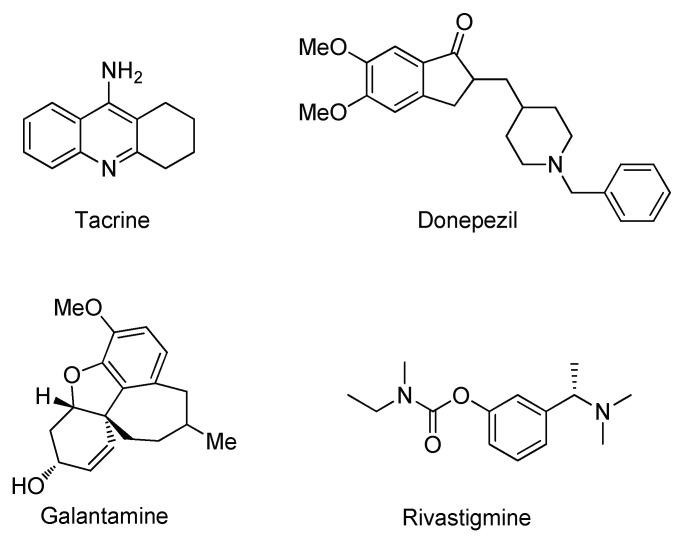
Structures of AChE inhibitors for managing AD.

Alzheimer’s disease (AD) is a condition that affects the brain and can cause serious health issues, especially in older adults [7,8]. The primary symptoms include memory loss, confusion, and cognitive difficulties [9,10]. Currently, more than 35.6 million people around the world have AD, and this number could rise to 115.4 million by 2050 [11,12,13]. Scientists have proposed several theories regarding the development of AD. These include the cholinergic hypothesis [14], the buildup of β-amyloid (Aβ) proteins [15], and the overactivation of tau proteins [16]. The cholinergic hypothesis has received significant focus. A variety of acetylcholinesterase inhibitors (AChEIs), including tacrine, donepezil, galantamine, and rivastigmine, have been used to treat cognitive impairment and memory loss in patients with mild to moderate AD [17,18] (Figure 1). However, these drugs also have notable side effects, including nausea, vomiting, decreased appetite, weight loss, and hepatotoxicity [19]. Therefore, it is essential to develop a new AChE inhibitor that offers greater AChE inhibition and reduced toxicity.

**Figure 2:**
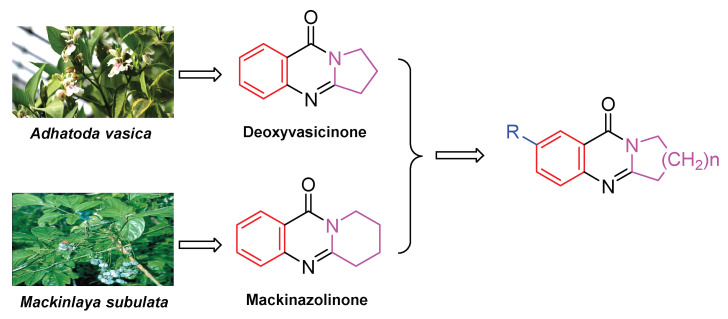
The structures of deoxyvasicinone and mackinazolinone alkaloids and the design of the present work.

Biologically active natural products are a crucial source of medicinal drugs [20,21,22,23]. Deoxyvasicinone and mackinazolinone (Figure 2) are the main active compounds found in *Peganum harmala* and *Mackinlaya* sp. [24,25]. They include a quinazolinone [26] moiety linked to a pyrrolidine. These compounds demonstrate antibacterial [27], anti-inflammatory [28], and antiproliferative effects [29]. Our research group has conducted several successful studies on the synthesis and biological properties of deoxyvasicinone and its A-ring-modified analogs [30,31,32,33,34,35,36]. Recently, tricyclic quinazolines have been recognized as promising cholinesterase inhibitors due to their structural resemblance to tacrine [37,38]. These tricyclic alkaloids exhibit moderate inhibitory effects on AChE and BChE. Additionally, a literature review revealed that deoxyvasicinone derivatives can inhibit AChEs [39,40,41,42].

Additionally, a modified version of deoxyvasicinone that was created using several five-membered heterocycles [43], such as thiophene, oxazole, thiazole [44], pyrazole [45], pyrrole [35,36], furan [35,36], and imidazole, resulted in a promising deoxyvasicinone-type tricyclic scaffold that exhibits anticancer potential. In our ongoing investigation into AChE and BuChE inhibitors, we have identified 7-aryl-2,3-dihydropyrrolo[2,1-*b*]quinazolin-9(1*H*)-ones as key candidates for further exploration. These compounds were synthesized from 6-bromoquinoline intermediates and underwent a Suzuki–Miyaura cross-coupling reaction [46,47,48,49] to facilitate the formation of new carbon–carbon (C-C) bonds within the target molecules (Figure 2).

## 2. Results and Discussion

### 2.1. Synthesis

**Scheme 1:**
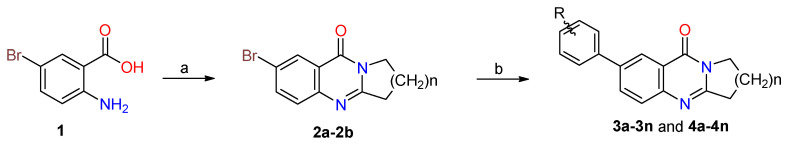
General synthetic route for the target compounds. Reagents and conditions: (a) lactams, POCl_3_, toluene, reflux 5 h; (b) boronic acids, Pd catalyst.

An important field of research in medicinal and organic chemistry involves halogen-containing heterocyclic synthones [50,51,52], which form the backbone of more complex organic compounds. These synthones use other sites for attachment to specific types of rapidly forming molecules. We investigated the formation of 7-aryl-2,3-dihydropyrrolo[2,1-*b*]quinazolin-9(1*H*)-ones through Suzuki cross-coupling, thereby creating new C-C bonds. We synthesized compounds **2a**–**2b** by reacting 2-amino-5-bromobenzoic acid (**1**) with lactams in the presence of phosphorus oxychloride. Then, we performed a Suzuki coupling reaction with various substituted phenylboronic acids to yield the target compounds **3a**–**3n** and **4a**–**4n** (Scheme 1).

The optimization of reaction conditions for the Suzuki coupling reaction is presented in detail in Table 1.

6-Bromquinazolinone (**2b**) underwent cross-coupling with o-trifluoromethyl boronic acid in the presence of different Pd catalysts and a basic medium condition. Initially, a comparative study was conducted to evaluate the efficacy of four distinct palladium catalysts in the presence of K_2_CO_3_ as the base, using a toluene solvent system. The results indicated that Pd(PPh_3_)_4_ yielded the highest synthesis efficiency, achieving an impressive target compound yield of 56%. Following that, we evaluated various solvents, ultimately identifying toluene as the most effective. Different bases were tested as substitutes for K_2_CO_3_, with Cs_2_CO_3_ yielding the best results for forming the desired compound **4n**. In addition, the optimization of the toluene/water ratio was explored, revealing that a 3:1 ratio yielded the most significant result, reaching an optimal product yield of 96%.

**Scheme 2:**
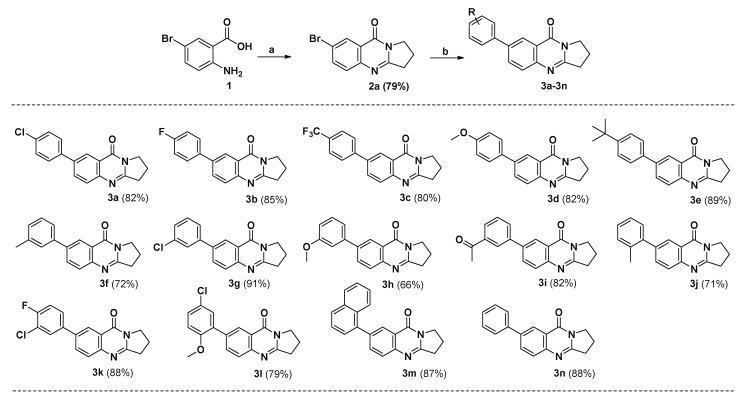
Synthetic route for the target compounds **3a**-**3n**. Reagents and conditions: (a) 2-Pyrrolidone, POCl_3_, toluene, reflux 5 h; (b) boronic acids, Cs_2_CO_3_, Pd(PPh_3_)_4_, PhCH_3_/H_2_O = 3:1, 110 °C, reflux 8 h.

**Scheme 3:**
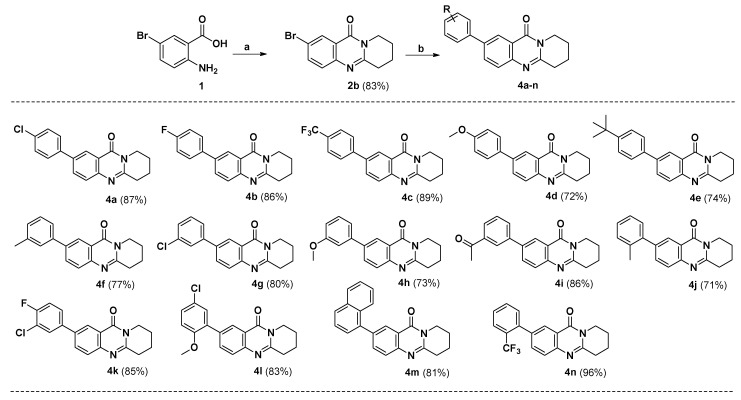
Synthetic route for the target compounds **4a**–**4n**. Reagents and conditions: (a) 2-Piperidinone, POCl_3_, toluene, reflux 5 h; (b) boronic acids, Cs_2_CO_3_, Pd(PPh_3_)_4_, PhCH_3_/H_2_O = 3:1, 110 °C, reflux 8 h.

Under these refined reaction conditions, compounds **3a**–**3n** and **4a**–**4n** were successfully synthesized (Scheme 2 and Scheme 3), with their structures thoroughly characterized using ^1^H NMR, ^13^C NMR, and high-resolution mass spectrometry (HRMS).

**Figure 3:**
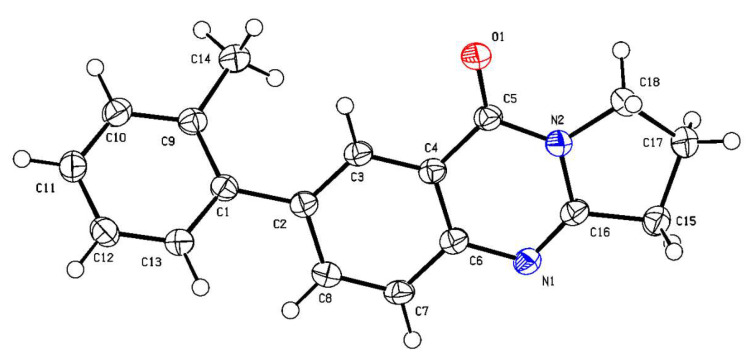
Crystal structure of compound **3j** (CCDC: 2392852).

A single crystal of **3j** (Figure 3) was obtained in ethanol, consistent with the structural formula in Scheme 1.

### 2.2. Inhibitory Activities of AChE and BChE for **3a**–**3n** and **4a**–**4n**

We assessed the inhibitory activities of compounds **3a**–**3n** and **4a**–**4n** against AChE and BChE using the Ellman method [53,54,55], with huperzine A [56,57] and Tacrine [58,59] serving as the positive controls. The results are presented as IC_50_ values.

As shown in Table 2, most compounds significantly inhibited AChE and BChE within the micromolar range. Notably, substituent groups on the benzene ring had a considerable effect on potency. Compounds with methyl, methoxy, or halogen substituents exhibited potent inhibition of both enzymes; compound **3k** was the most effective among the tri-methylene series. Compound **3k** exhibited IC_50_ values of 6.084 ± 0.26 and 29.01 ± 0.74 for AChE and BChE, respectively. The position of the substituent on the benzene ring also played a crucial role: meta-substituted compounds exhibited the highest activity, followed by para- and ortho-substituted ones. For example, among the tetra-methylene series, derivative **4f** showed the highest activity toward AChE and BChE, with IC_50_ values of 6.39 ± 0.31 and 10.74 ± 0.64, respectively. Although compound **4f** showed notable activity for both cholinesterases, it had poor solubility in common organic solvents, which significantly limited its handling and further analysis. Therefore, compound **3k** appears to be a more representative candidate for further discussion because of its favorable balance of activity and solubility. Other synthesized quinazolinones showed moderate or no inhibition.

**Figure 4:**
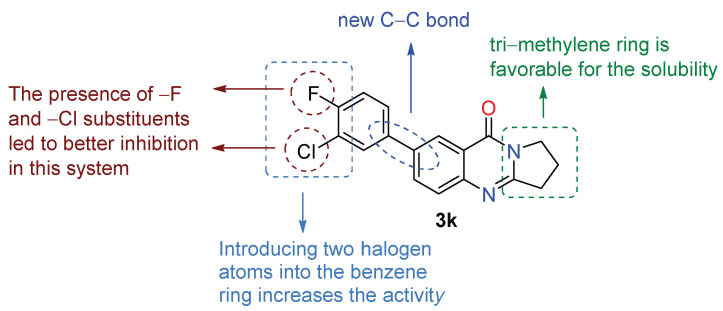
The analysis of the SAR by compound **3k**.

It should be noted that introducing the two halogen atoms into the aromatic benzene section led to favorable inhibition (Figure 4). In addition, when comparing two side-chain cycloalkane rings (methylene group), the tri-methylene ring provided better solubility than the tetramethylene ring in organic solvents.

### 2.3. Docking Study

**Figure 5:**
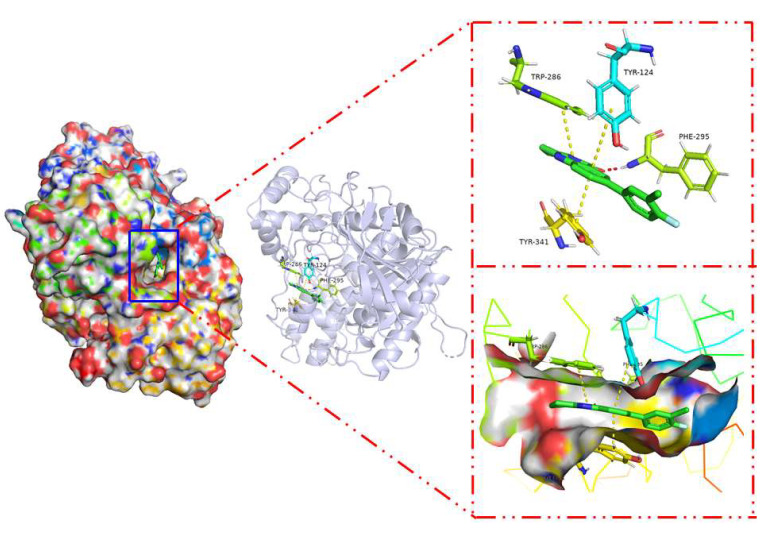
Molecule docking results: 3D docking models of compound **3k** with AChE (PDB code: 4ey7).

To investigate the binding mode of compound **3k** to the protein (PDB: 4ey7), we conducted docking simulations using AutoDock [60]. As illustrated in Figure 5, the molecular docking results indicate that hydrogen bonding and π-π stacking were the two primary interactions facilitating the binding of this ligand to the receptor. Compound **3k** could be effectively accommodated within the protein pocket, with the keto carbonyl group on the pyrimidine ring forming a hydrogen bond with PHE-295. Additionally, the benzo-pyrimidine ring of the ligand interacted with TYR-341, TYR-124, and TRP-286 through π-π stacking interactions. These interactions enhanced the inhibitory activity of the protein, providing a plausible explanation for its significant inhibitory effect against AChE.

### 2.4. Molecular Dynamics

Molecular dynamics (MD) simulations can predict the binding state of a ligand to a receptor within a biological environment [61,62]. In this study, compound **3k** and the protein (PDB: 4ey7) were chosen as the initial structures for the MD simulations. A molecular dynamics calculation lasting 100 nanoseconds was conducted using GROMACS 2024 software [63].

#### 2.4.1. Root Mean Square Deviation (RMSD)

**Figure 6:**
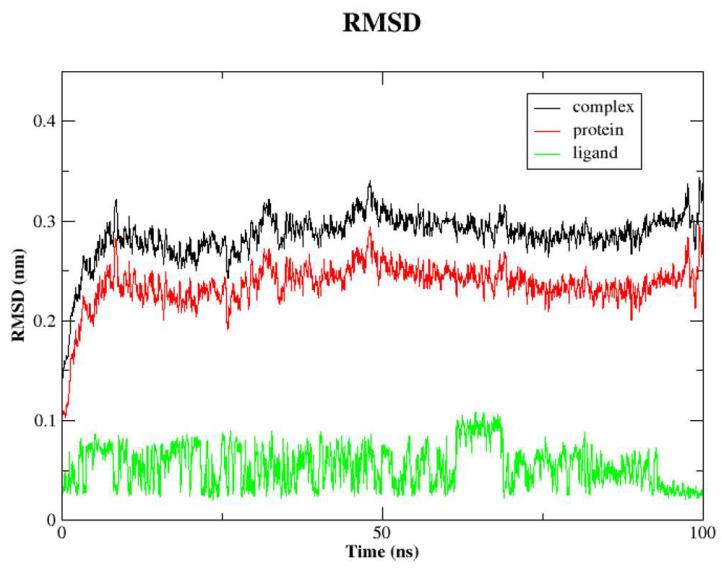
RMSD plot of **3k**–4ey7 complex (black), 4ey7 (red), and **3k** (green) across 100 ns MD simulation trajectory.

Root mean square difference (RMSD) evaluates the overall conformational changes of the protein, ligand, and protein–ligand complex relative to the initial structure of the system [64,65]. As shown in Figure 6, the RMSD values for the proteins and protein–ligand complexes exhibit fluctuations during the initial 0–15 ns period, eventually stabilizing at approximately 0.3 nm and 0.25 nm, respectively. In contrast, the RMSD of ligand **3k** exhibited fluctuations at the binding site from 0 to 10 ns, followed by a period of equilibration from 10 to 100 ns, ultimately stabilizing at approximately 0.075 nm. The minimal fluctuations and low RMSD values indicate that the protein backbone’s conformation remains stable compared to its initial structure.

#### 2.4.2. Root Mean Square Fluctuation (RMSF)

**Figure 7:**
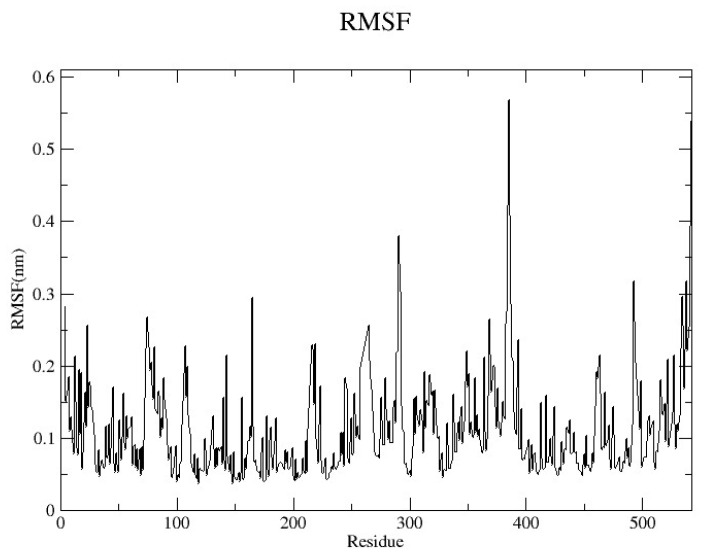
RMSF plot of protein 4ey7 across 100 ns MD simulation trajectory.

RMSF analyzes the fluctuations of each amino acid in a protein relative to the initial structure [66]. Lower values are consistently observed in the residues surrounding the site. As shown in Figure 7, the structural fluctuations of the amino acid residues in the protein are less than 0.3 nm, except for a few residues, indicating that the protein has undergone minimal change.

#### 2.4.3. Radius of Gyration (Rg)

**Figure 8:**
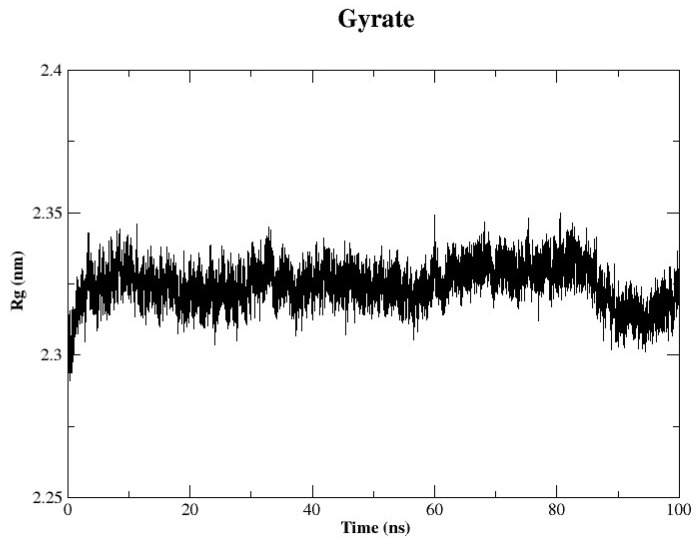
Radius of gyration plot of **3k**–**4ey7** complex across 100 ns MD simulation trajectory.

The radius of gyration (Rg) is utilized to assess the compactness of a system’s structure; a lower Rg value indicates a more folded configuration [67]. As illustrated in Figure 8, the **3k**-4EY7 complex exhibits a distinct conformational change between 0–10 ns and 85–100 ns, gradually decreasing and stabilizing at approximately 2.33 nm.

#### 2.4.4. Hydrogen Bonds Analysis

**Figure 9:**
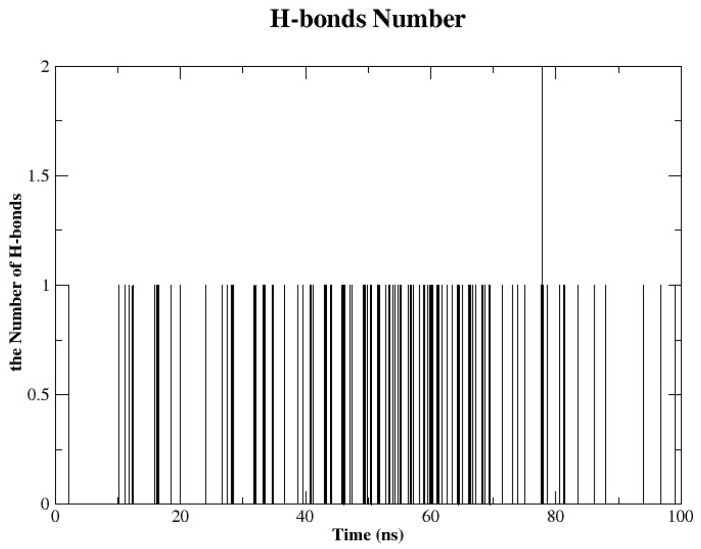
Number of H-bonds in **3k**–**4ey7** complex across 100 ns MD simulation trajectory.

Hydrogen bonding is the most significant interaction that stabilizes protein–ligand complex systems [9,68,69]. Therefore, molecular dynamics (MD) simulations were performed to analyze the information regarding the hydrogen bonds formed between the protein and ligands. The results indicated that hydrogen bonding interactions could occur between ligand **3k** and protein 4ey7 (Figure 9), with a hydrogen bond count of 1. This suggests that the ligand and protein could establish specific interactions, thereby maintaining a stable conformation.

## 3. Materials and Methods

### 3.1. Materials

All reagents were of analytical grade and were used directly without further purification. All reactions were monitored by analytical thin-layer chromatography. Visualization was performed using 254 nm UV light, and column chromatography was conducted with 100–200 mesh silica gel. Melting points were determined using a Buchi B-540 melting point apparatus. Hydrogen and carbon spectra were obtained using 400 MHz NMR spectroscopy. High-resolution mass spectra were acquired using an AB SCIEX QSTAR Elite quadrupole time-of-flight mass spectrometer.

### 3.2. Experimental Procedures

#### 3.2.1. General Procedure of Preparation of **2a**–**2b**

A solution of compound **1** (1 mmol) and lactam (1.2 mmol) was cooled to 0–5 °C, and then POCl_3_ (2 mL) was added dropwise. Toluene (10 mL) was added to the mixture and stirred at reflux for 5 h. The solvent and excess POCl_3_ were evaporated under reduced pressure. Next, a 10% NH_4_OH solution was added until the pH reached 9, after which the mixture was extracted with DCM (2 × 30 mL). The organic phase was washed with a brine solution. The organic layer was then separated and dried over anhydrous MgSO_4_. It was then filtered and concentrated under reduced pressure to yield the crude product. This product was then purified by silica gel chromatography to produce the pure corresponding compounds **2a** and **2b**.

7-Bromo-2,3-dihydropyrrolo[2,1-*b*]quinazolin-9(1*H*)-one (2a). Yield 79%, yellow solid, m.p: 163–164 °C. ^1^H NMR (400 MHz, CDCl_3_) *δ* 8.32 (d, *J* = 2.3 Hz, 1H), 7.73 (dd, *J* = 8.8, 2.4 Hz, 1H), 7.45 (d, *J* = 8.7 Hz, 1H), 4.15 (t, *J* = 7.4 Hz, 2H), 3.12 (t, *J* = 7.9 Hz, 2H), 2.67–2.00 (m, 2H). ^13^C NMR (100 MHz, CDCl_3_) *δ* 159.90, 159.67, 147.91, 137.23, 128.85, 128.60, 121.87, 119.59, 46.62, 32.50, 19.44. HRMS (ESI) calcd for C_11_H_9_BrN_2_O [M+H]^+^ 264.9971, found 264.9987.

2-Bromo-6,7,8,9-tetrahydro-11*H*-pyrido[2,1-*b*]quinazolin-11-one (2b). Yield 83%, yellow solid, m.p: 120–121 °C. ^1^H NMR (400 MHz, CDCl_3_) *δ* 8.34 (d, *J* = 2.3 Hz, 1H), 7.75 (dd, *J* = 8.7, 2.3 Hz, 1H), 7.44 (d, *J* = 8.7 Hz, 1H), 4.04 (t, *J* = 6.2 Hz, 2H), 2.96 (t, *J* = 6.6 Hz, 2H), 2.17–1.85 (m, 4H). ^13^C NMR (100 MHz, CDCl_3_) *δ* 160.93, 155.39, 146.08, 137.33, 129.08, 128.24, 121.69, 119.36, 42.57, 31.90, 21.99, 19.19. HRMS (ESI) calcd for C_12_H_11_BrN_2_O [M+H]^+^ 279.0128, found 279.0135.

#### 3.2.2. General Procedure for the Synthesis of Compounds **3a**–**3n** and **4a**–**4n**

A mixture of compound **2a**–**2b** (1 mmol), arylboronic acid (1.2 mmol), cesium carbonate (1.5 mmol), and tetrakis(triphenylphosphine)palladium (0.05 mmol) in toluene/water (3/1) was heated at 110 °C under reflux for 8 h under an inert atmosphere. The reaction product was monitored by TLC and extracted with aqueous NH_4_Cl solution (15 mL) and ethyl acetate (15 × 3 mL). It was then washed with an aqueous solution of NaHCO_3_ and brine. The organic phase was then separated, filtered, and dried over Na_2_SO_4_ to give compounds **3a**–**3n** and **4a**–**4n**. The product was purified by silica gel column chromatography (ethyl acetate/petroleum ether = 3:1).

7-(4-Chlorophenyl)-2,3-dihydropyrrolo[2,1-*b*]quinazolin-9(1*H*)-one (3a). Yield 82%, gray solid, m.p: 180–181 °C. ^1^H NMR (400 MHz, CDCl_3_) δ8.45 (d, *J* = 2.2 Hz, 1H), 7.92 (dd, *J* = 8.5, 2.3 Hz, 1H), 7.71 (d, *J* = 8.5 Hz, 1H), 7.61 (d, *J* = 8.5 Hz, 2H), 7.43 (d, *J* = 8.6 Hz, 2H), 4.29–4.18 (m, 2H), 3.21 (t, *J* = 8.0 Hz, 2H), 2.39–2.20 (m, 2H). ^13^C NMR (100 MHz, CDCl_3_) δ 160.85, 159.67, 148.27, 138.03, 137.87, 133.93, 132.81, 129.10, 128.36, 127.32, 124.20, 120.74, 46.63, 32.52, 19.53. HRMS (ESI) calcd for C_17_H_13_ClN_2_O [M+H]^+^ 297.0789, found 297.0799.

7-(4-Fluorophenyl)-2,3-dihydropyrrolo[2,1-*b*]quinazolin-9(1*H*)-one (3b). Yield 85%, yellow solid, m.p: 156–157 °C; ^1^H NMR (400 MHz, CDCl_3_) δ 8.43 (d, *J* = 2.2 Hz, 1H), 7.91 (dd, *J* = 8.5, 2.2 Hz, 1H), 7.70 (d, *J* = 8.5 Hz, 1H), 7.64 (dd, *J* = 8.8, 5.2 Hz, 2H), 7.15 (t, *J* = 8.8 Hz, 2H), 4.26–4.19 (m, 2H), 3.20 (t, *J* = 8.0 Hz, 2H), 2.35–2.25 (m, 2H). ^13^C NMR (100 MHz, CDCl_3_) δ 163.49, 160.75 (d, J = 55.7 Hz), 159.06, 147.66, 137.71, 135.31 (d, J = 3.1 Hz), 132.45, 128.32 (d, J = 8.4 Hz), 126.83, 123.67, 120.27, 115.41 (d, J = 21.5 Hz), 46.17, 32.07, 19.10. HRMS (ESI) calcd for C_17_H_13_FN_2_O [M+H]^+^ 281.1085, found 281.1092.

7-(4-(Trifluoromethyl)phenyl)-2,3-dihydropyrrolo[2,1-*b*]quinazolin-9(1*H*)-one (3c). Yield 80%, white solid, m.p: 191–192 °C. ^1^H NMR (400 MHz, CDCl_3_) δ 8.51 (d, *J* = 2.2 Hz, 1H), 7.97 (dd, *J* = 8.5, 2.3 Hz, 1H), 7.83–7.62 (m, 5H), 4.28–4.20 (m, 2H), 3.22 (t, *J* = 7.9 Hz, 2H), 2.38–2.26 (m, 2H). ^13^C NMR (100 MHz, CDCl_3_) δ 160.81, 159.99, 148.75, 143.09, 137.55, 132.97, 131.96 (d, J = 12.0 Hz), 129.78 (d, J = 32.6 Hz), 128.46 (d, J = 12.0 Hz), 127.46 (d, J = 9.8 Hz), 125.89 (q, J = 3.8 Hz), 124.75, 120.83, 46.65, 32.56, 19.51. HRMS (ESI) calcd for C_18_H_13_F_3_N_2_O [M+H]^+^ 331.1053, found 331.1063.

7-(4-Methoxyphenyl)-2,3-dihydropyrrolo[2,1-*b*]quinazolin-9(1*H*)-one (3d). Yield 82%, white solid, m.p: 173–174 °C. ^1^H NMR (400 MHz, CDCl_3_) δ 8.43 (d, J = 2.2 Hz, 1H), 7.93 (dd, J = 8.5, 2.2 Hz, 1H), 7.68 (d, J = 8.5 Hz, 1H), 7.62 (d, J = 9.0 Hz, 2H), 6.99 (d, J = 9.0 Hz, 2H), 4.24–4.20 (m, 2H), 3.85 (s, 3H), 3.19 (t, J = 7.9 Hz, 2H), 2.36–2.22 (m, 2H). ^13^C NMR (100 MHz, CDCl_3_) δ 161.20, 159.73, 159.34, 147.77, 139.05, 132.94, 132.28, 128.41, 127.25, 123.74, 120.87, 114.59, 55.57, 46.80, 32.68, 19.77. HRMS (ESI) calcd for C_18_H_16_N_2_O_2_ [M+H]^+^ 293.1285, found 293.1289.

7-(4-(*tert*-Butyl)phenyl)-2,3-dihydropyrrolo[2,1-*b*]quinazolin-9(1*H*)-one (3e). Yield 89%, white solid, m.p: 221–222 °C. ^1^H NMR (400 MHz, CDCl_3_) δ 8.50 (d, *J* = 2.2 Hz, 1H), 7.98 (dd, *J* = 8.5, 2.2 Hz, 1H), 7.70 (d, *J* = 8.5 Hz, 1H), 7.64 (d, *J* = 8.3 Hz, 2H), 7.49 (d, *J* = 8.2 Hz, 2H), 4.26–4.18 (m, 2H), 3.19 (t, *J* = 7.9 Hz, 2H), 2.34–2.24 (m, 2H), 1.36 (s, 9H). ^13^C NMR (100 MHz, CDCl_3_) δ 161.00, 159.22, 150.88, 147.92, 139.00, 136.62, 132.95, 127.09, 126.75, 125.94, 123.97, 120.68, 46.56, 34.57, 32.48, 31.29, 19.56. HRMS (ESI) calcd for C_21_H_22_N_2_O [M+H]^+^ 319.1805, found 319.1813.

7-(m-Tolyl)-2,3-dihydropyrrolo[2,1-*b*]quinazolin-9(1*H*)-one (3f). Yield 72%, yellow solid, m.p: 156–157 °C. ^1^H NMR (400 MHz, CDCl_3_) δ 8.49 (d, *J* = 2.2 Hz, 1H), 7.97 (dd, *J* = 8.4, 2.2 Hz, 1H), 7.70 (d, *J* = 8.5 Hz, 1H), 7.53–7.46 (m, 2H), 7.35 (t, *J* = 7.6 Hz, 1H), 7.19 (d, *J* = 8.9 Hz, 1H), 4.27–4.20 (m, 2H), 3.20 (t, *J* = 7.9 Hz, 2H), 2.43 (s, 3H), 2.35–2.26 (m, 2H). ^13^C NMR (100 MHz, CDCl_3_) δ 161.02, 159.33, 148.10, 139.53, 139.28, 138.58, 133.11, 128.83, 128.50, 127.94, 127.12, 124.21, 120.66, 46.58, 32.51, 21.52, 19.56. HRMS (ESI) calcd for C_18_H_16_N_2_O [M+H]^+^ 277.1335, found 277.1345.

7-(3-Chlorophenyl)-2,3-dihydropyrrolo[2,1-*b*]quinazolin-9(1*H*)-one (3g). Yield 91%, white solid, m.p: 171–172 °C. ^1^H NMR (400 MHz, CDCl_3_) δ 8.46 (d, *J* = 2.2 Hz, 1H), 7.93 (dd, *J* = 8.5, 2.3 Hz, 1H), 7.71 (d, *J* = 8.7 Hz, 1H), 7.67 (t, *J* = 1.8 Hz, 1H), 7.56 (dt, *J* = 7.6, 1.3 Hz, 1H), 7.42–7.32 (m, 2H), 4.27–4.20 (m, 2H), 3.20 (t, *J* = 8.0 Hz, 2H), 2.36–2.26 (m, 2H). ^13^C NMR (100 MHz, CDCl_3_) δ 160.85, 159.77, 148.57, 141.42, 137.64, 134.88, 132.89, 130.16, 127.73, 127.41, 127.28, 125.29, 124.45, 120.77, 46.62, 32.55, 19.53. HRMS (ESI) calcd for C_17_H_13_ClN_2_O [M+H]^+^ 297.0789, found 297.0800.

7-(3-Methoxyphenyl)-2,3-dihydropyrrolo[2,1-*b*]quinazolin-9(1*H*)-one (3h). Yield 66%, yellow solid, m.p: 140–141 °C. ^1^H NMR (400 MHz, CDCl_3_) δ 8.49 (d, *J* = 2.2 Hz, 1H), 7.96 (dd, *J* = 8.5, 2.3 Hz, 1H), 7.70 (d, *J* = 8.5 Hz, 1H), 7.38 (t, *J* = 8.0 Hz, 1H), 7.27 (d, *J* = 6.5 Hz, 1H), 7.23–7.18 (m, 1H), 6.92 (dd, *J* = 8.2, 1.6 Hz, 1H), 4.26–4.20 (m, 2H), 3.88 (s, 3H), 3.20 (t, *J* = 7.9 Hz, 2H), 2.35–2.24 (m, 2H). ^13^C NMR (100 MHz, CDCl_3_) δ 161.00, 160.09, 159.47, 148.30, 141.11, 139.03, 133.16, 129.96, 127.19, 124.33, 120.67, 119.65, 113.38, 112.68, 55.38, 46.59, 32.52, 19.54. HRMS (ESI) calcd for C_18_H_16_N_2_O_2_ [M+H]^+^ 293.1285, found 293.1293.

7-(3-Acetylphenyl)-2,3-dihydropyrrolo[2,1-*b*]quinazolin-9(1*H*)-one (3i). Yield 82%, yellow solid, m.p: 228–229 °C. ^1^H NMR (400 MHz, CDCl_3_) δ8.51 (d, *J* = 2.3 Hz, 1H), 8.25 (t, *J* = 1.7 Hz, 1H), 7.98 (dd, *J* = 17.1, 8.1 Hz, 2H), 7.90–7.86 (m, 1H), 7.74 (d, *J* = 8.4 Hz, 1H), 7.57 (t, *J* = 7.7 Hz, 1H), 4.46–4.06 (m, 2H), 3.21 (t, *J* = 7.9 Hz, 2H), 2.67 (s, 3H), 2.39–2.21 (m, 2H). ^13^C NMR (100 MHz, CDCl_3_) δ197.89, 160.87, 159.79, 148.50, 140.17, 138.09, 137.77, 133.05, 131.71, 129.25, 127.61, 127.43, 126.89, 124.50, 120.78, 46.64, 32.54, 26.80, 19.53. HRMS (ESI) calcd for C_19_H_16_N_2_O_2_ [M+H]^+^ 305.1285, found 305.1295.

7-(o-Tolyl)-2,3-dihydropyrrolo[2,1-*b*]quinazolin-9(1*H*)-one (3j). Yield 71%. white solid, m.p: 176–177 °C. ^1^H NMR (400 MHz, CDCl_3_) δ8.24 (d, *J* = 1.0 Hz, 1H), 7.69 (d, *J* = 1.6 Hz, 2H), 7.52–7.08 (m, 4H), 4.41–4.01 (m, 2H), 3.21 (t, *J* = 8.0 Hz, 2H), 2.54–2.04 (m, 2H), 2.28 (s, 3H). ^13^C NMR (100 MHz, CDCl_3_) δ 160.91, 159.43, 147.67, 140.46, 140.19, 135.45, 135.31, 130.46, 129.86, 127.71, 126.54, 126.35, 125.93, 120.20, 46.57, 32.49, 20.45, 19.56. HRMS (ESI) calcd for C_18_H_16_N_2_O [M+H]^+^ 277.1335, found 277.1345.

7-(3-Chloro-4-fluorophenyl)-2,3-dihydropyrrolo[2,1-*b*]quinazolin-9(1*H*)-one (3k). Yield 88%, white solid, m.p: 182–183 °C. ^1^H NMR (400 MHz, CDCl_3_) δ 8.41 (d, *J* = 2.2 Hz, 1H), 7.88 (dd, *J* = 8.5, 2.2 Hz, 1H), 7.73–7.68 (m, 2H), 7.52 (ddd, *J* = 8.6, 4.5, 2.3 Hz, 1H), 7.22 (t, *J* = 8.7 Hz, 1H), 4.27–4.19 (m, 2H), 3.21 (t, *J* = 7.9 Hz, 2H), 2.37–2.24 (m, 2H). ^13^C NMR (100 MHz, CDCl_3_) δ 160.78, 159.14, 158.25 (d, J = 321.2 Hz), 148.43, 136.85, 132.71, 129.28, 127.46, 126.79 (d, J = 7.3 Hz), 124.29, 121.55 (d, J = 17.9 Hz), 120.78, 117.01 (d, J = 21.1 Hz), 46.65, 32.53, 19.52. HRMS (ESI) calcd for C_17_H_12_ClFN_2_O [M+H]^+^ 315.0695, found 315.0703.

7-(5-Chloro-2-methoxyphenyl)-2,3-dihydropyrrolo[2,1-*b*]quinazolin-9(1*H*)-one (3l). Yield 79%, white solid, m.p: 123–124 °C. ^1^H NMR (400 MHz, CDCl_3_) δ 8.39 (d, *J* = 2.2 Hz, 1H), 7.89 (dd, *J* = 8.5, 2.2 Hz, 1H), 7.67 (d, *J* = 8.4 Hz, 1H), 7.36 (d, *J* = 2.7 Hz, 1H), 7.29 (d, *J* = 8.7 Hz, 1H), 6.91 (d, *J* = 8.8 Hz, 1H), 4.25–4.18 (m, 2H), 3.80 (s, 3H), 3.23–3.13 (m, 2H), 2.35–2.24 (m, 2H). ^13^C NMR (100 MHz, CDCl_3_) δ 160.79, 159.68, 155.10, 147,89, 137.29, 135.63, 130.54, 128.93, 128.64, 128.57, 126.89, 126.15, 125.85, 112.48, 55.89, 46.65, 32.49, 19.53. HRMS (ESI) calcd for C_18_H_15_ClN_2_O_2_ [M+H]^+^ 327.0895, found 327.0905.

7-(Naphthalen-1-yl)-2,3-dihydropyrrolo[2,1-*b*]quinazolin-9(1*H*)-one (3m). Yield 87%, white solid, m.p: 169–170 °C. ^1^H NMR (400 MHz, CDCl_3_) δ8.43 (d, *J* = 2.0 Hz, 1H), 7.96–7.83 (m, 4H), 7.77 (d, *J* = 8.3 Hz, 1H), 7.61–7.39 (m, 4H), 4.52–3.73 (m, 2H), 3.24 (t, *J* = 8.0 Hz, 2H), 2.56–2.12 (m, 2H). ^13^C NMR (100 MHz, CDCl_3_) δ160.87, 159.66, 147.95, 139.05, 138.69, 136.28, 133.80, 131.38, 128.38, 128.17, 127.43, 127.35, 126.47, 126.36, 125.91, 125.53, 125.37, 120.44, 46.65, 32.53, 19.57. HRMS (ESI) calcd for C_21_H_16_N_2_O [M+H]^+^ 313.1335, found 313.1344.

7-Phenyl-2,3-dihydropyrrolo[2,1-*b*]quinazolin-9(1*H*)-one (3n). Yield 88%, white solid, m.p: 167–168 °C. ^1^H NMR (400 MHz, CDCl_3_) δ8.50 (d, *J* = 2.2 Hz, 1H), 7.98 (dd, *J* = 8.5, 2.2 Hz, 1H), 7.74–7.67 (m, 3H), 7.47 (t, *J* = 7.6 Hz, 2H), 7.38 (ddd, *J* = 8.4, 4.4, 1.7 Hz, 1H), 4.33–4.09 (m, 2H), 3.20 (t, *J* = 8.0 Hz, 2H), 2.49–2.23 (m, 2H). ^13^C NMR (100 MHz, CDCl_3_) δ 160.99, 159.41, 148.16, 139.59, 139.15, 133.08, 128.94, 127.75, 127.19, 127.14, 124.27, 120.70, 46.59, 32.51, 19.55. HRMS (ESI) calcd for C_17_H_14_N_2_O [M+H]^+^ 263.1179, found 263.1188.

2-(4-Chlorophenyl)-6,7,8,9-tetrahydro-11*H*-pyrido[2,1-*b*]quinazolin-11-one (4a). Yield 87%, white solid, m.p: 168–169 °C. ^1^H NMR (400 MHz, CDCl_3_) δ 8.44 (d, *J* = 1.8 Hz, 1H), 7.92 (dd, *J* = 8.5, 2.2 Hz, 1H), 7.61 (d, *J* = 8.2 Hz, 2H), 7.43 (d, *J* = 8.3 Hz, 3H), 4.10 (t, *J* = 6.3 Hz, 2H), 3.03 (t, *J* = 6.4 Hz, 2H), 2.09–1.91 (m, 4H). ^13^C NMR (100 MHz, CDCl_3_) δ 161.98, 155.32, 138.11, 137.80, 133.91, 132.96, 132.13, 132.03, 129.11, 128.54, 128.42, 128.35, 124.45, 120.54, 42.53, 31.77, 22.05, 19.21. HRMS (ESI) calcd for C_18_H_15_ClN_2_O [M+H]^+^ 311.0873, found 311.0956.

2-(4-Fluorophenyl)-6,7,8,9-tetrahydro-11*H*-pyrido[2,1-*b*]quinazolin-11-one (4b). Yield 86%, yellow solid, m.p: 133–134 °C. ^1^H NMR (400 MHz, CDCl_3_) δ 8.43 (d, *J* = 2.2 Hz, 1H), 7.91 (dd, *J* = 8.5, 2.2 Hz, 1H), 7.74–7.59 (m, 3H), 7.15 (t, *J* = 8.7 Hz, 2H), 4.10 (t, *J* = 6.2 Hz, 2H), 3.03 (t, *J* = 6.6 Hz, 2H), 2.09–1.91 (m, 4H). ^13^C NMR (100 MHz, CDCl_3_) δ 163.51, 162.01 (d, J = 42.9 Hz), 155.00, 146.40, 138.00, 135.89 (d, *J* = 3.2 Hz), 133.00, 128.76 (d, *J* = 8.1 Hz), 126.92, 124.35, 120.60, 115.86 (d, *J* = 21.4 Hz), 42.49, 31.92, 22.11, 19.31. HRMS (ESI) calcd for C_18_H_15_FN_2_O [M+H]^+^ 295.1241, found 295.1250.

2-(4-(Trifluoromethyl)phenyl)-6,7,8,9-tetrahydro-11*H*-pyrido[2,1-*b*]quinazolin-11-one (4c). Yield 89%, white solid, m.p: 206–207 °C. ^1^H NMR (400 MHz, CDCl_3_) δ 8.50 (d, *J* = 2.2 Hz, 1H), 7.97 (dd, *J* = 8.5, 2.2 Hz, 1H), 7.79 (d, *J* = 8.4 Hz, 2H), 7.72 (d, *J* = 8.2 Hz, 3H), 4.11 (t, *J* = 6.3 Hz, 2H), 3.04 (t, *J* = 6.7 Hz, 2H), 2.10–1.92 (m, 4H). ^13^C NMR (100 MHz, CDCl_3_) δ162.02, 155.57, 146.91, 143.18, 137.41, 133.05, 129.72 (q, *J* = 32.3 Hz), 127.38, 127.07, 125.89 (q, *J* = 3.6 Hz), 124.99, 124.81 (q, *J* = 272.3 Hz), 120.66, 42.55, 31.89, 22.05, 19.24. HRMS (ESI) calcd for C_19_H_15_F_3_N_2_O [M+H]^+^ 345.1209, found 345,1220.

2-(4-Methoxyphenyl)-6,7,8,9-tetrahydro-11*H*-pyrido[2,1-*b*]quinazolin-11-one (4d). Yield 72%, yellow solid, m.p: 126–127 °C. ^1^H NMR (400 MHz, CDCl_3_) δ 8.43 (d, *J* = 2.2 Hz, 1H), 7.93 (dd, *J* = 8.5, 2.3 Hz, 1H), 7.64 (t, *J* = 9.0 Hz, 3H), 6.99 (d, *J* = 8.8 Hz, 2H), 4.10 (t, *J* = 6.2 Hz, 2H), 3.85 (s, 3H), 3.02 (t, *J* = 6.6 Hz, 2H), 2.04–1.93 (m, 4H). ^13^C NMR (100 MHz, CDCl_3_) δ 162.21, 159.48, 154.59, 145.95, 138.64, 132.81, 128.42, 128.17, 126.72, 123.72, 120.56, 114.37, 55.36, 42.41, 31.86, 22.11, 19.31. HRMS (ESI) calcd for C_19_H_18_N_2_O_2_ [M+H]^+^ 307.1441, found 307.1450.

2-(4-(*tert*-Butyl)phenyl)-6,7,8,9-tetrahydro-11*H*-pyrido[2,1-*b*]quinazolin-11-one (4e). Yield 74%, yellow solid, m.p: 161–162 °C. ^1^H NMR (400 MHz, CDCl_3_) δ 8.49 (d, *J* = 2.2 Hz, 1H), 7.98 (dd, *J* = 8.5, 2.2 Hz, 1H), 7.72–7.58 (m, 3H), 7.49 (d, *J* = 8.4 Hz, 2H), 4.10 (t, *J* = 6.2 Hz, 2H), 3.03 (t, *J* = 6.6 Hz, 2H), 2.08–1.90 (m, 4H), 1.37 (s, 9H). ^13^C NMR (100 MHz, CDCl_3_) δ 162.37, 155.06, 151.08, 139.11, 136.92, 134.51, 133.34, 126.96, 126.81, 126.14, 124.41, 120.73, 42.67, 34.81, 32.01, 31.54, 22.31, 19.49. HRMS (ESI) calcd for C_22_H_24_N_2_O [M+H]^+^ 333.1961, found 333.1971.

2-(m-Tolyl)-6,7,8,9-tetrahydro-11*H*-pyrido[2,1-*b*]quinazolin-11-one (4f). Yield 77%, yellow solid, m.p: 129–130 °C. ^1^H NMR (400 MHz, CDCl_3_) δ 8.48 (d, *J* = 2.2 Hz, 1H), 7.97 (dd, *J* = 8.5, 2.2 Hz, 1H), 7.67 (d, *J* = 8.5 Hz, 1H), 7.54–7.46 (m, 2H), 7.35 (t, *J* = 7.6 Hz, 1H), 7.19 (d, *J* = 7.5 Hz, 1H), 4.11 (t, *J* = 6.2 Hz, 2H), 3.03 (t, *J* = 6.6 Hz, 2H), 2.43 (s, 3H), 2.09–1.91 (m, 4H). ^13^C NMR (100 MHz, CDCl_3_) δ 162.21, 154.80, 146.48, 139.65, 139.10, 138.56, 133.18, 128.81, 128.43, 127.92, 126.74, 124.43, 124.18, 120.54, 42.41, 31.90, 22.11, 21.50, 19.32. HRMS (ESI) calcd for C_19_H_18_N_2_O [M+H]^+^ 291.1492, found 291.1500.

2-(3-Chlorophenyl)-6,7,8,9-tetrahydro-11*H*-pyrido[2,1-*b*]quinazolin-11-one (4g). Yield 80%, yellow solid, m.p: 124–125 °C. ^1^H NMR (400 MHz, CDCl_3_) δ 8.45 (d, *J* = 2.2 Hz, 1H), 7.93 (dd, *J* = 8.5, 2.3 Hz, 1H), 7.71–7.64 (m, 2H), 7.56 (d, *J* = 7.6 Hz, 1H), 7.43–7.30 (m, 2H), 4.10 (t, *J* = 6.3 Hz, 2H), 3.03 (t, *J* = 6.7 Hz, 2H), 2.09–1.91 (m, 4H). ^13^C NMR (100 MHz, CDCl_3_) δ 162.07, 155.25, 146.85, 141.54, 137.45, 134.87, 132.92, 130.14, 127.67, 127.24, 127.05, 125.23, 124.68, 120.63, 42.48, 31.94, 22.08, 19.29. HRMS (ESI) calcd for C_18_H_15_ClN_2_O [M+H]^+^ 311.0946, found 311.0955.

2-(3-Methoxyphenyl)-6,7,8,9-tetrahydro-11*H*-pyrido[2,1-*b*]quinazolin-11-one (4h). Yield 73%, yellow solid, m.p: 167–168 °C. ^1^H NMR (400 MHz, CDCl_3_) δ 8.46 (d, *J* = 2.2 Hz, 1H), 7.95 (dd, *J* = 8.5, 2.2 Hz, 1H), 7.66 (d, *J* = 8.5 Hz, 1H), 7.36 (t, *J* = 8.0 Hz, 1H), 7.26 (d, *J* = 6.0 Hz, 1H), 7.22–7.17 (m, 1H), 6.94–6.87 (m, 1H), 4.09 (t, *J* = 6.2 Hz, 2H), 3.87 (s, 3H), 3.01 (t, *J* = 6.6 Hz, 2H), 2.07–1.89 (m, 4H). ^13^C NMR (100 MHz, CDCl_3_) δ 162.15, 160.05, 154.97, 146.41, 141.16, 138.85, 133.24, 129.93, 126.71, 124.53, 120.48, 119.60, 113.34, 112.59, 55.36, 42.47, 31.86, 22.08, 19.27. HRMS (ESI) calcd for C_19_H_18_N_2_O_2_ [M+H]^+^ 307.1441, found 307.1450.

2-(3-Acetylphenyl)-6,7,8,9-tetrahydro-11*H*-pyrido[2,1-*b*]quinazolin-11-one (4i). Yield 86%, white solid, m.p: 126–127 °C. ^1^H NMR (400 MHz, CDCl_3_) δ 8.50 (d, *J* = 2.2 Hz, 1H), 8.25 (d, *J* = 1.9 Hz, 1H), 7.97 (dd, *J* = 15.3, 8.1 Hz, 2H), 7.88 (d, *J* = 7.7 Hz, 1H), 7.70 (dd, *J* = 8.7, 4.3 Hz, 1H), 7.57 (t, *J* = 7.8 Hz, 1H), 4.11 (t, *J* = 6.3 Hz, 2H), 3.04 (t, *J* = 6.7 Hz, 2H), 2.67 (s, 3H), 2.09–1.91 (m, 4H). ^13^C NMR (100 MHz, CDCl_3_) δ 197.91, 162.04, 155.37, 146.59, 140.25, 137.95, 137.78, 133.15, 131.68, 129.25, 127.57, 126.96, 126.88, 124.73, 120.60, 42.53, 31.85, 26.79, 22.06, 19.24. HRMS (ESI) calcd for C_20_H_18_N_2_O_2_ [M+H]^+^ 319.1441, found 319.1451.

2-(o-Tolyl)-6,7,8,9-tetrahydro-11*H*-pyrido[2,1-*b*]quinazolin-11-one (4j). Yield 71%, white solid, m.p: 178–179 °C. ^1^H NMR (400 MHz, CDCl_3_) δ 8.22 (d, *J* = 2.0 Hz, 1H), 7.72–7.61 (m, 2H), 7.32–7.21 (m, 4H), 4.09 (t, *J* = 6.1 Hz, 2H), 3.02 (t, *J* = 6.6 Hz, 2H), 2.28 (s, 3H), 2.08–1.90 (m, 4H). ^13^C NMR (100 MHz, CDCl_3_) 162.14, 154.88, 146.03, 140.57, 139.97, 135.51, 135.32, 130.44, 129.86, 127.65, 126.74, 126.00, 125.91, 120.09, 42.39, 31.89, 22.11, 20.46, 19.32. HRMS (ESI) calcd for C_19_H_18_N_2_O [M+H]^+^ 291.1492, found 291.1500.

2-(3-Chloro-4-fluorophenyl)-6,7,8,9-tetrahydro-11*H*-pyrido[2,1-*b*]quinazolin-11-one (4k). Yield 85%, white solid, m.p: 167–168 °C. ^1^H NMR (400 MHz, CDCl_3_) δ 8.40 (d, *J* = 2.2 Hz, 1H), 7.88 (dd, *J* = 8.5, 2.3 Hz, 1H), 7.74–7.61 (m, 2H), 7.52 (ddd, *J* = 8.6, 4.5, 2.3 Hz, 1H), 7.22 (t, *J* = 8.7 Hz, 1H), 4.10 (t, *J* = 6.2 Hz, 2H), 3.03 (t, *J* = 6.6 Hz, 2H), 2.09–1.91 (m, 4H). ^13^C NMR (100 MHz, CDCl_3_) δ 161.96, 157.87 (d, *J* = 250.0 Hz), 155.41, 146.52, 136.96 (d, *J* = 3.8 Hz), 136.68, 132.80, 129.25, 126.99, 126.79 (d, *J* = 7.2 Hz), 124.51, 121.53 (d, *J* = 17.9 Hz), 120.57, 117.01 (d, *J* = 21.2 Hz), 42.56, 31.86, 22.04, 19.23. HRMS (ESI) calcd for C_18_H_14_ClFN_2_O [M+H]^+^ 329.0851, found 329.0862.

2-(5-Chloro-2-methoxyphenyl)-6,7,8,9-tetrahydro-11*H*-pyrido[2,1-*b*]quinazolin-11-one (4l). Yield 83%, yellow solid, m.p: 149–150 °C. ^1^H NMR (400 MHz, cdcl_3_) δ 8.37 (d, *J* = 2.1 Hz, 1H), 7.88 (dd, *J* = 8.5, 2.1 Hz, 1H), 7.62 (d, *J* = 8.5 Hz, 1H), 7.36 (d, *J* = 2.6 Hz, 1H), 7.28 (dd, *J* = 8.8, 2.6 Hz, 1H), 6.91 (d, *J* = 8.8 Hz, 1H), 4.09 (t, *J* = 6.1 Hz, 2H), 3.80 (s, 3H), 3.02 (t, *J* = 6.6 Hz, 2H), 2.07–1.90 (m, 4H). ^13^C NMR (100 MHz, CDCl_3_) δ 162.04, 155.10, 146.30, 135.63, 135.25, 130.75, 130.53, 128.55, 127.10, 125.89 125.81, 120.10, 112.46, 55.88, 42.40, 31.85, 22.09, 19.28. HRMS (ESI) calcd for C_19_H_17_ClN_2_O_2_ [M+H]^+^ 341.1051, found 341.1063.

2-(Naphthalen-1-yl)-6,7,8,9-tetrahydro-11*H*-pyrido[2,1-*b*]quinazolin-11-one (4m). Yield 81%, white solid, m.p: 130–131 °C. ^1^H NMR (400 MHz, CDCl_3_) δ 8.41 (d, *J* = 2.0 Hz, 1H), 7.96–7.82 (m, 4H), 7.75 (d, *J* = 8.3 Hz, 1H), 7.58–7.39 (m, 4H), 4.12 (t, *J* = 6.1 Hz, 2H), 3.08 (t, *J* = 6.5 Hz, 2H), 2.10–1.93 (m, 4H). ^13^C NMR (100 MHz, CDCl_3_) 162.05, 155.22, 146.16, 138.89, 138.79, 136.37, 133.79, 131.38, 128.38, 128.13, 127.64, 127.34, 126.34, 126.03, 125.91, 125.56, 125.37, 120.28, 42.48, 31.82, 22.10, 19.28. HRMS (ESI) calcd for C_22_H_18_N_2_O [M+H]^+^ 327.1492, found 327.1501.

2-(2-(Trifluoromethyl)phenyl)-6,7,8,9-tetrahydro-11*H*-pyrido[2,1-*b*]quinazolin-11-one (4n). Yield 96%, brown solid, m.p: 130–131 °C. ^1^H NMR (400 MHz, CDCl_3_) δ 8.21 (d, *J* = 2.0 Hz, 1H), 7.76 (d, *J* = 7.2 Hz, 1H), 7.72–7.60 (m, 2H), 7.58 (t, *J* = 7.2 Hz, 1H), 7.50 (t, *J* = 7.6 Hz, 1H), 7.36 (d, *J* = 7.5 Hz, 1H), 4.09 (t, *J* = 6.1 Hz, 2H), 3.04 (t, *J* = 6.6 Hz, 2H), 2.08–1.91 (m, 4H). ^13^C NMR (100 MHz, CDCl_3_) δ 162.18, 155.63, 146.62, 140.21 (q, *J* = 3.6 Hz), 138.04, 135.34 (q, *J* = 3.4 Hz), 132.29, 131.65, 127.99, 127.04, 127.02, 126.39 (q, *J* = 5.3 Hz), 125.84, 124.23 (q, *J* = 274.3 Hz), 120.00, 42.70, 32.06, 22.28, 19.47. HRMS (ESI) calcd for C_19_H_15_F_3_N_2_O [M+H]^+^ 345,1209, found 345.1220.

### 3.3. Biological Assay Methods

#### AChE and BChE Inhibition Assays

The total volume of the reaction was 200 μL, consisting of 2 μL of sample, 138 μL of Tris-HCl buffer solution containing acetylcholinesterase (final concentration of 0.03 U/mL), and a control consisting of Tris-HCl buffer solution without the enzyme. The reaction was incubated at room temperature for 10 min. Following this, 20 μL of thioethane (butyryl)choline iodide (5 mM) was added, and the reaction was conducted at 37 °C for an additional 10 min. The absorbance was measured at 405 nm for each well. After the 10-min incubation at 37 °C, 20 μL of 1% SDS and 20 μL of DTNB (2.5 mM) were added to each well, and the absorbance at 405 nm was determined by shaking and mixing the solution.

### 3.4. Molecular Docking

The crystal structure of the protein was obtained from the Protein Data Bank (PDB: 4EY7). The proteins underwent hydrogenation and dehydrogenation, followed by structure and energy optimization. The protonation of ligand **3k** was conducted at pH 7 ± 0.4 using the ‘Ligand Preparation’ module, and minimization was performed using the ‘Ligand Minimization’ module. Finally, molecular docking was executed using AutoDock 4 software, and PyMOL was utilized for visualization and analysis of the results [70].

#### Molecular Dynamics Analysis 

Molecular dynamics simulations were conducted using GROMACS 2025. The Amber99SB molecular force field was selected, and the TIP3P water model was employed. The cut-off distances were uniformly set to 10 pm, and the time step was established at 2 fs. Long-range electrostatic interactions were corrected using the Particle-Mesh Ewald (PME) method. The simulation conditions included a temperature of 300 K and a pressure of 1 bar. Subsequently, an appropriate amount of sodium ions was added to neutralize the charge of the simulated system. Energy minimization was performed using the steepest descent method, followed by NVT and NPT equilibrium simulations, each lasting 100 ps. The system temperature was coupled to a heat bath using the V-rescale method, while pressure was controlled using the Parrinello–Rahman method. Finally, molecular dynamics simulations were executed, comprising 50,000,000 steps with a step size of 2 fs, resulting in a total duration of 100 ns. At the conclusion of the calculations, the root mean square deviation (RMSD), root mean square fluctuation (RMSF), radius of gyration (Rg), and hydrogen bonding were analyzed using the software’s built-in tools [70].

## 4. Conclusions

In conclusion, this scientific investigation presents a study of the design, synthesis, and biological evaluation of 28 novel 7-phenyl-2,3-dihydro-pyrrolo[2,1-*b*]quinazolin-9(1*H*)-one derivatives. The research focused on their activity, particularly their ability to inhibit AChE, an enzyme associated with AD. Notably, compound **3k** was the most effective, with an IC_50_ value of 6.084 ± 0.26 µM, indicating its strong inhibitory potential. Among the tetra-methylene series, derivative **4f** showed the highest activity toward the AChE and BChE, with IC_50_ values of 6.39 ± 0.31 and 10.74 ± 0.64, respectively. Molecular docking studies revealed that compound **3k** interacted with AChE (4EY7) through critical hydrogen bonds and π-π stacking interactions, suggesting stable binding. Overall, compound **3k** shows significant promise as a therapeutic agent for Alzheimer’s disease due to its potent inhibitory activity and stable interactions with AChE.

## Figures and Tables

**Table 1:** Optimization of conditions for the Suzuki coupling reaction.

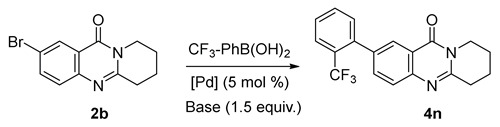						
No.	Catalyst ^a^	Base ^b^	Solvent	Temperature	Time	Yield (%)
1	PdCl_2_(PPh_3_)_2_	K_2_CO_3_	PhCH_3_	110 °C	8 h	48
2	Pd(AcO)_2_	K_2_CO_3_	PhCH_3_	110 °C	8 h	45
3	Pd_2_(DBA)_3_	K_2_CO_3_	PhCH_3_	110 °C	8 h	38
4	Pd(PPh_3_)_4_	K_2_CO_3_	PhCH_3_	110 °C	8 h	56
5	Pd(PPh_3_)_4_	Cs_2_CO_3_	PhCH_3_	110 °C	8 h	61
6	Pd(PPh_3_)_4_	*t*-BuOK	PhCH_3_	110 °C	8 h	51
7	Pd(PPh_3_)_4_	K_2_CO_3_	1,4-dioxane	100 °C	8 h	26
8	Pd(PPh_3_)_4_	Cs_2_CO_3_	1,4-dioxane	100 °C	8 h	32
9	Pd(PPh_3_)_4_	*t*-BuOK	1,4-dioxane	100 °C	8 h	24
10	Pd(PPh_3_)_4_	Cs_2_CO_3_	PhCH_3_:H_2_O:EtOH = 1:1:1	90 °C	8 h	62
11	Pd(PPh_3_)_4_	Cs_2_CO_3_	PhCH_3_:H_2_O = 3:1	110 °C	8 h	96
12	Pd(PPh_3_)_4_	Cs_2_CO_3_	PhCH_3_:H_2_O = 5:1	110 °C	8 h	70

^a^—catalyst used at 5.0 mol% relative to the starting compound. ^b^—base used at 1.5 equivalents relative to the starting compound.

**Table 2:** ChEs inhibition and cytotoxicity of the compounds **3a**–**3n** and **4a**–**4n**.

No.	IC_50_^c^, μM		Selectivity (BChE/AChE) ^d^	No.	IC_50_^c^, μM		Selectivity (BChE/AChE) ^d^
	AChE ^a^	BChE ^b^			AChE ^a^	BChE ^b^	
**3a**	>50	>50	– ^e^	**4a**	>50	>50	–
**3b**	34.76 ± 1.21	15.52 ± 0.41	0.5	**4b**	34.59 ± 1.27	>50	–
**3c**	>50	>50	–	**4c**	>50	>50	–
**3d**	16.21 ± 0.39	>50	–	**4d**	33.24 ± 1.08	>50	–
**3e**	>50	>50	–	**4e**	>50	>50	–
**3f**	28.71 ± 0.69	22.79 ± 0.49	0.8	**4f**	6.39 ± 0.31	10.74 ± 0.64	1.7
**3g**	27.92 ± 0.72	22.2 ± 0.48	0.8	**4g**	21.28 ± 0.88	22.93 ± 0.76	1.1
**3h**	17.7 ± 0.84	41.84 ± 1.28	2.4	**4h**	42.67 ± 1.80	42.3 ± 1.23	1
**3i**	33.18 ± 1.01	>50	–	**4i**	11.12 ± 0.73	>50	–
**3j**	>50	11.06 ± 0.37	–	**4j**	>50	>50	–
**3k**	6.084 ± 0.26	29.01 ± 0.74	4.8	**4k**	>50	>50	–
**3l**	14.74 ± 0.48	28.53 ± 0.46	2.0	**4l**	>50	46.61 ± 1.45	–
**3m**	>50	31.98 ± 0.72	–	**4m**	>50	>50	–
**3n**	35.86 ± 1.03	22.94 ± 0.57	0.64	**4n**	>50	43.19 ± 1.27	–
Huperzine A	0.25 ± 0.01	–	–		0.25 ± 0.01	–	–
Tacrine	0.037 ± 0.00	5.24 ± 0.02	–		0.037 ± 0.00	5.24 ± 0.02	–

^a^ AChE (E.C. 3.1.1.7) fromelectric eel; ^b^ BChE (E.C. 3.1.1.8) fromhorse serum; ^c^ Concentration required for 50% inhibition of ChEs, data are shown in means ± SD of triplicate independent experiments; ^d^ Selectivity = BChE IC_50_/AChE IC_50_ (BChE-to-AChE IC_50_ ratio, calculated as IC_50_(BChE)/IC_50_(AChE)); ^e^ not determined.

## Data Availability

The data that supports the structure confirmation of this study are available in the Supplementary Material of this article.

## Supplementary Materials

The following supporting information can be downloaded at: https://www.mdpi.com/article/10.3390/molecules30132791/s1, Figures S1–S84: The ^1^H and ^13^C NMR, along with the HRMS spectrum of compounds **3a–3n** and **4a–4n**.
