# Supplementary material for: Synthesis of Novel 7-Phenyl-2,3-Dihydropyrrolo[2,1-b]Quinazolin-9(1H)-ones as Cholinesterase Inhibitors Targeting Alzheimer’s Disease Through Suzuki–Miyaura Cross-Coupling Reaction
Source: Molecules. 2025 Jun 28;30(13):2791. doi: 10.3390/molecules30132791 (PMC12250785; doi:10.3390/molecules30132791)
Supplement: Supplementary file 1 [file molecules-30-02791-s001.zip › molecules-3714342-supplementary.pdf]

# SUPPLEMENTARY MATERIALS

# Synthesis of Novel 7-Phenyl-2,3-Dihydropyrrolo[2,1-*b*]Quinazolin-9(1*H*)-ones as Cholinesterase Inhibitors Targeting Alzheimer's Disease Through Suzuki-Miyaura Cross-Coupling Reaction

**Davron Turgunov [1], Lifei Nie [2], Azizbek Nasrullaev [2], Bianlin Wang [2], Zarifa Murtazaeva [1], Dilafruz Kholmurodova [3], Rustamkhon Kuryazov [4], Jiangyu Zhao [2], Khurshed Bozorov [1,2,*] and Haji Akber Aisa [2,*]**

1 Department of Organic Synthesis and Bioorganic Chemistry, Institute of Biochemistry, Samarkand State University, University Blvd. 15, Samarkand, 140104, Uzbekistan
2 State Key Laboratory Basis of Xinjiang Indigenous Medicinal Plants Resource Utilization, Xinjiang Technical Institute of Physics and Chemistry, Chinese Academy of Sciences, South Beijing Rd 40-1, Urumqi, 830011, P.R. China
3 Scientific and Practical Center of Immunology, Allergology and Human Genomics, Samarkand State Medical University, Makhdum-i Aʿẓam st. 18, Samarkand, 140104, Uzbekistan
4 Department of Chemistry, Urgench State University, Kh. Olimjon st. 14, Urgench, 220100, Uzbekistan
* Correspondence: khurshedbek@gmail.com; haji@ms.xjb.ac.cn

The $^{1}$H and $^{13}$C NMR, along with the HRMS spectrum of compounds 3a-3n and 4a-4n.

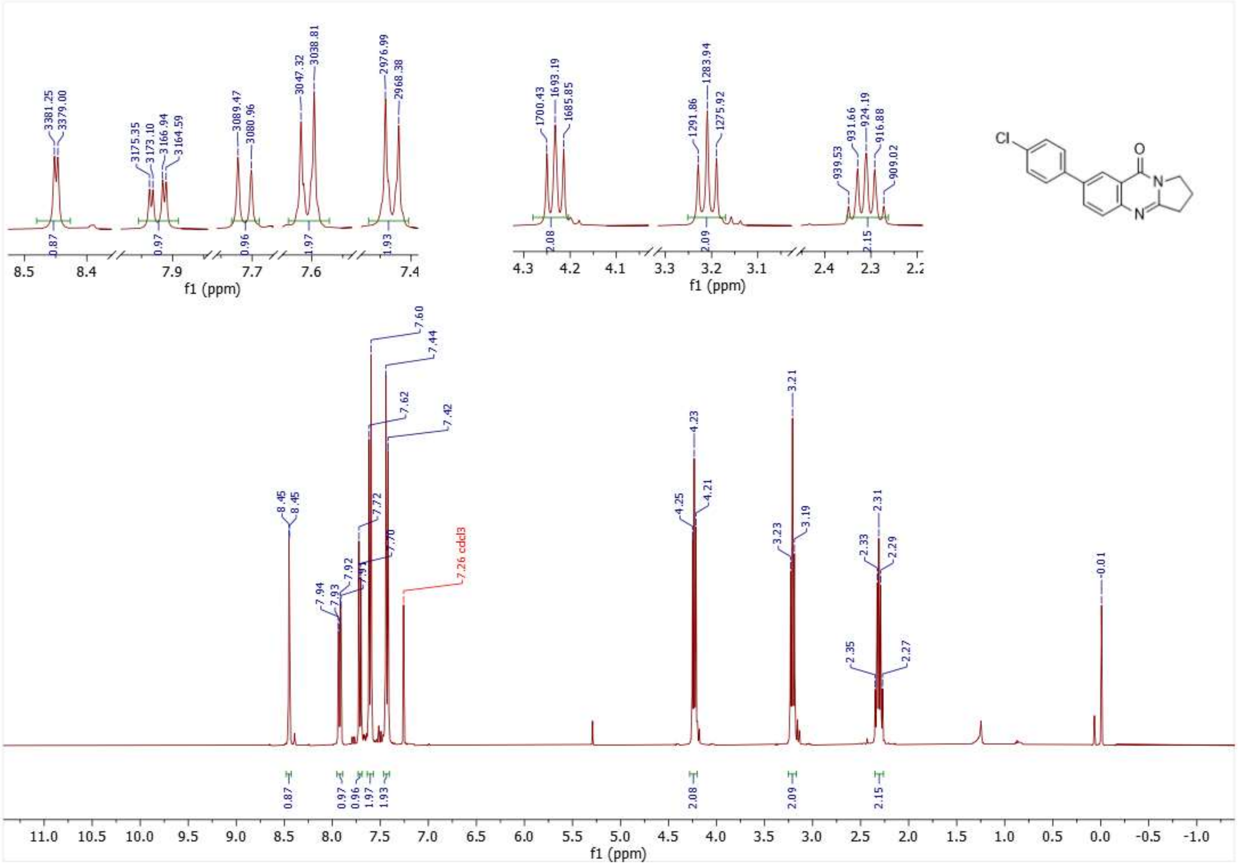

**Figure S1. ¹H NMR spectrum of 3a**

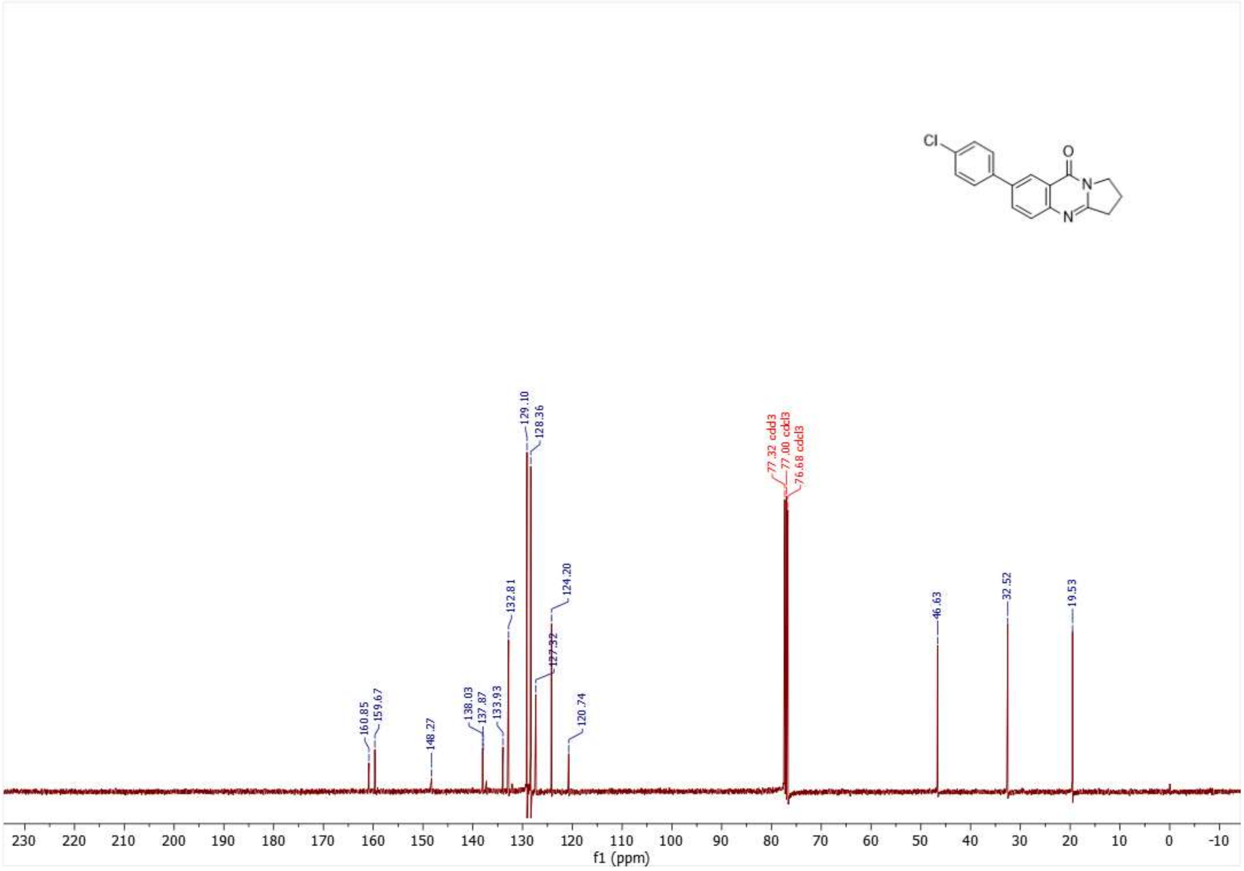

**Figure S2. ¹³C NMR spectrum of 3a**

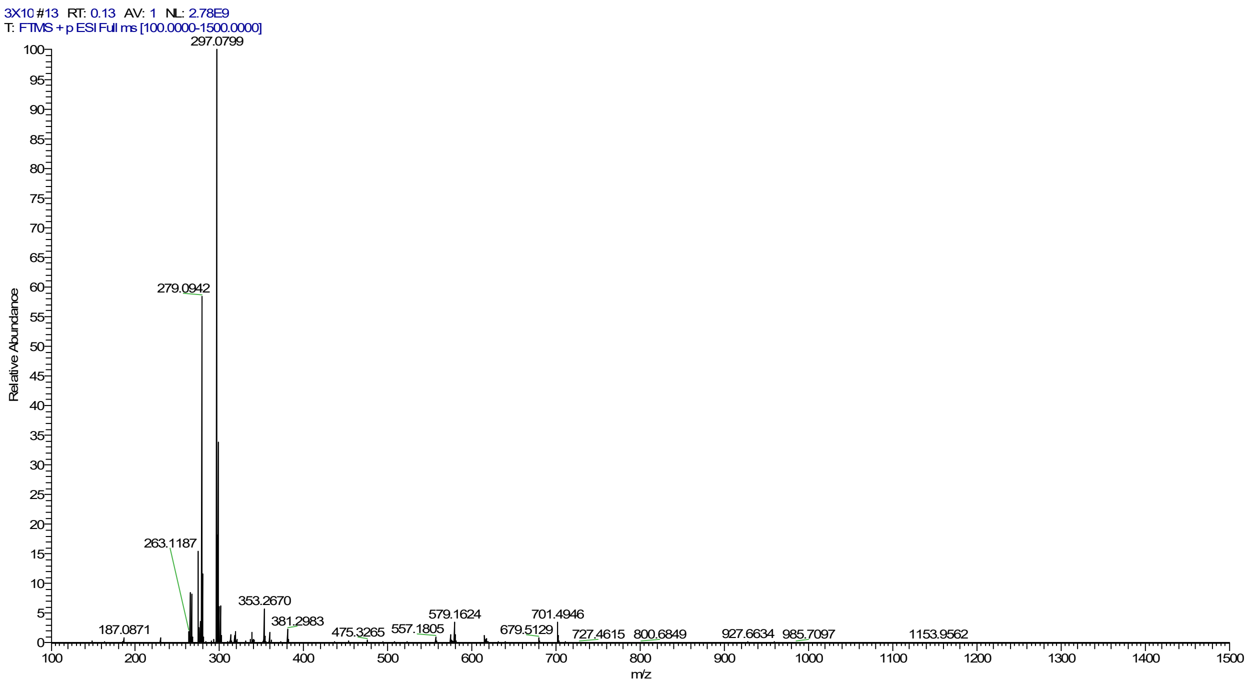


**Figure S3. Mass spectrum of 3a**

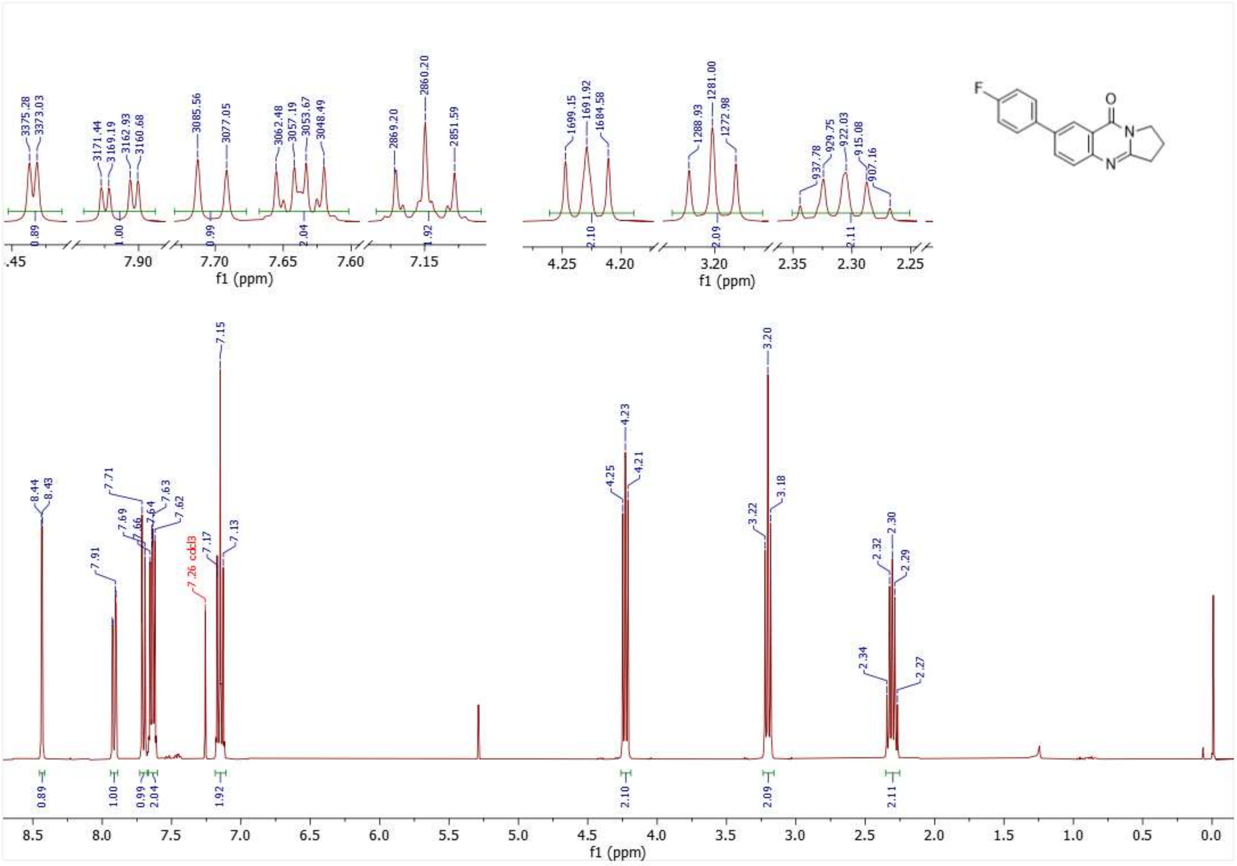


**Figure S4. $^{1}$H NMR spectrum of 3b**

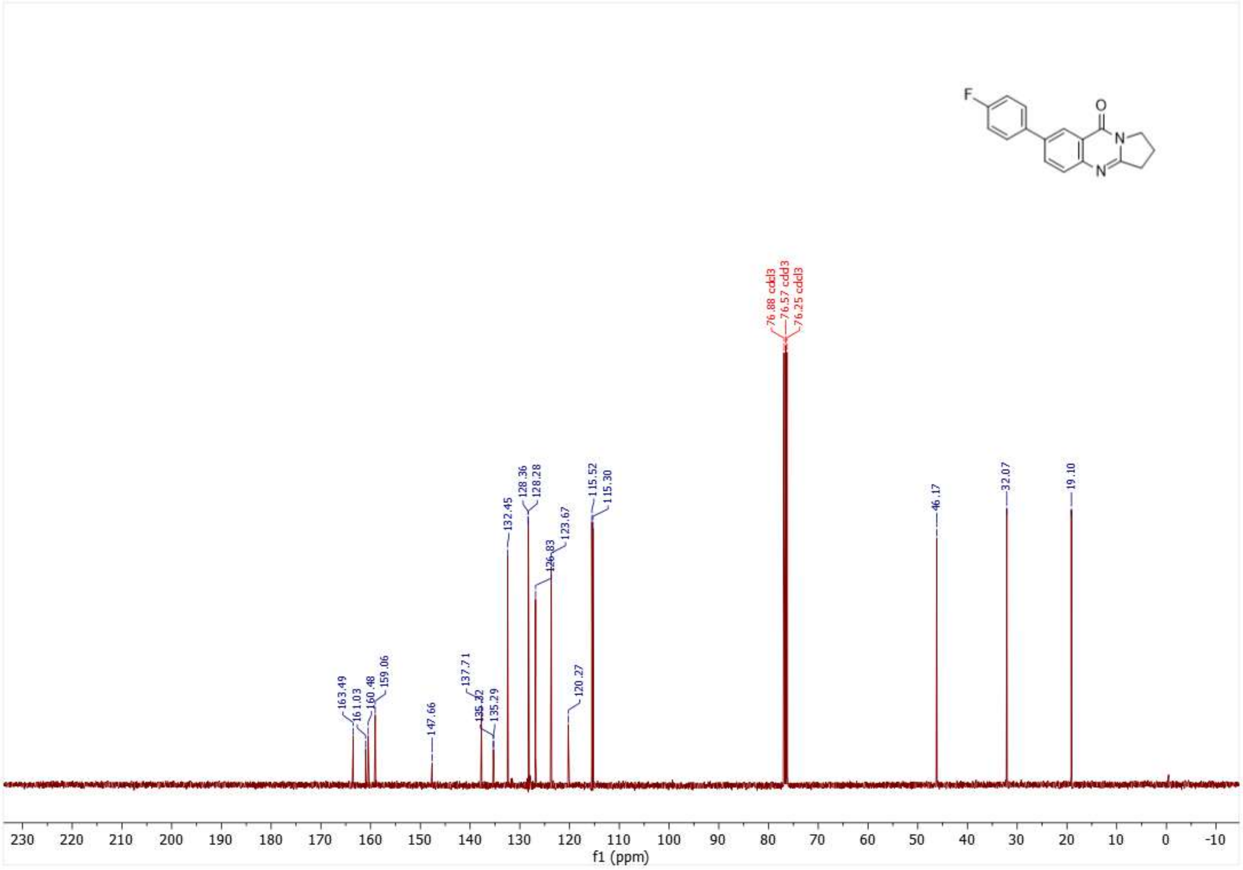

# Figure S5. $^{13}$C NMR spectrum of 3b

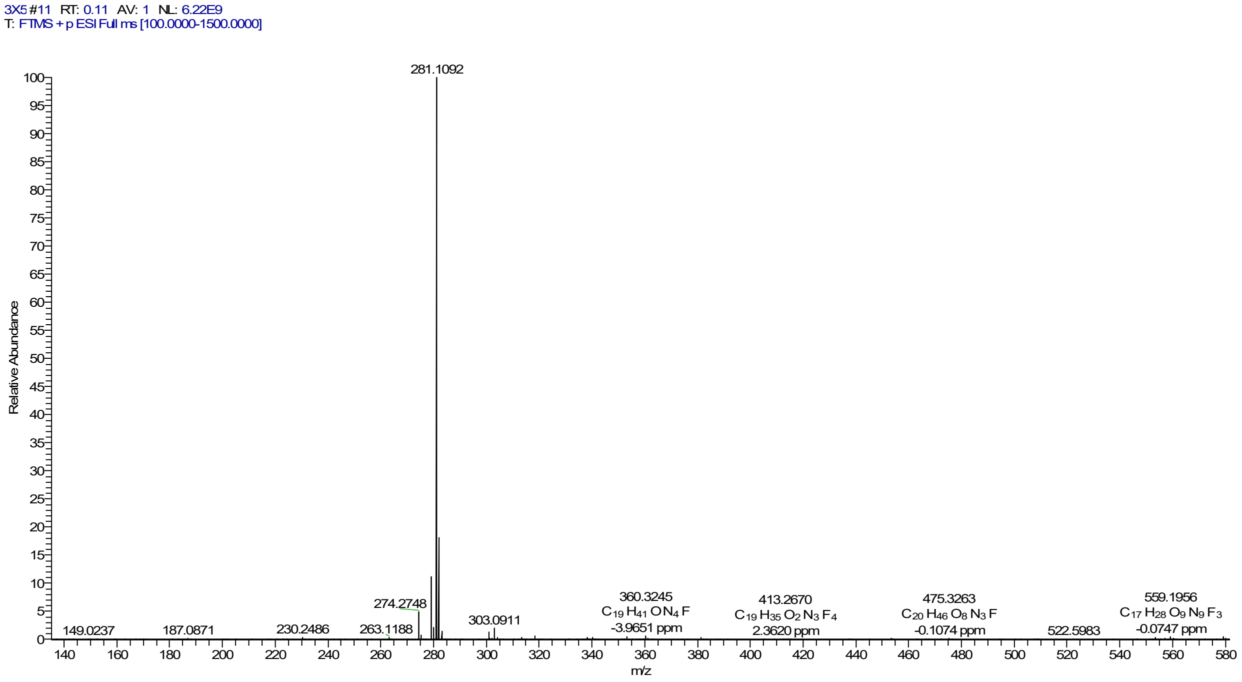

# Figure S6. Mass spectrum of 3b

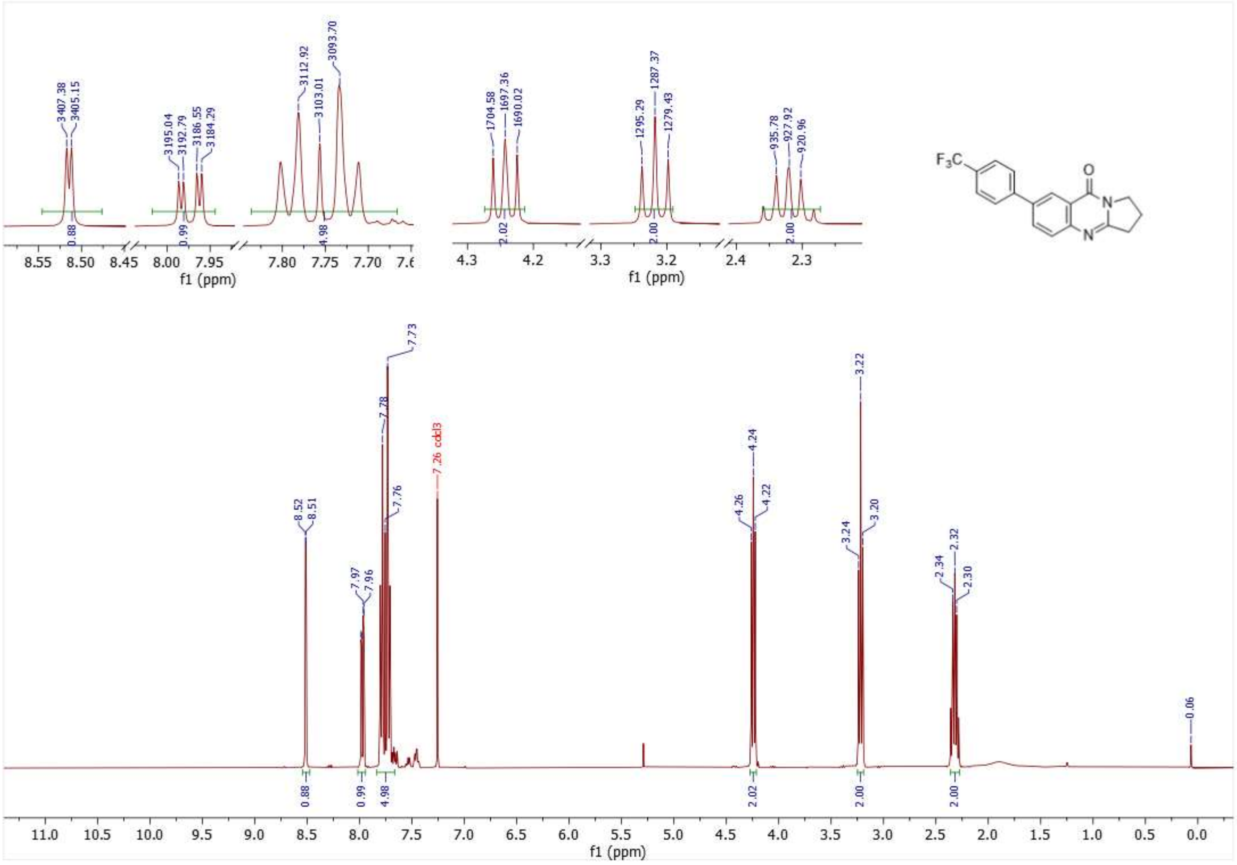

**Figure S7. $^{1}$H NMR spectrum of 3c**

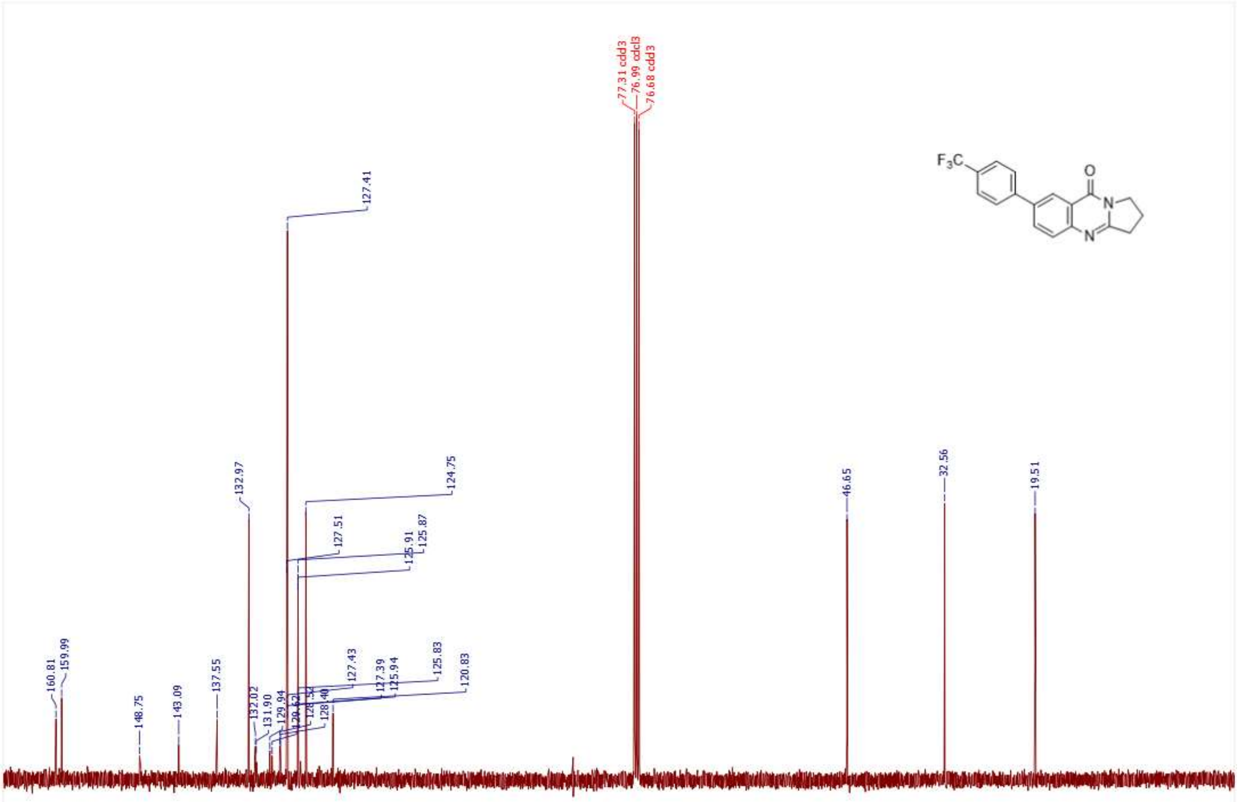

**Figure S8. $^{13}$C NMR spectrum of 3c**

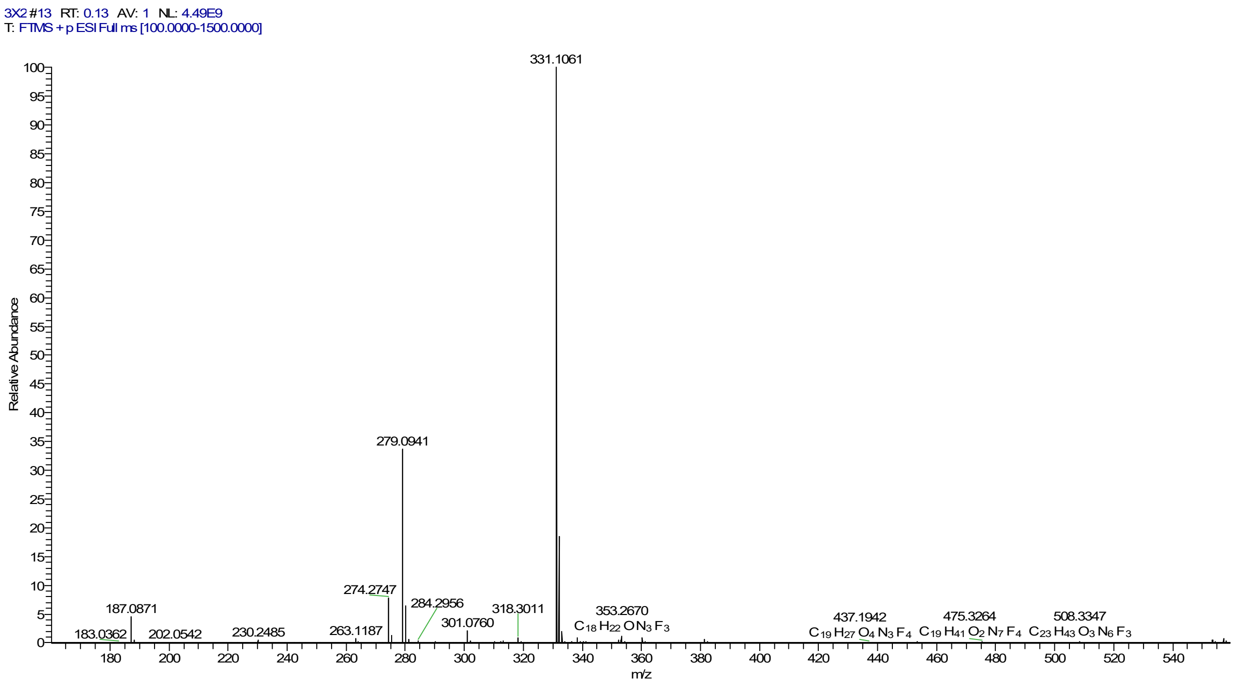


**Figure S9. Mass spectrum of 3c**

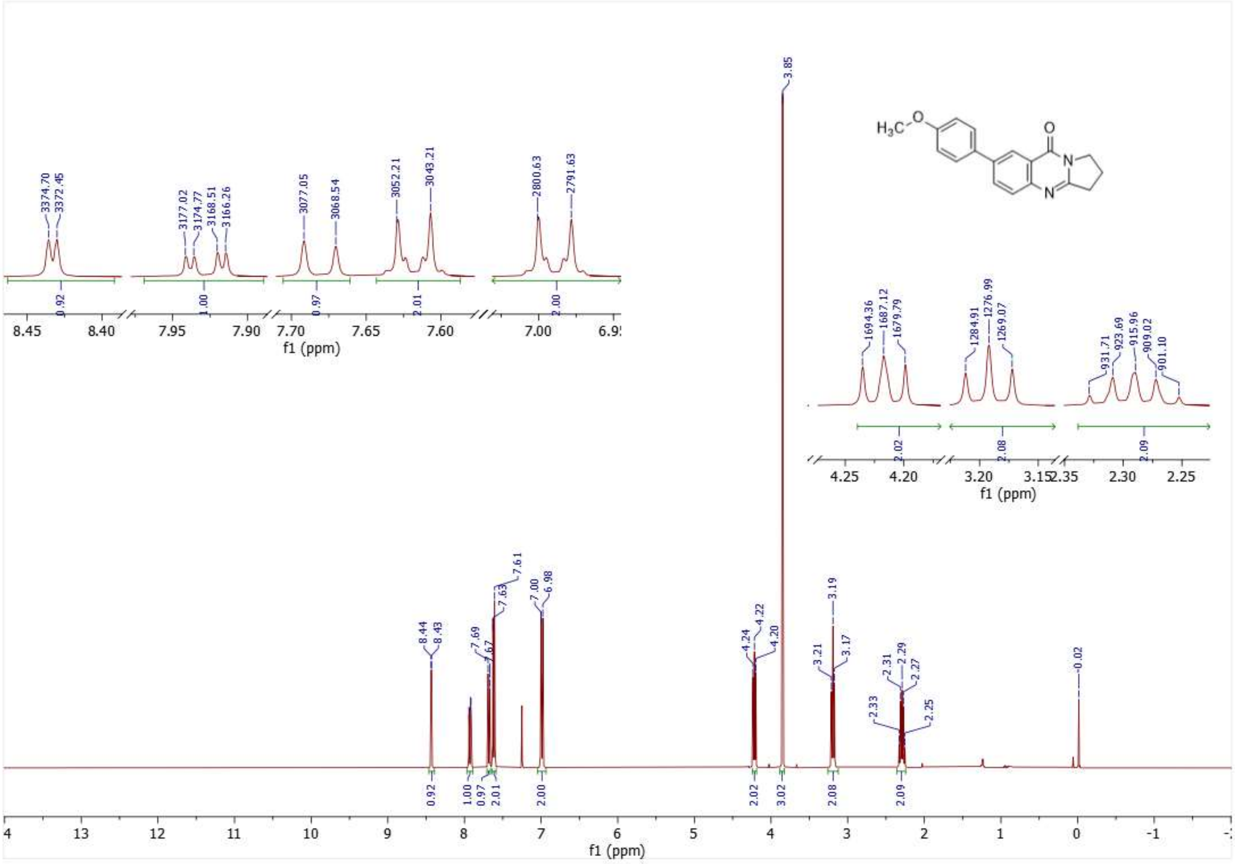


**Figure S10. $^{1}$H NMR spectrum of 3d**

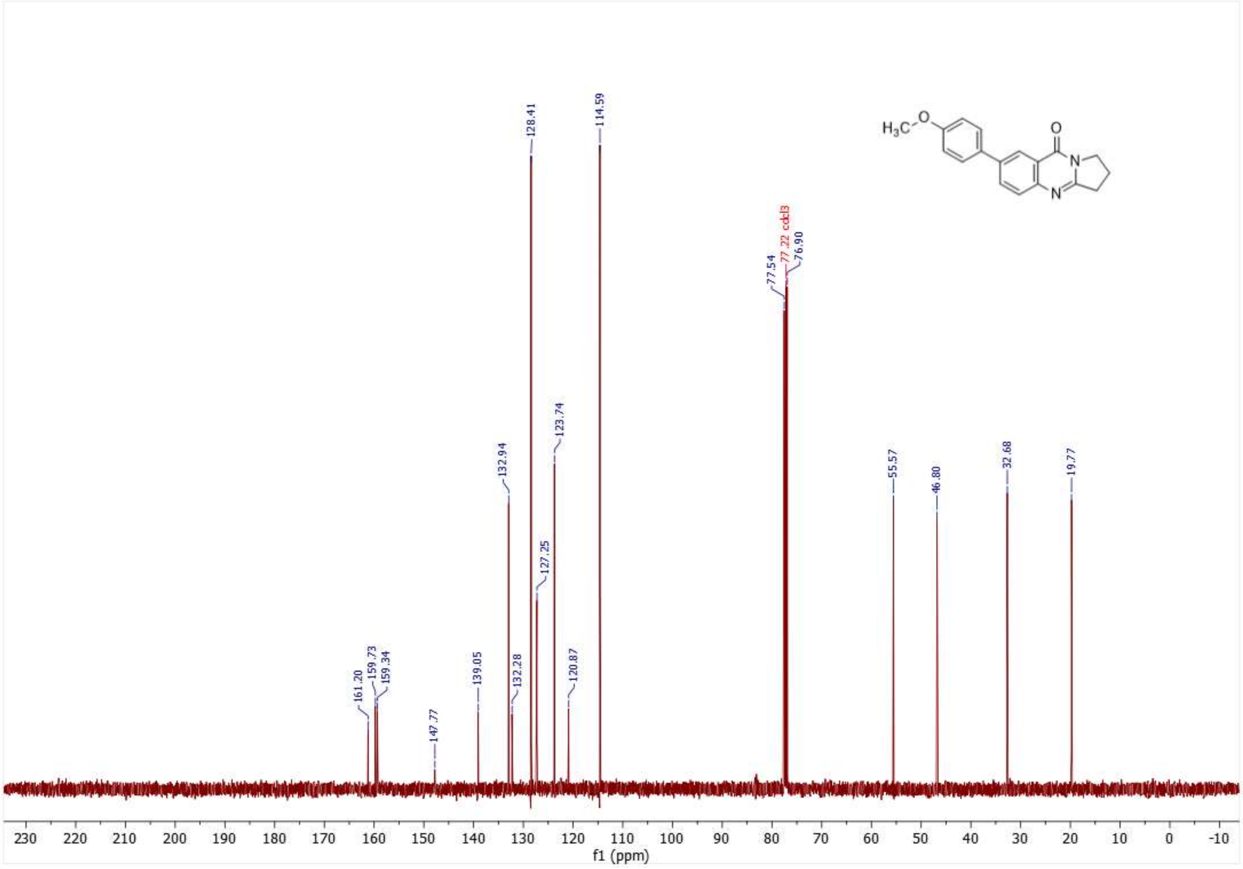


**Figure S11.** $^{13}$**C NMR spectrum of 3d**

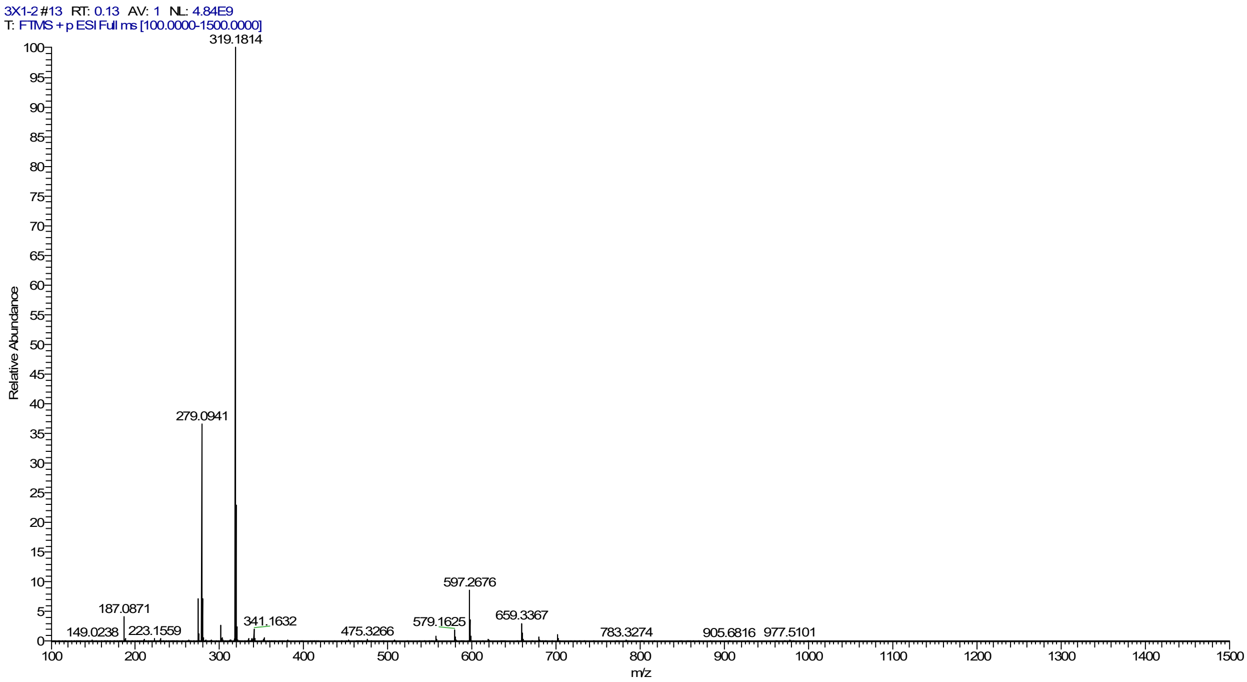


**Figure S12. Mass spectrum of 3d**

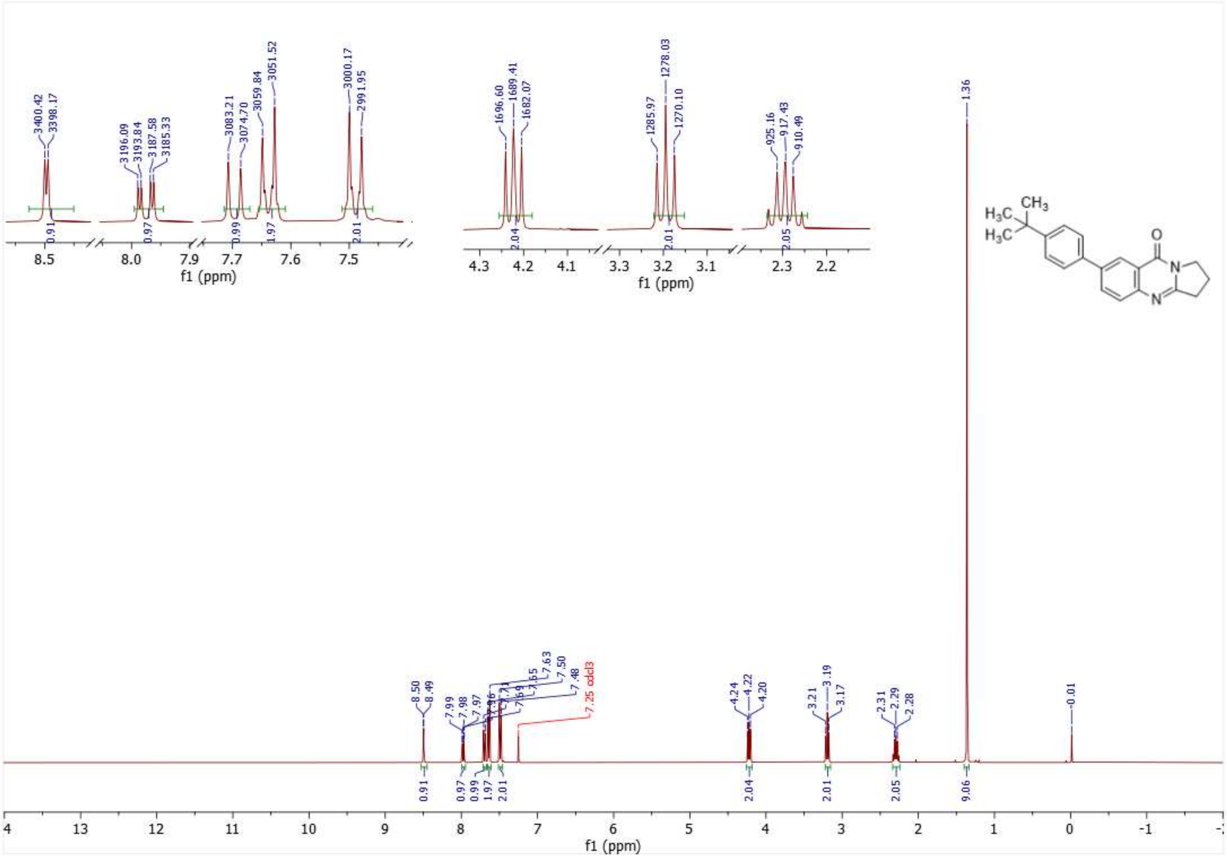

**Figure S13. $^{1}$H NMR spectrum of 3e**

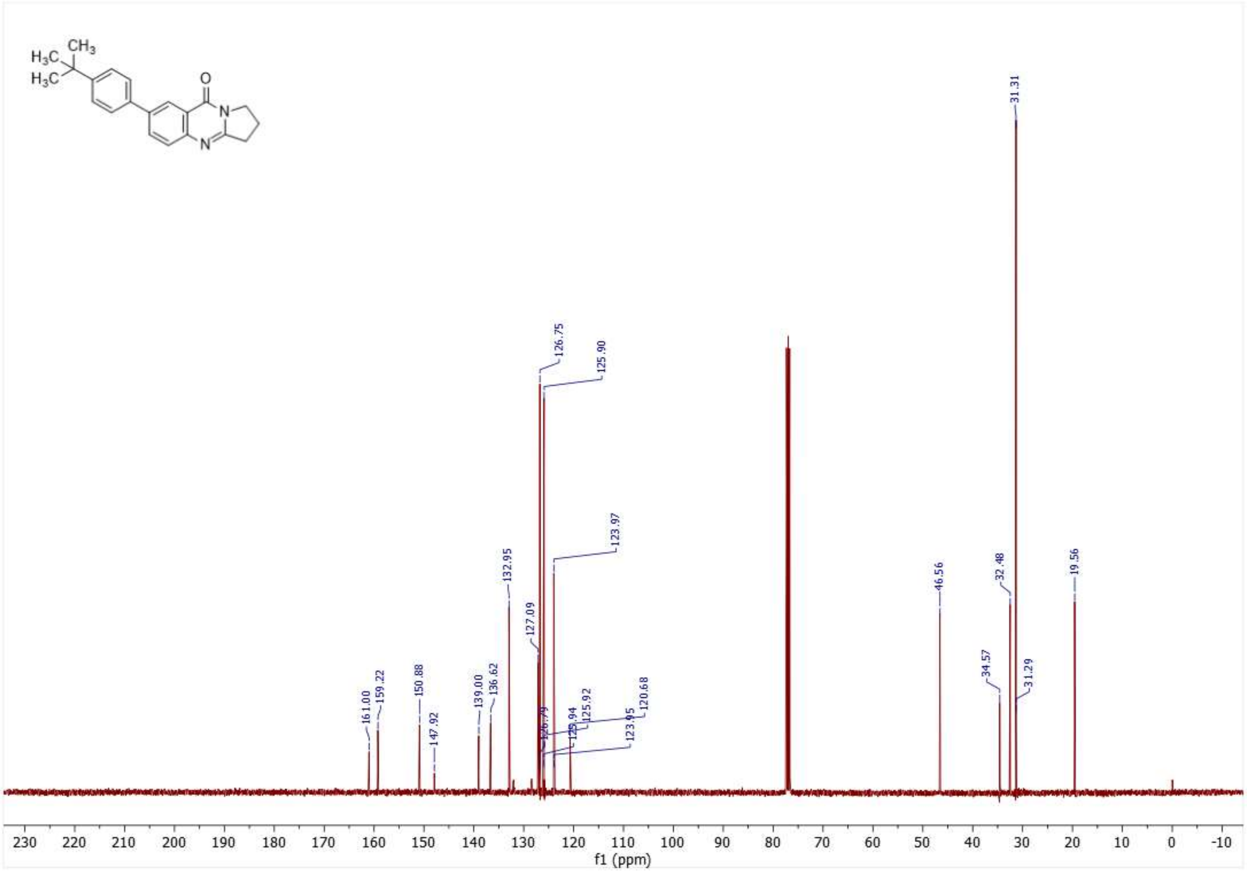

**Figure S14. $^{13}$C NMR spectrum of 3e**

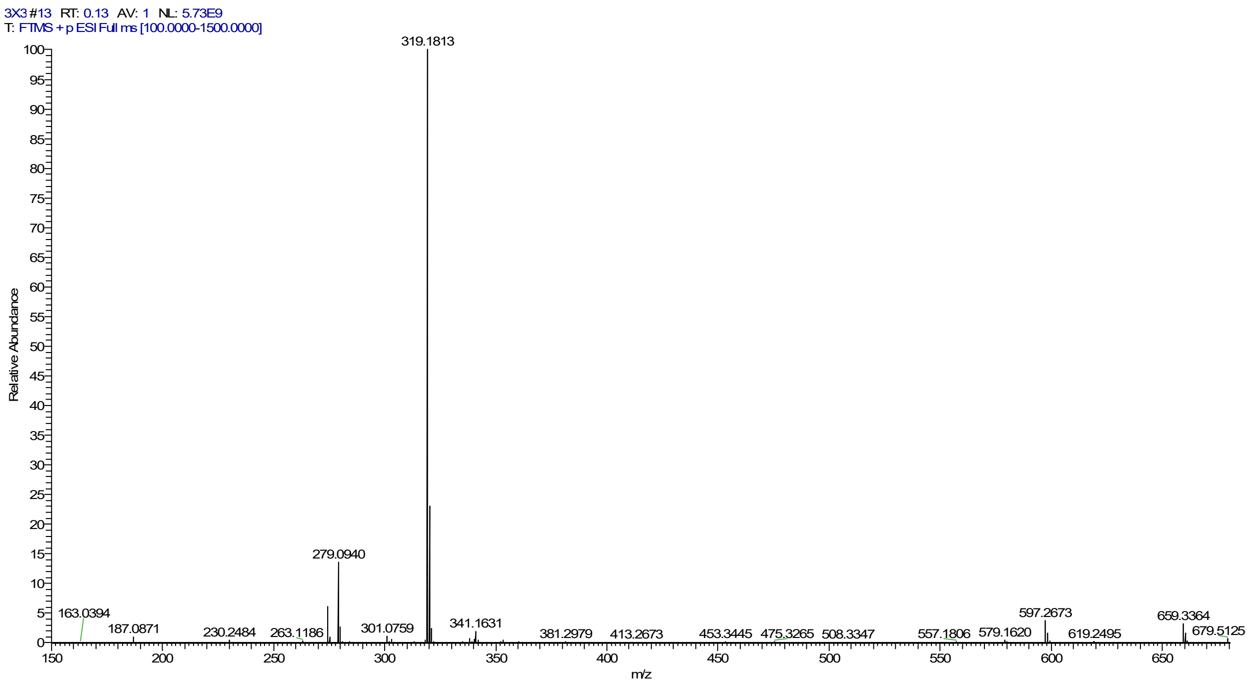


**Figure S15. Mass spectrum of 3e**

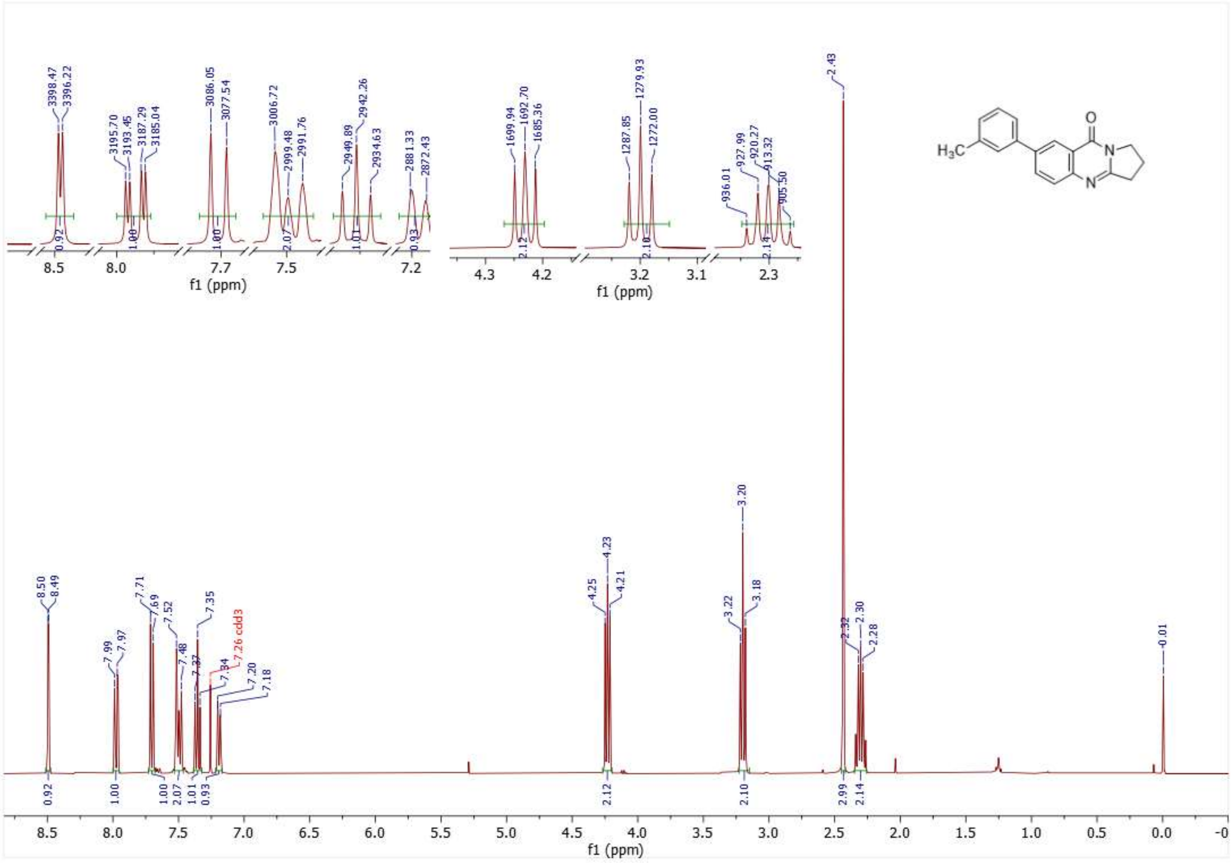


**Figure S16. $^{1}$H NMR spectrum of 3f**

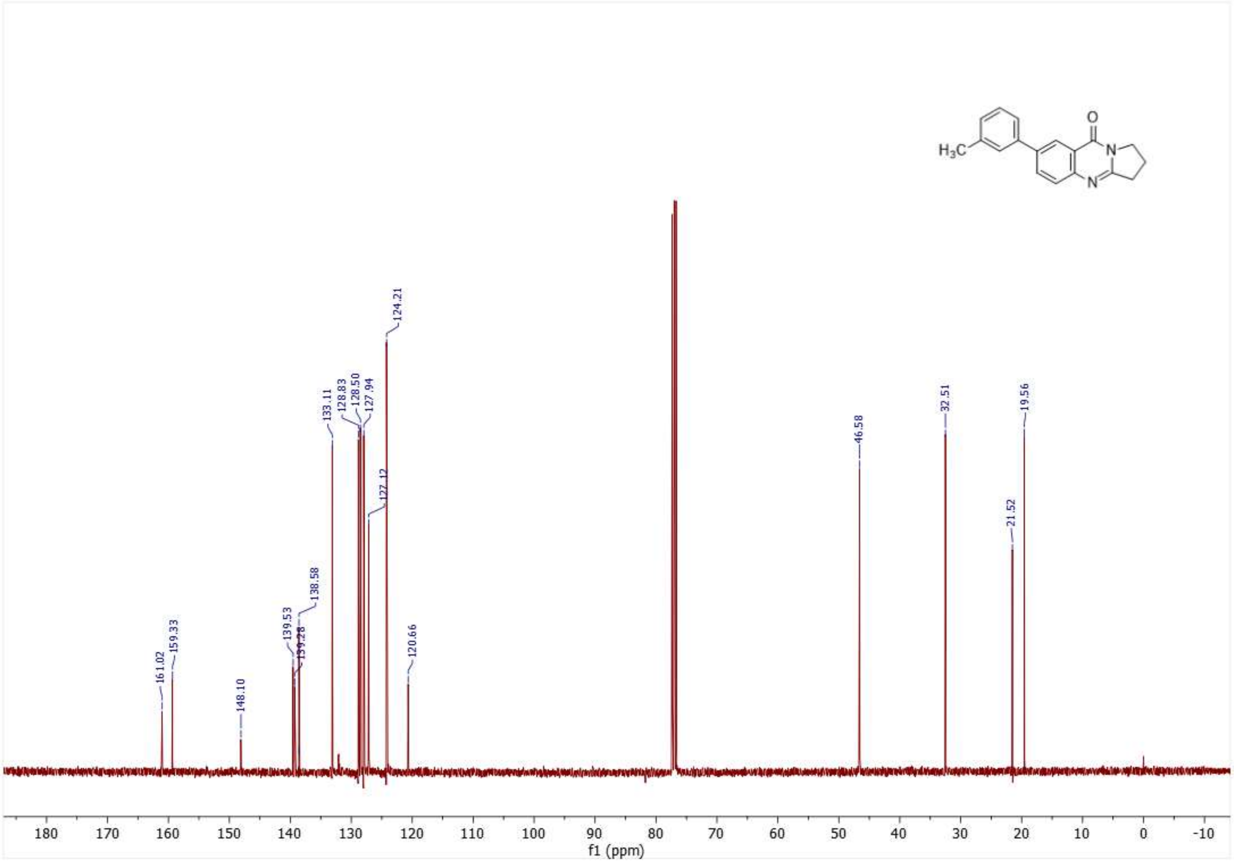


**Figure S17. $^{13}$C NMR spectrum of 3f**

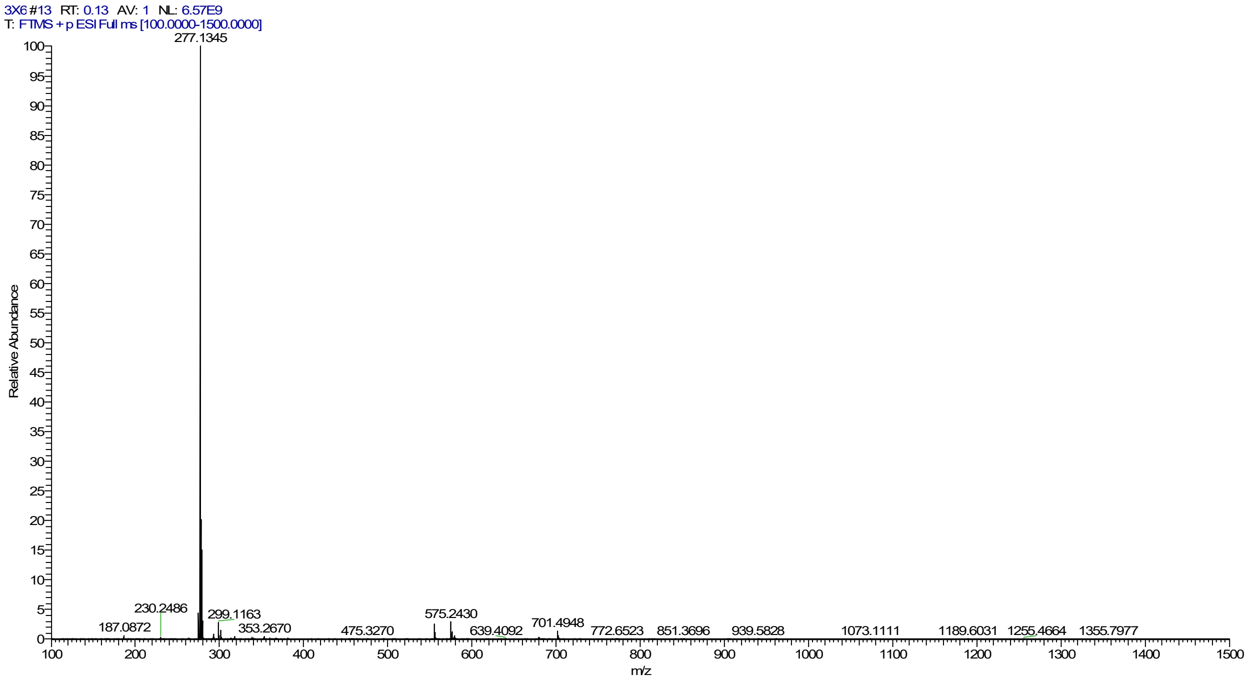


**Figure S18. Mass spectrum of 3f**

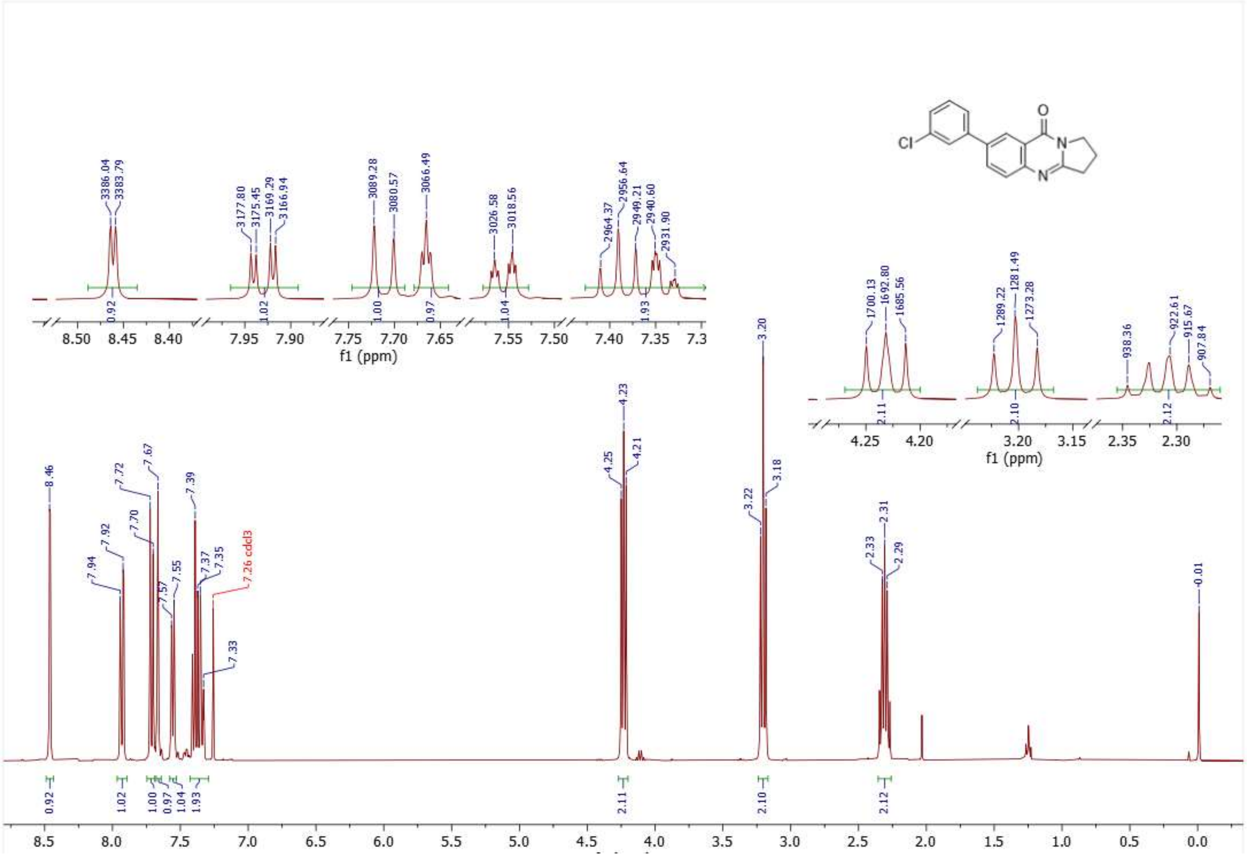


Figure S19. $^{1}H$ NMR spectrum of 3g

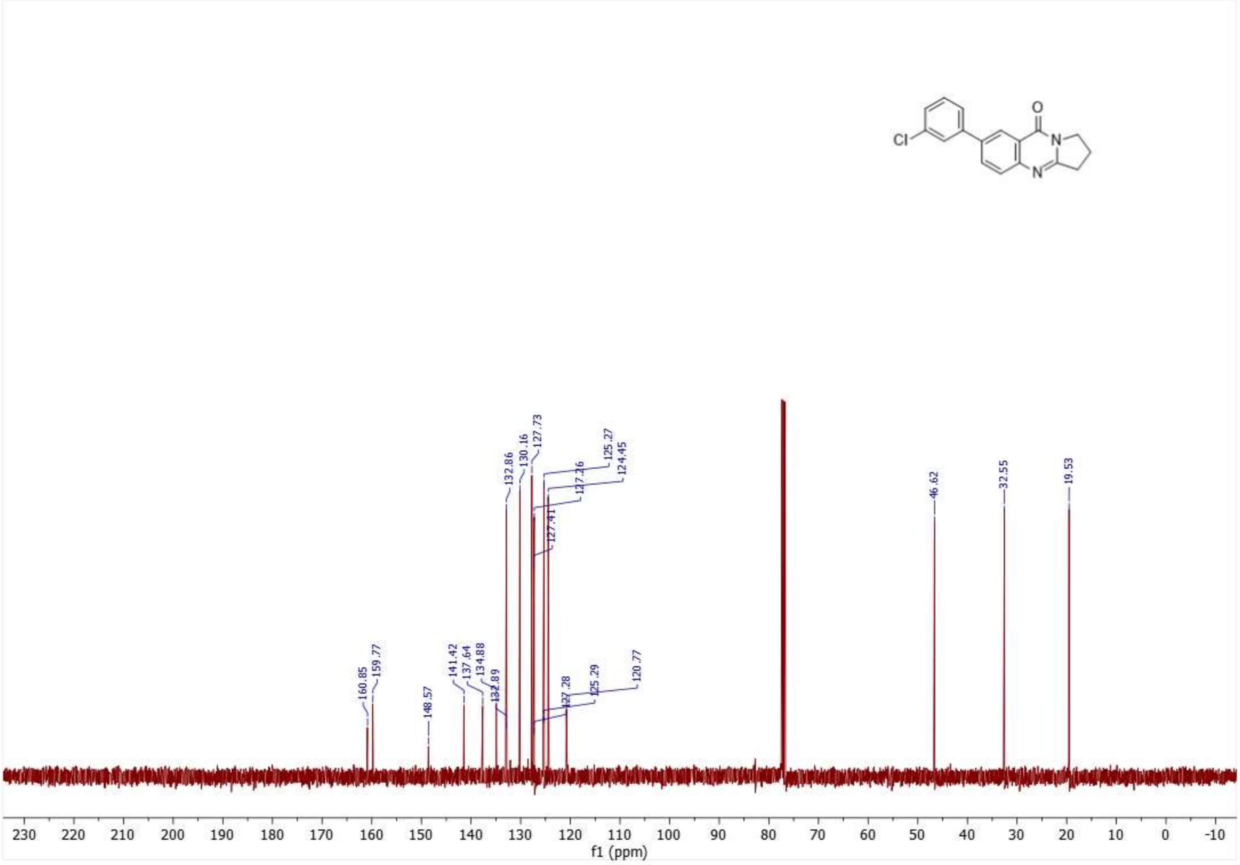


Figure S20. $^{13}C$ NMR spectrum of 3g

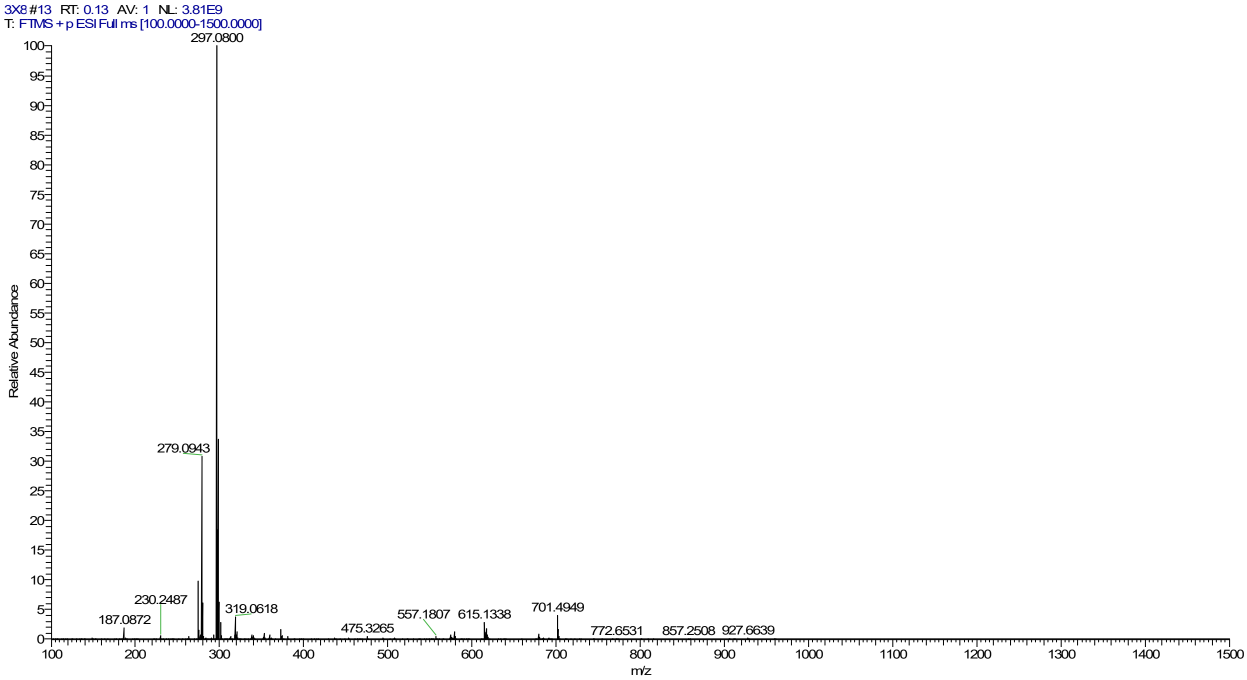


**Figure S21. Mass spectrum of 3g**

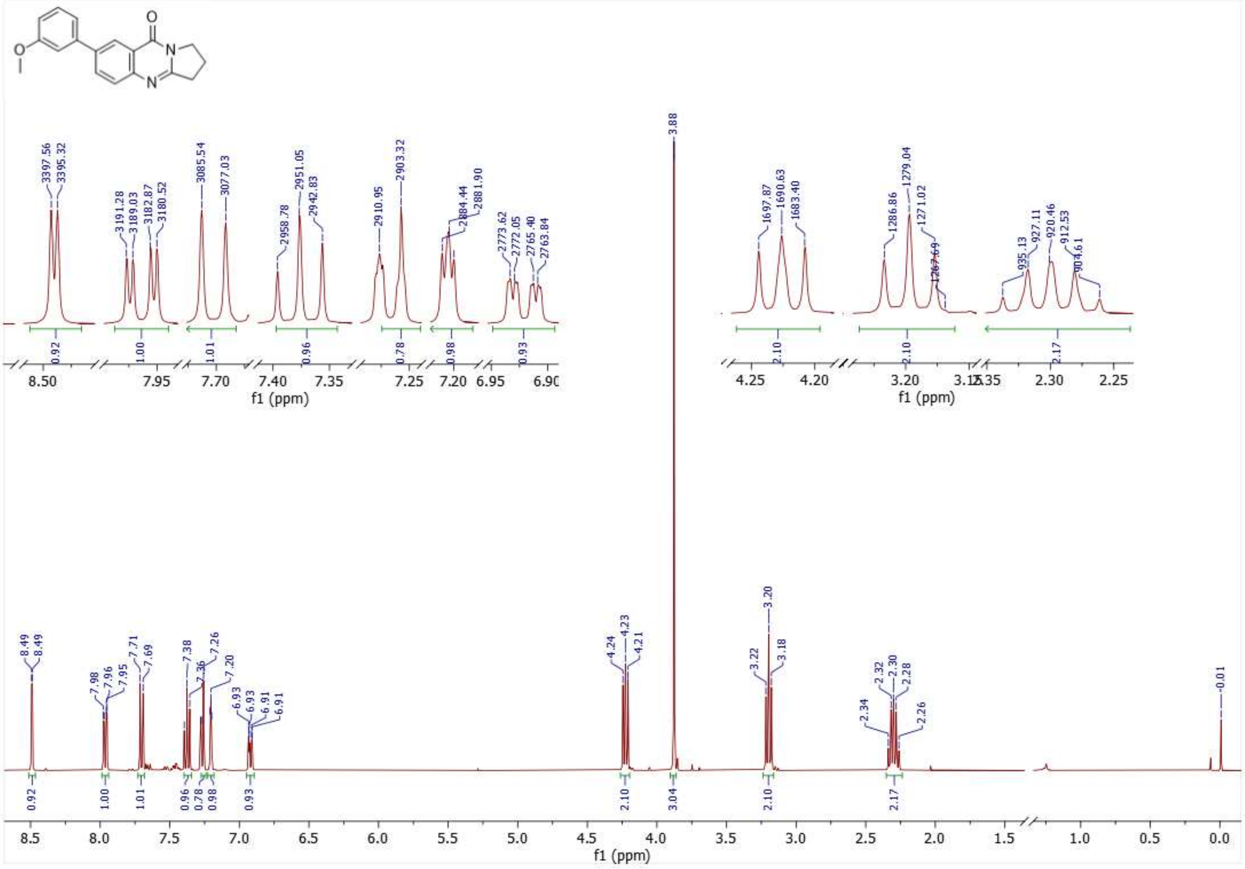


**Figure S22. ¹H NMR spectrum of 3h**

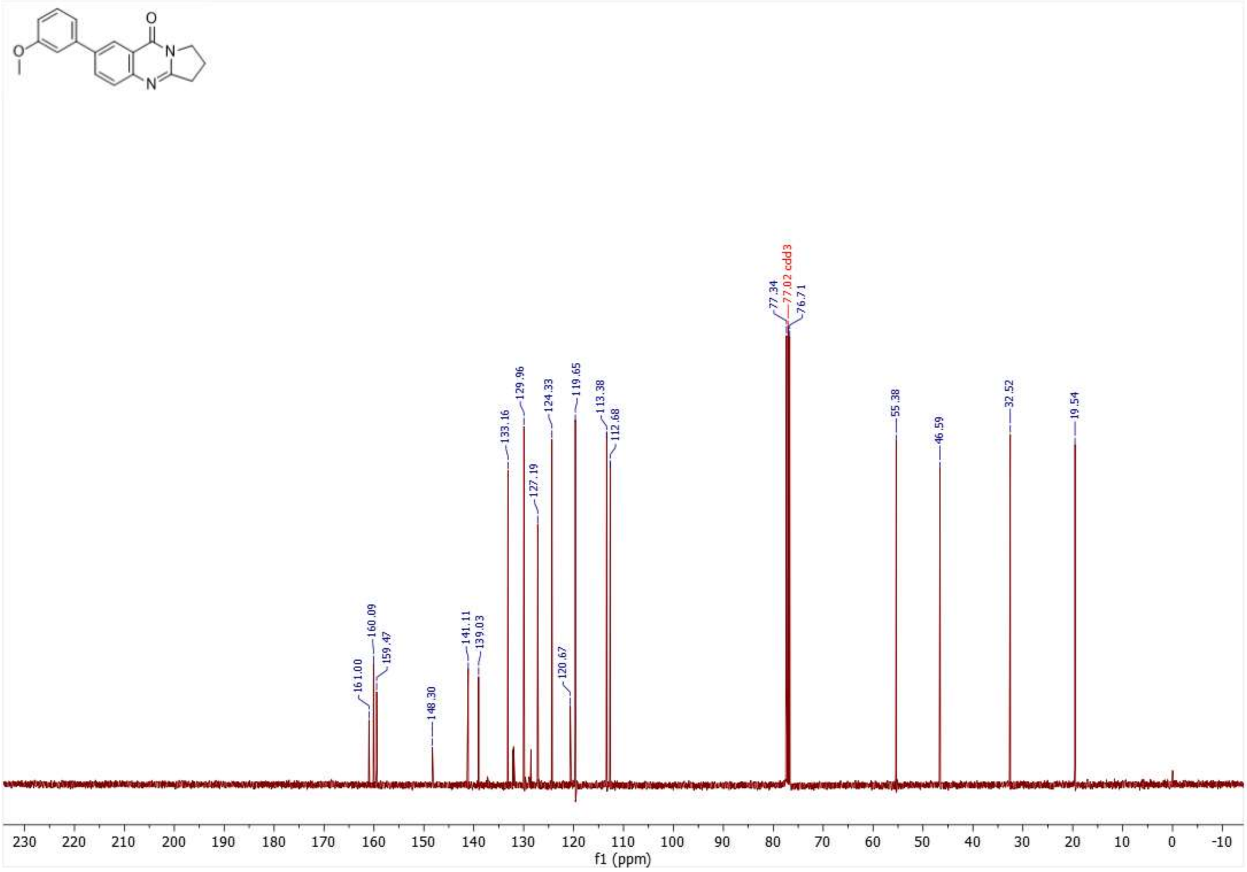


**Figure S23. $^{13}C$ NMR spectrum of 3h**

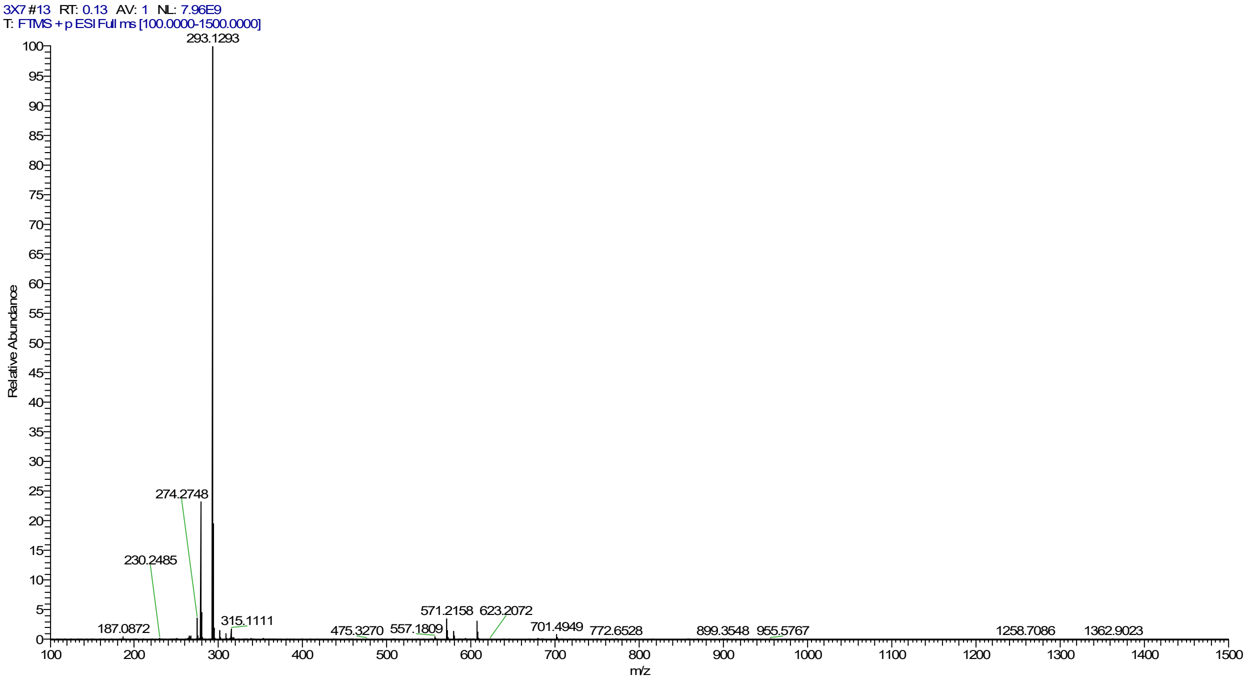


**Figure S24. Mass spectrum of 3h**

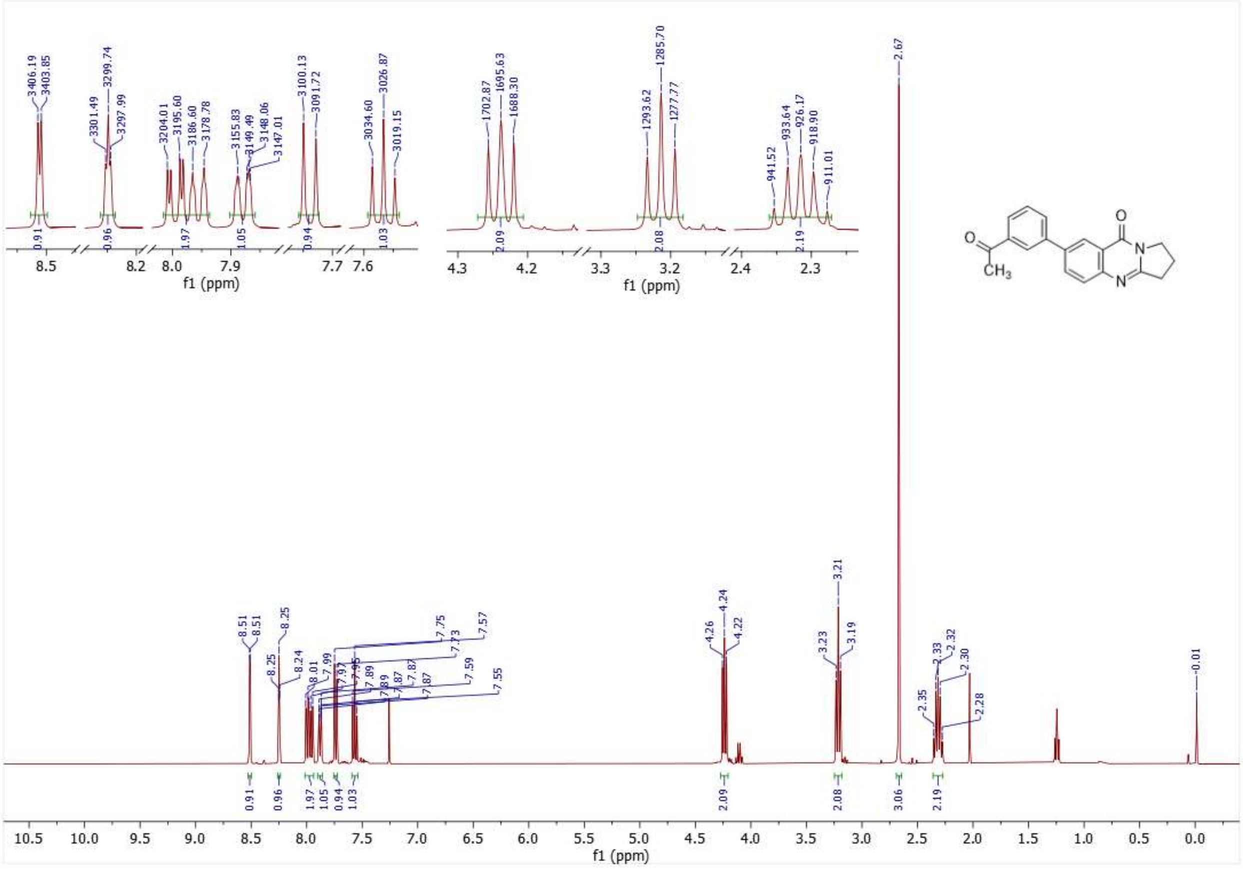

**Figure S25. ¹H NMR spectrum of 3i**

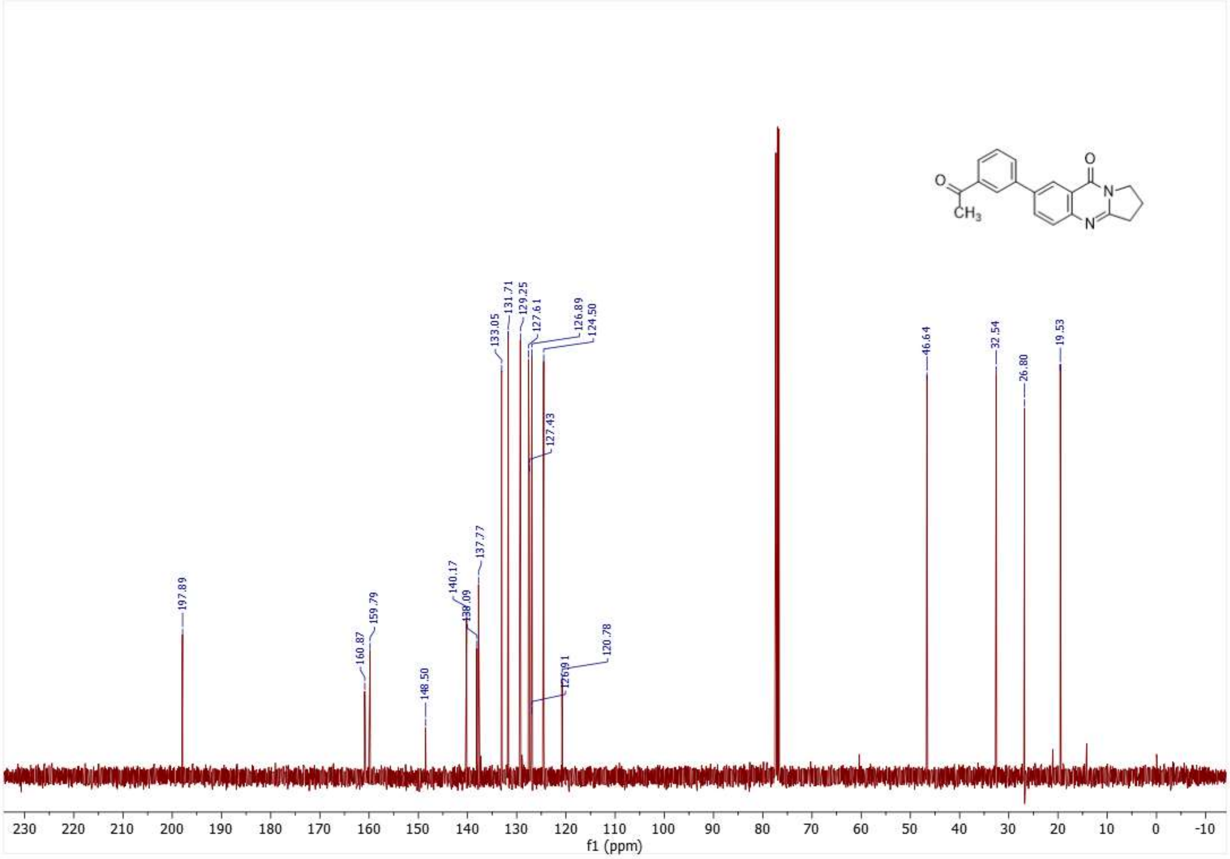

**Figure S26. ¹³C NMR spectrum of 3i**

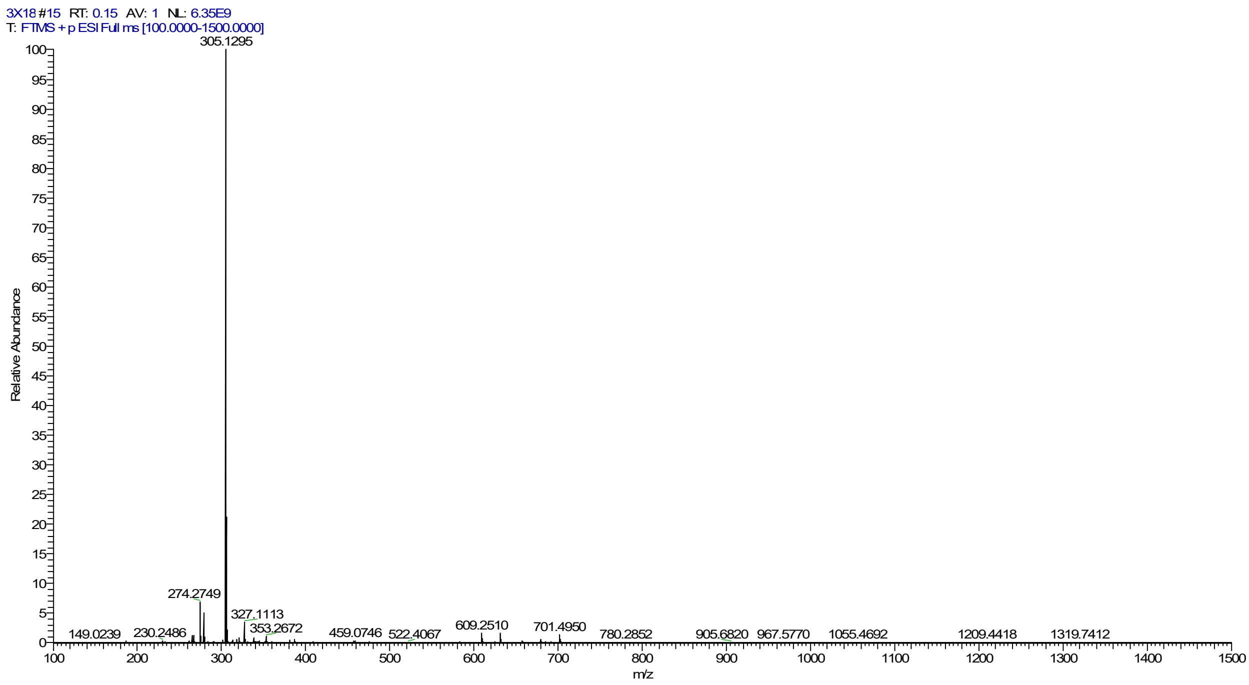


**Figure S27. Mass spectrum of 3i**

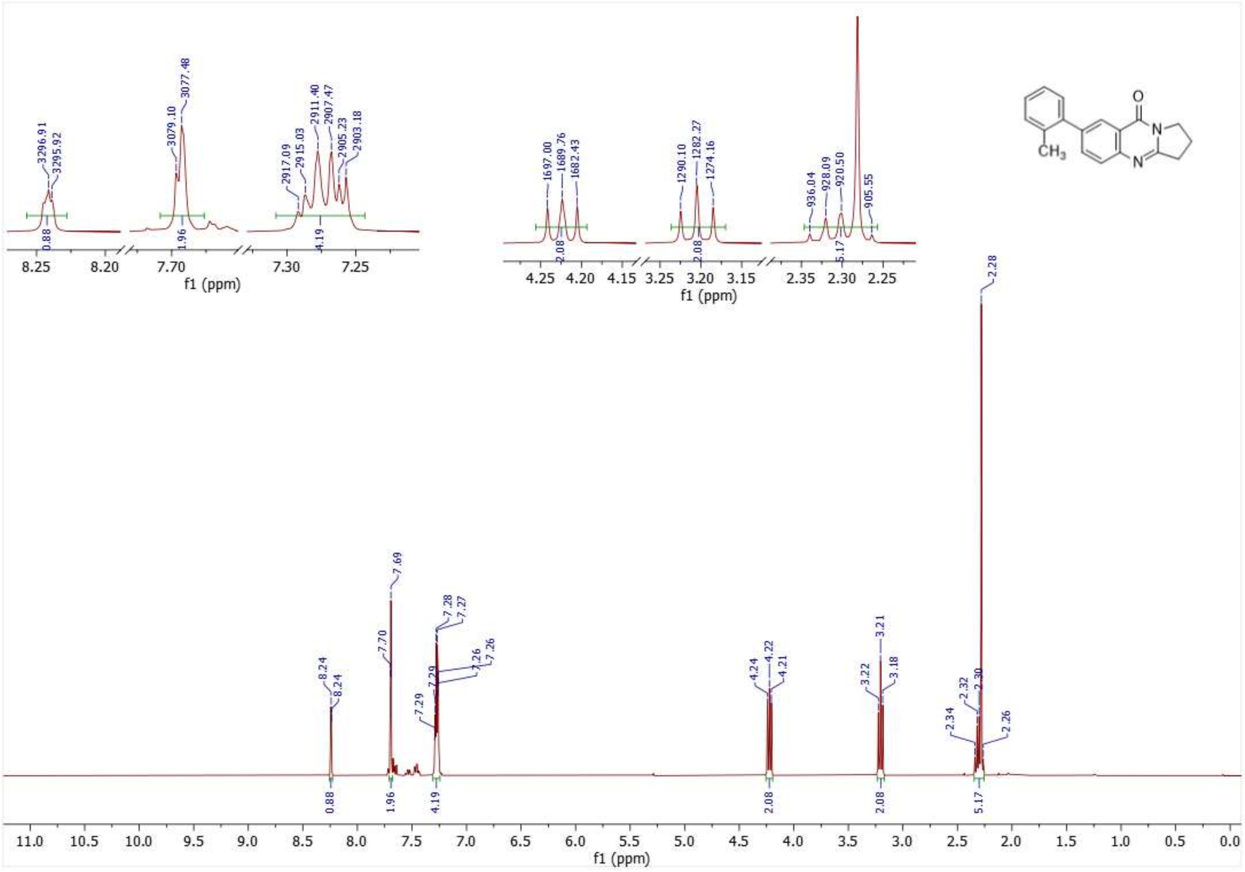


**Figure S28. $^1$H NMR spectrum of 3j**

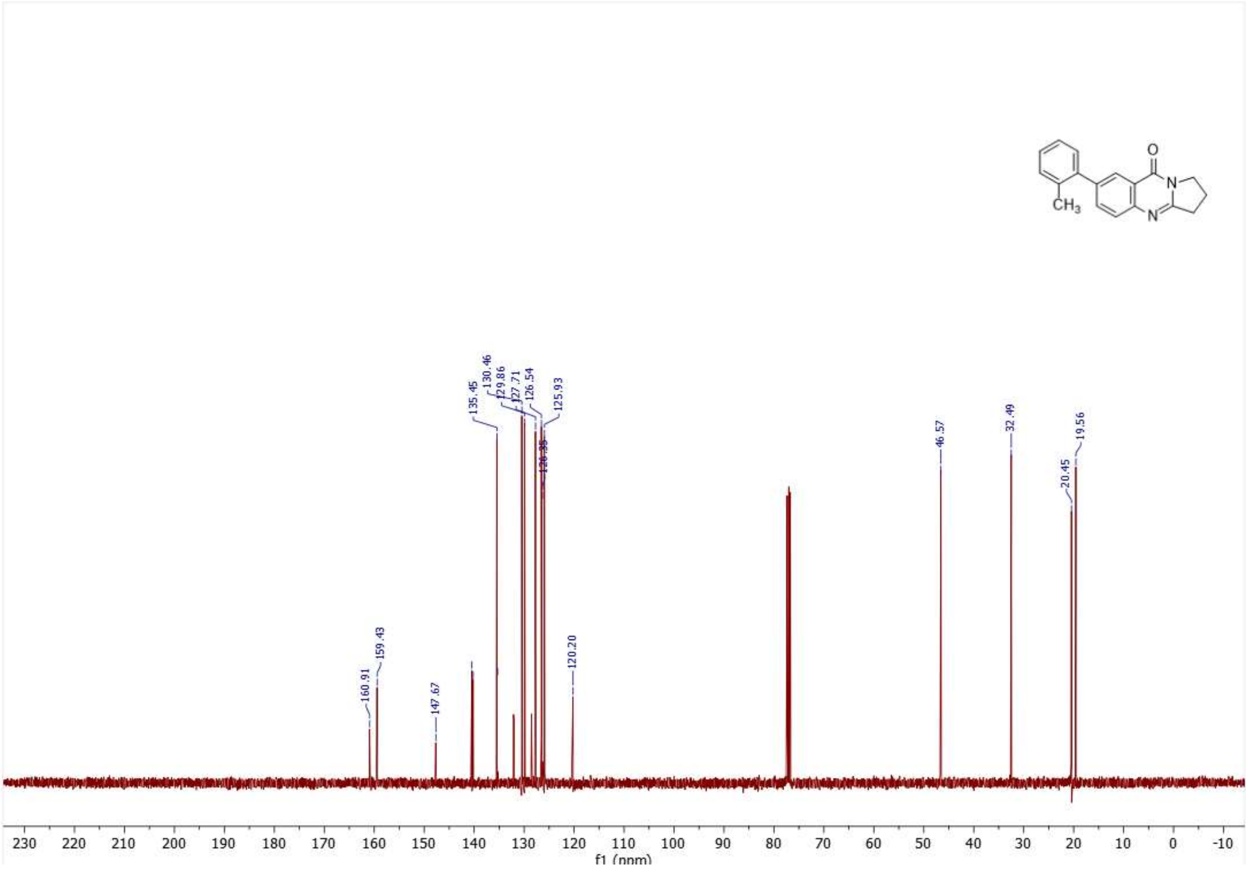


**Figure S29. $^{13}C$ NMR spectrum of 3j**

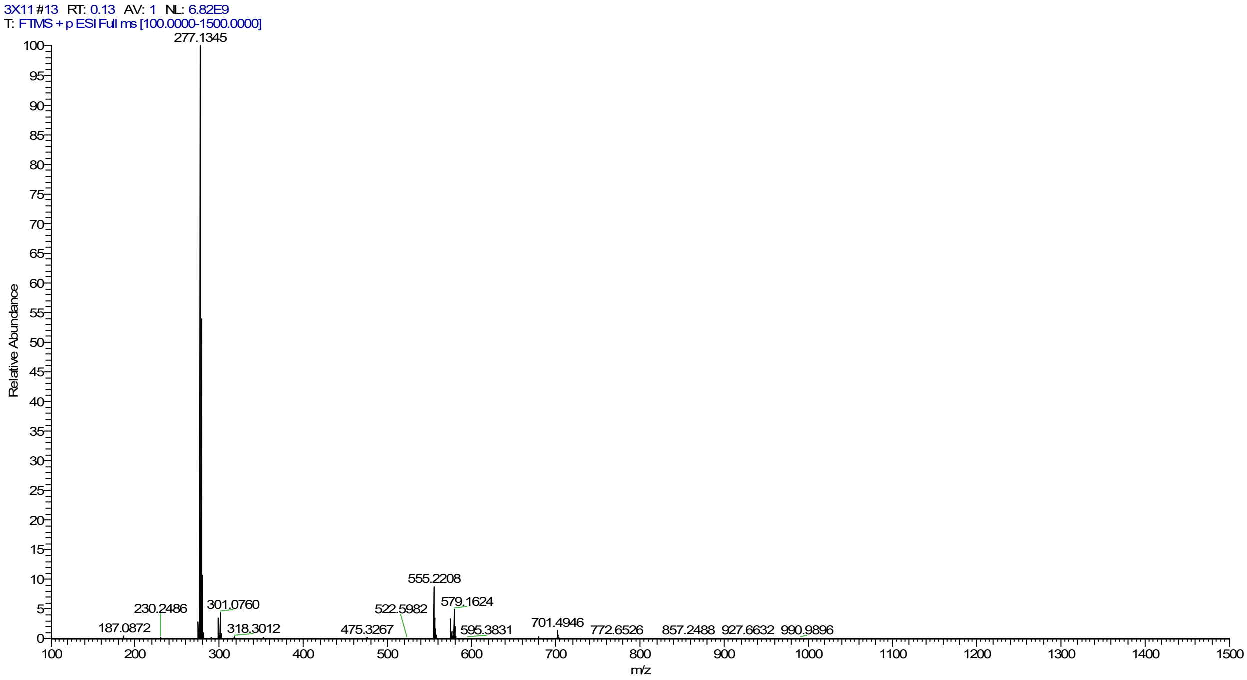


**Figure S30. Mass spectrum of 3j**

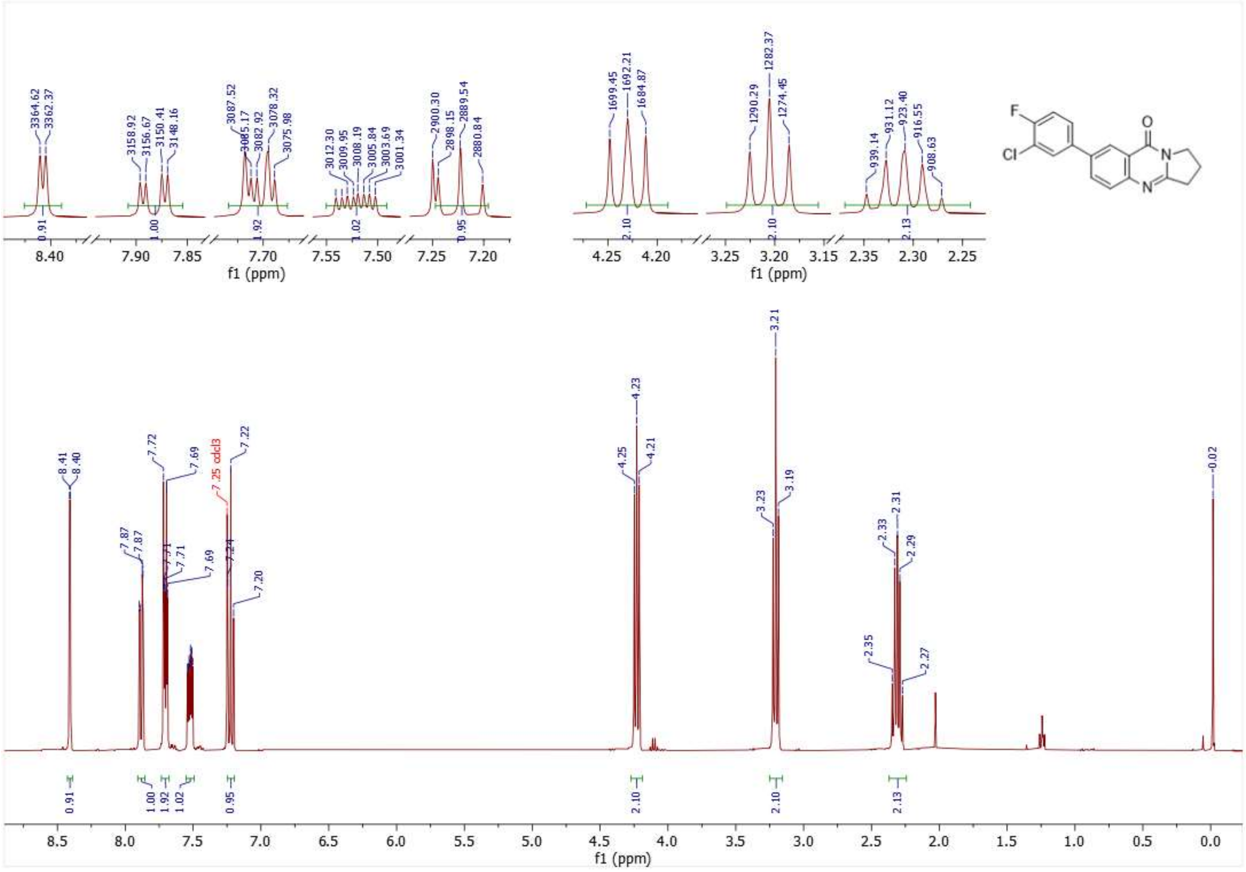

**Figure S31. $^{1}$H NMR spectrum of 3k**

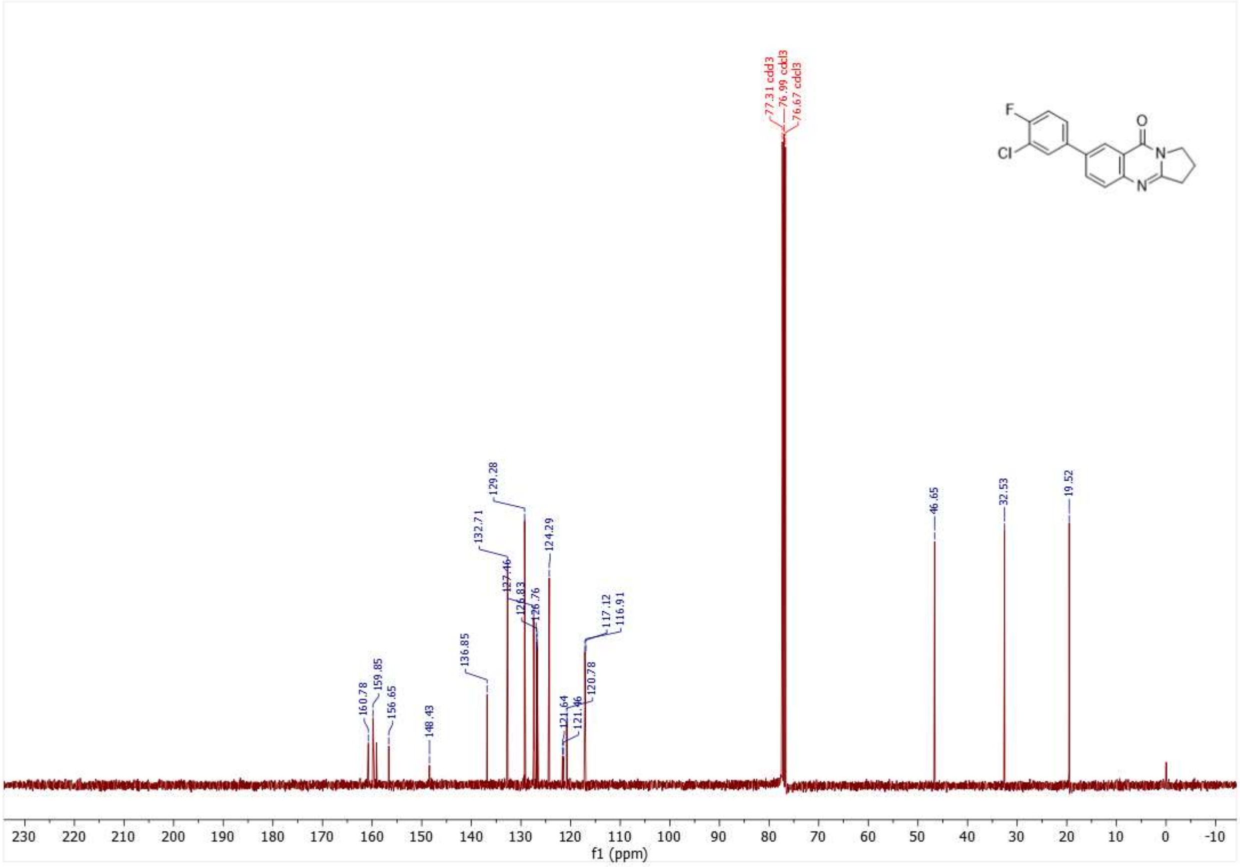

**Figure S32. $^{13}$C NMR spectrum of 3k**

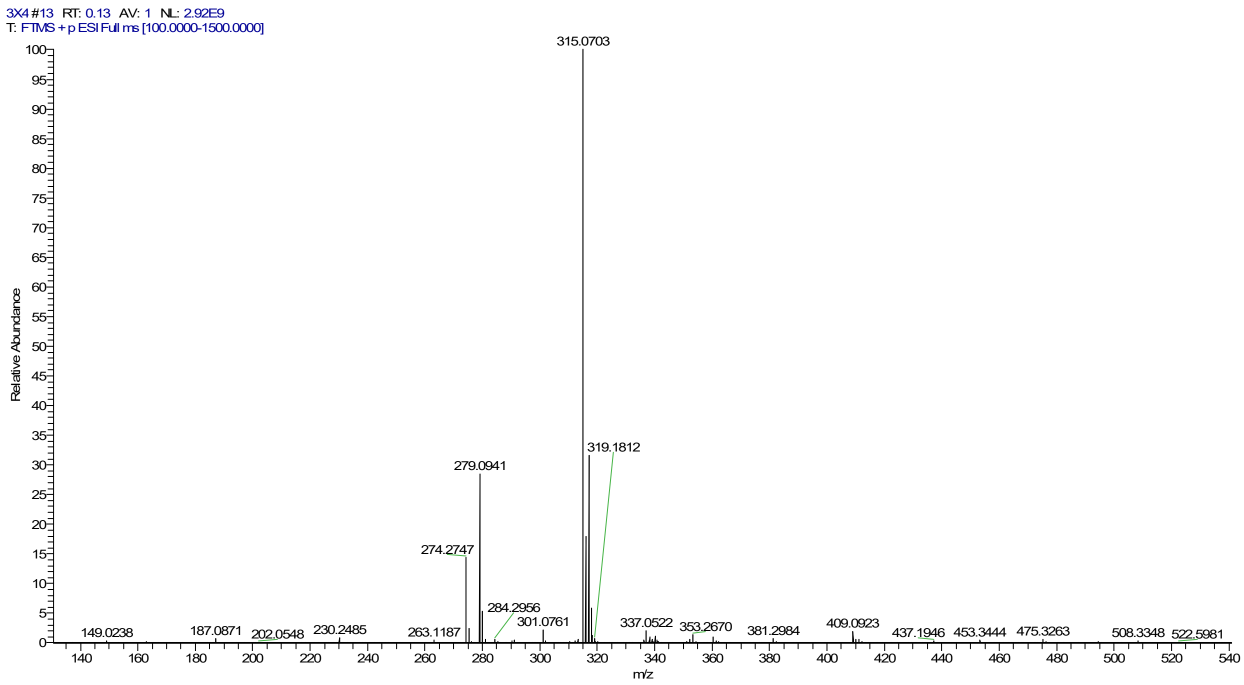

**Figure S33. Mass spectrum of 3k**

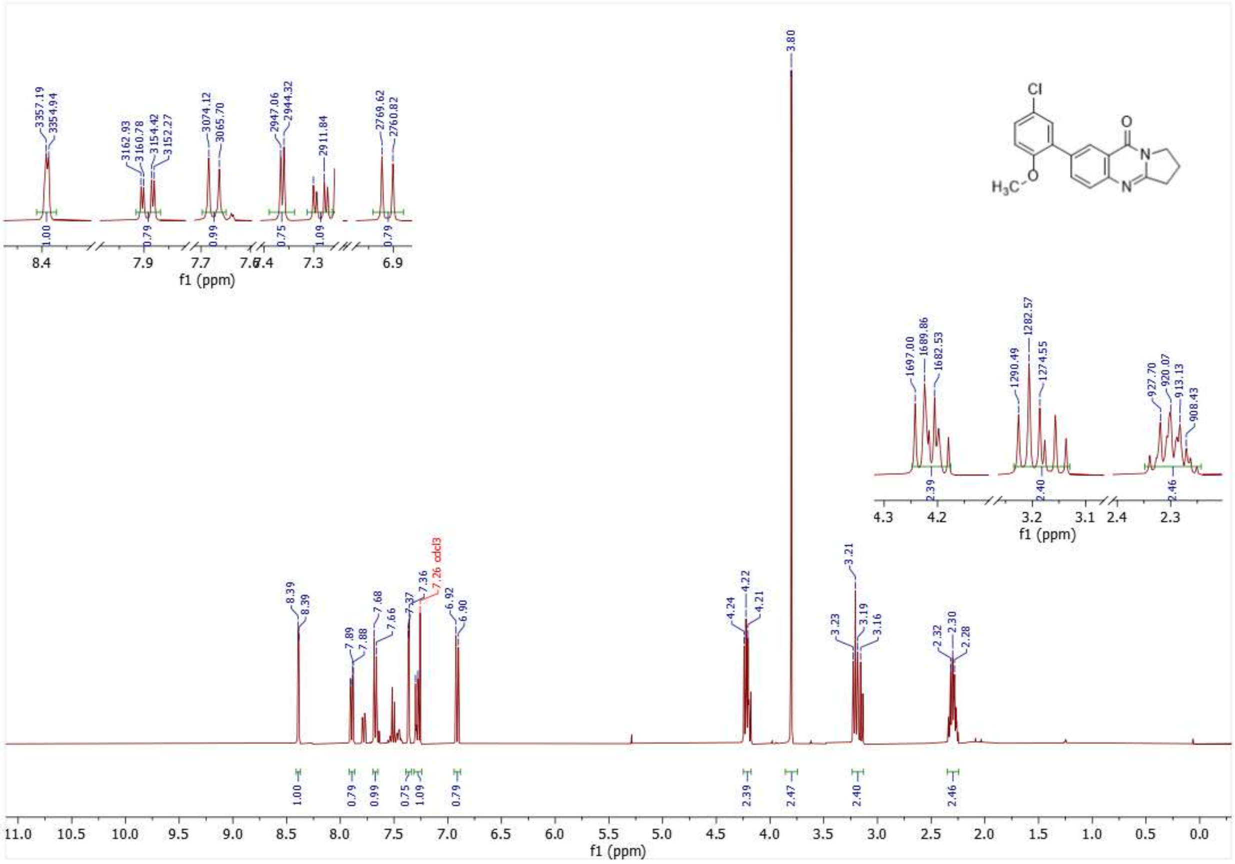

**Figure S34. $^{1}$H NMR spectrum of 3l**

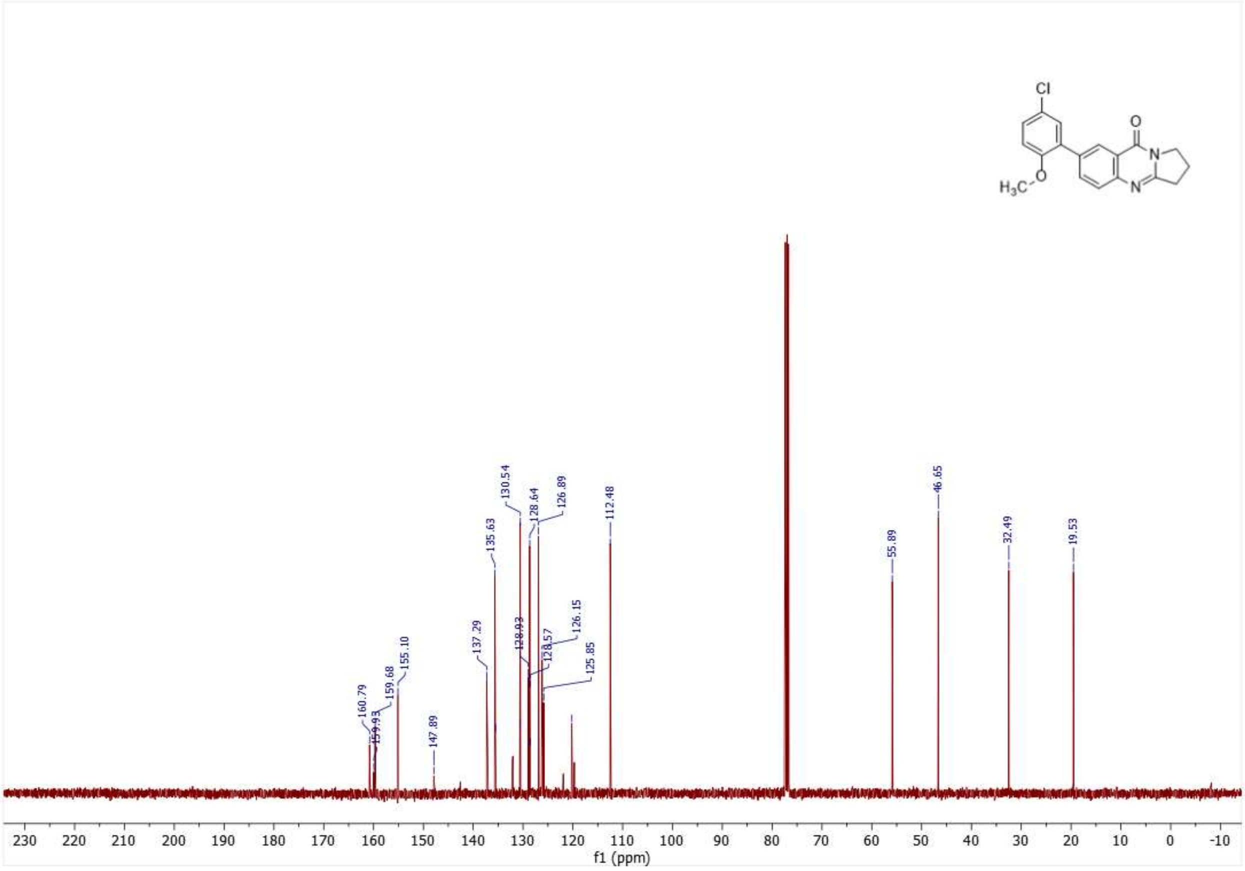


**Figure S35. $^{13}$C NMR spectrum of 3l**

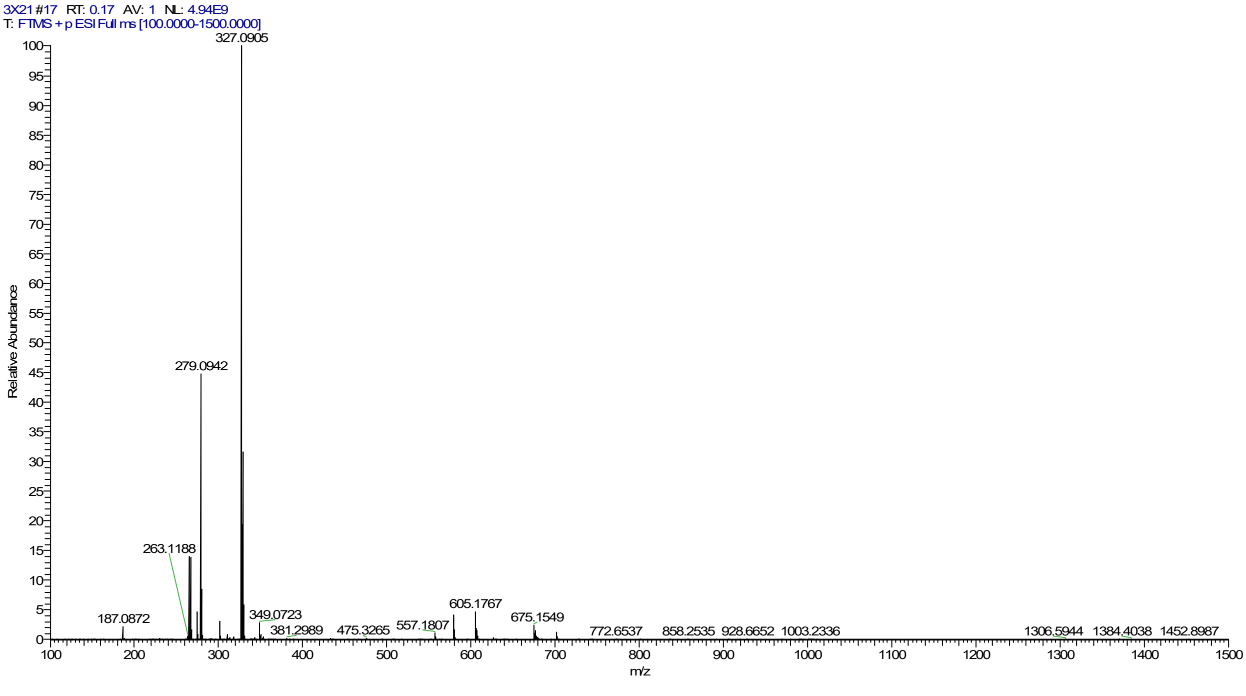


**Figure S36. Mass spectrum of 3l**

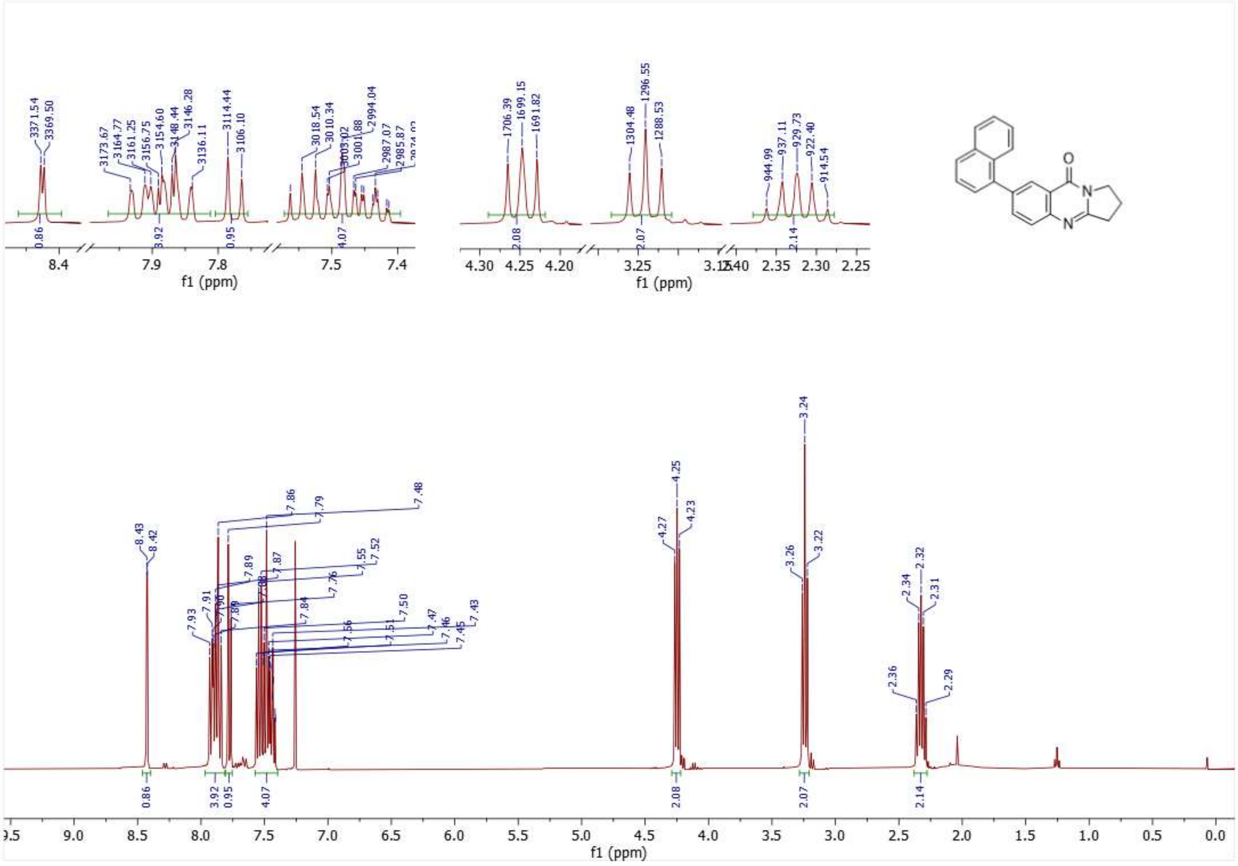


**Figure S37. $^{1}$H NMR spectrum of 3m**

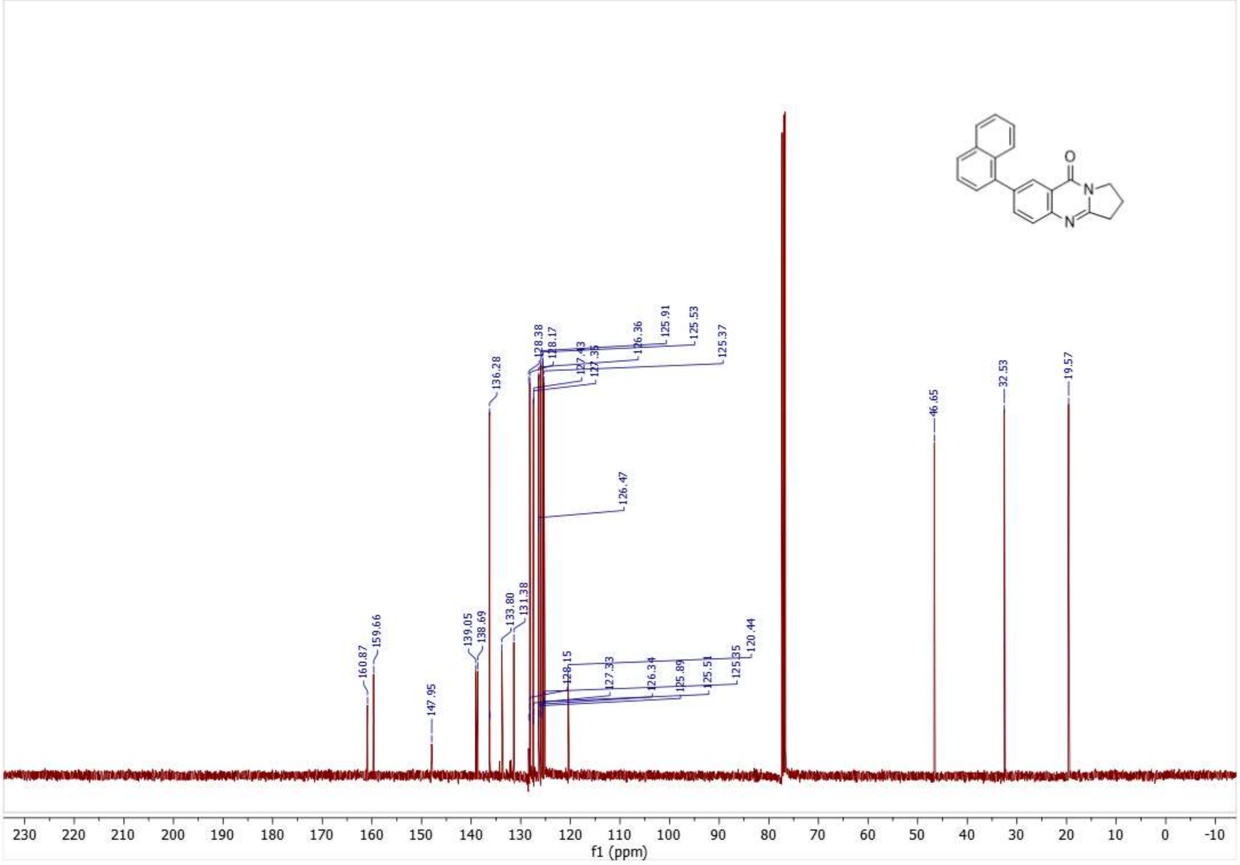


**Figure S38. $^{13}$C NMR spectrum of 3m**

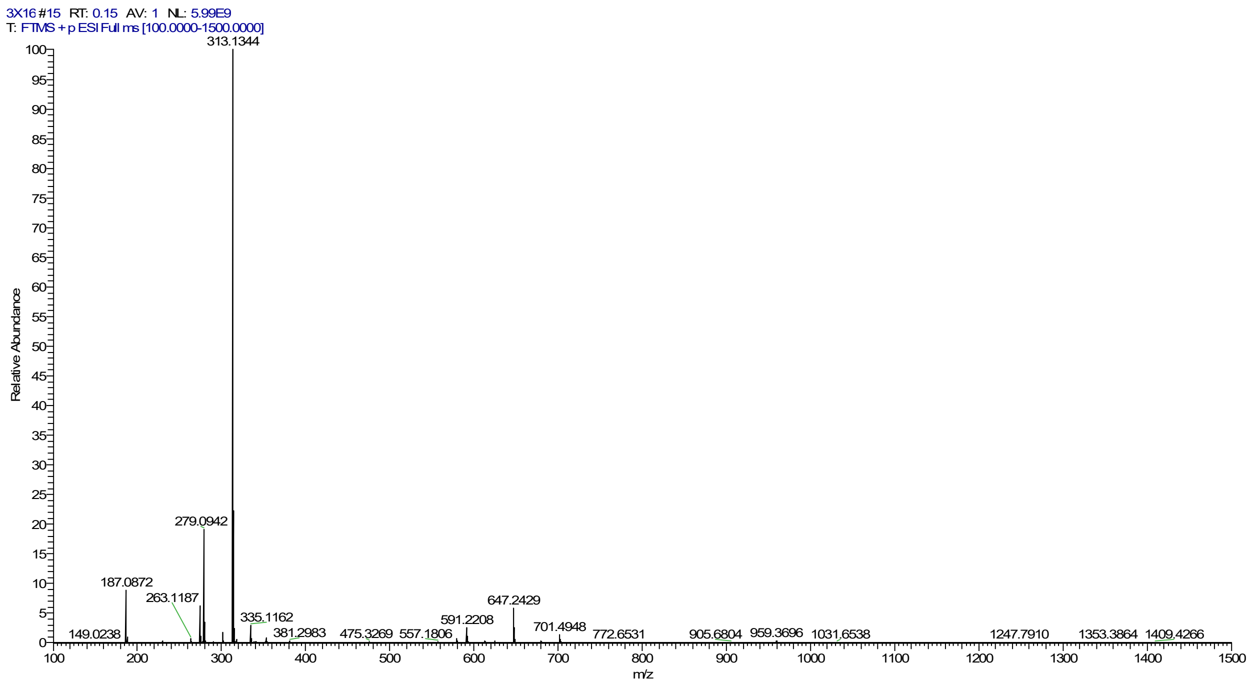


**Figure S39. Mass spectrum of 3m**

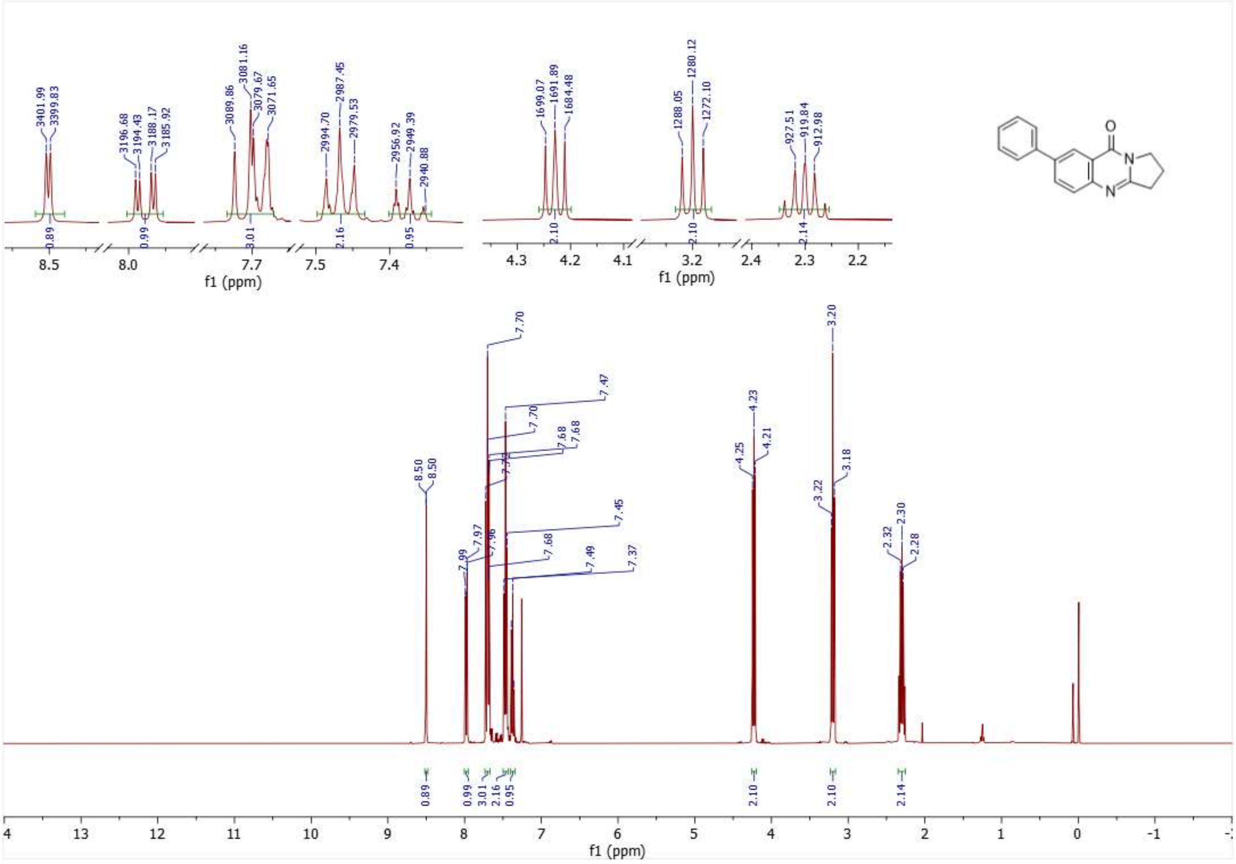


**Figure S40. $^{1}$H NMR spectrum of 3n**

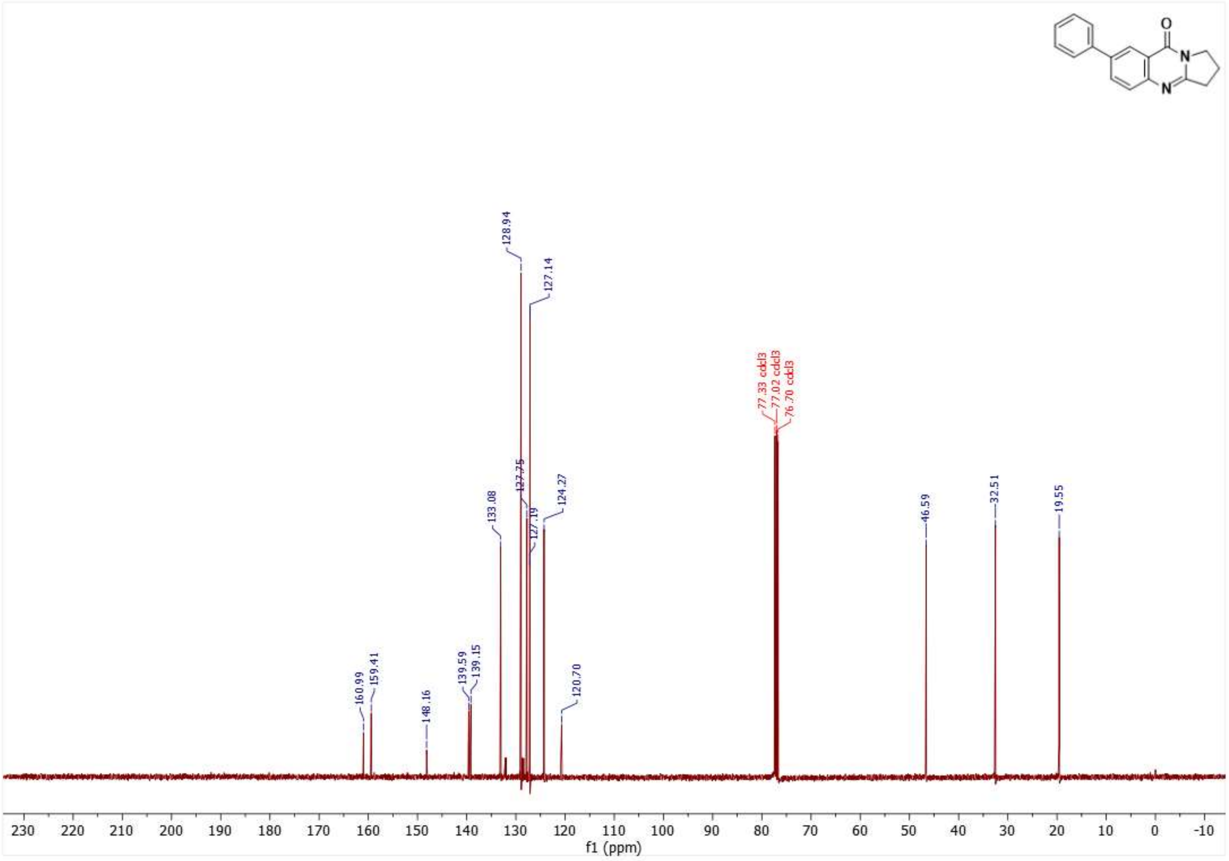


**Figure S41. $^{13}$C NMR spectrum of 3n**

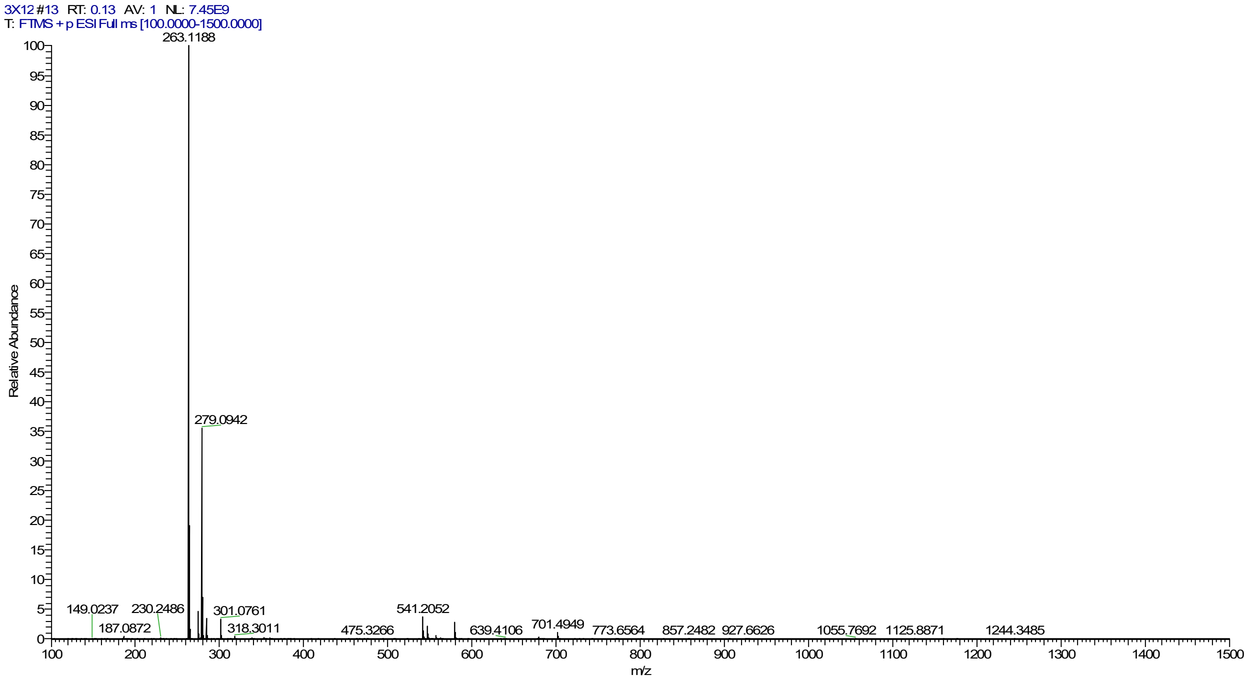


**Figure S42. Mass spectrum of 3n**

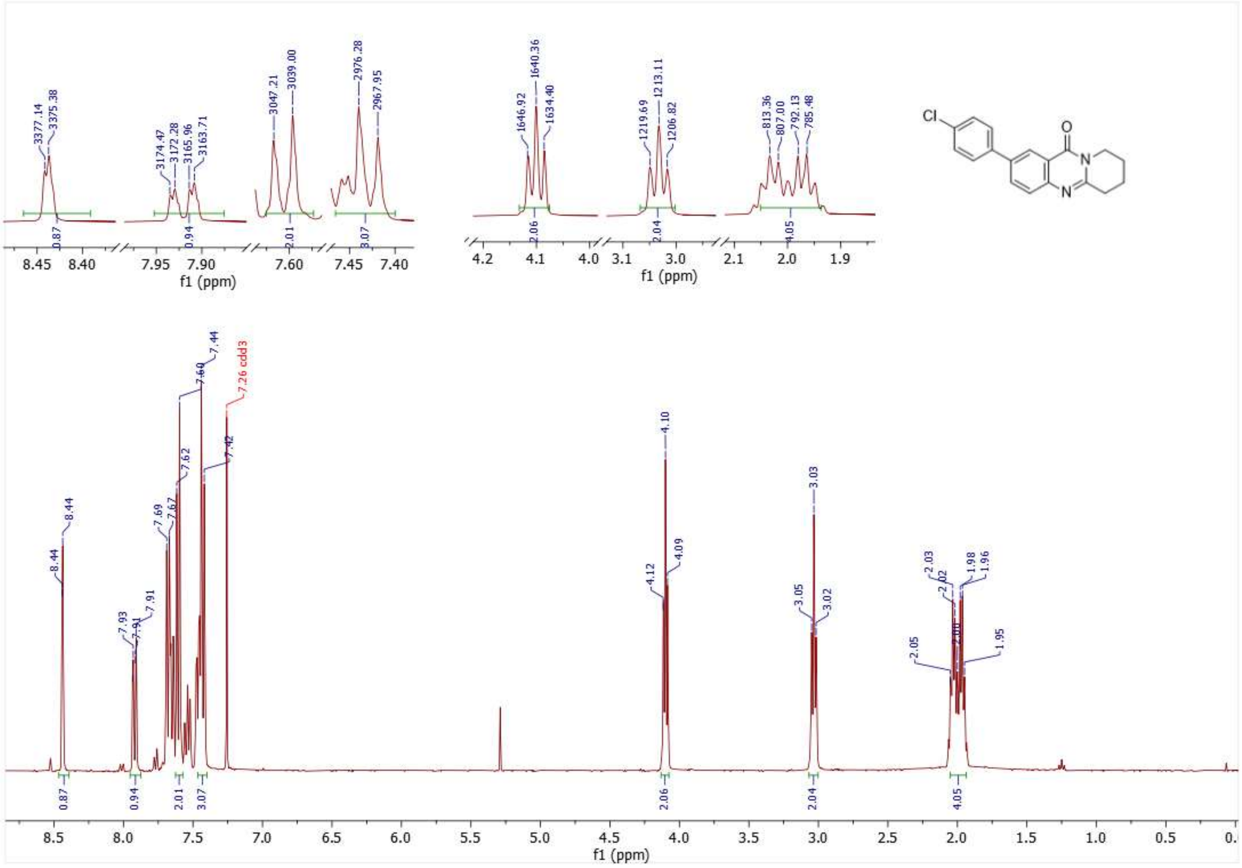

**Figure S43. ¹H NMR spectrum of 4a**

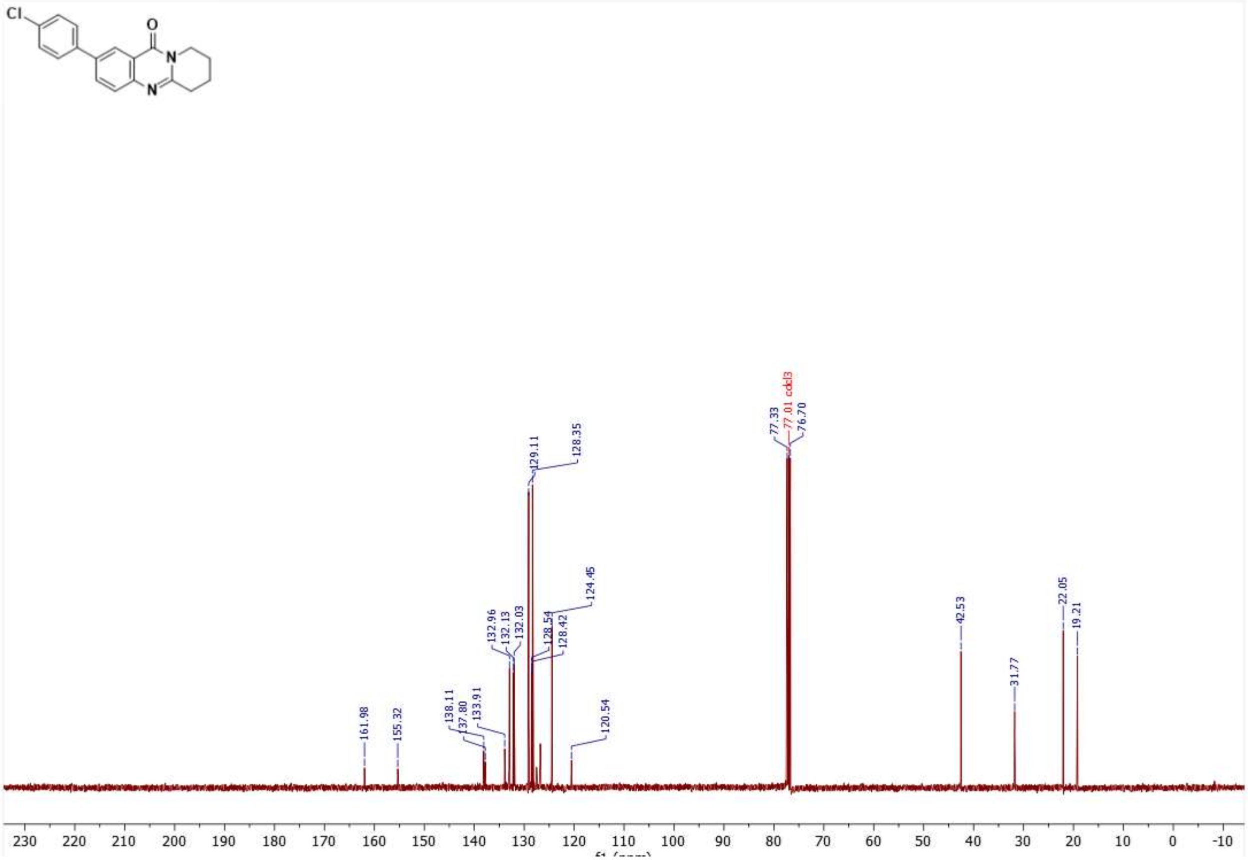

**Figure S44. ¹³C NMR spectrum of 4a**

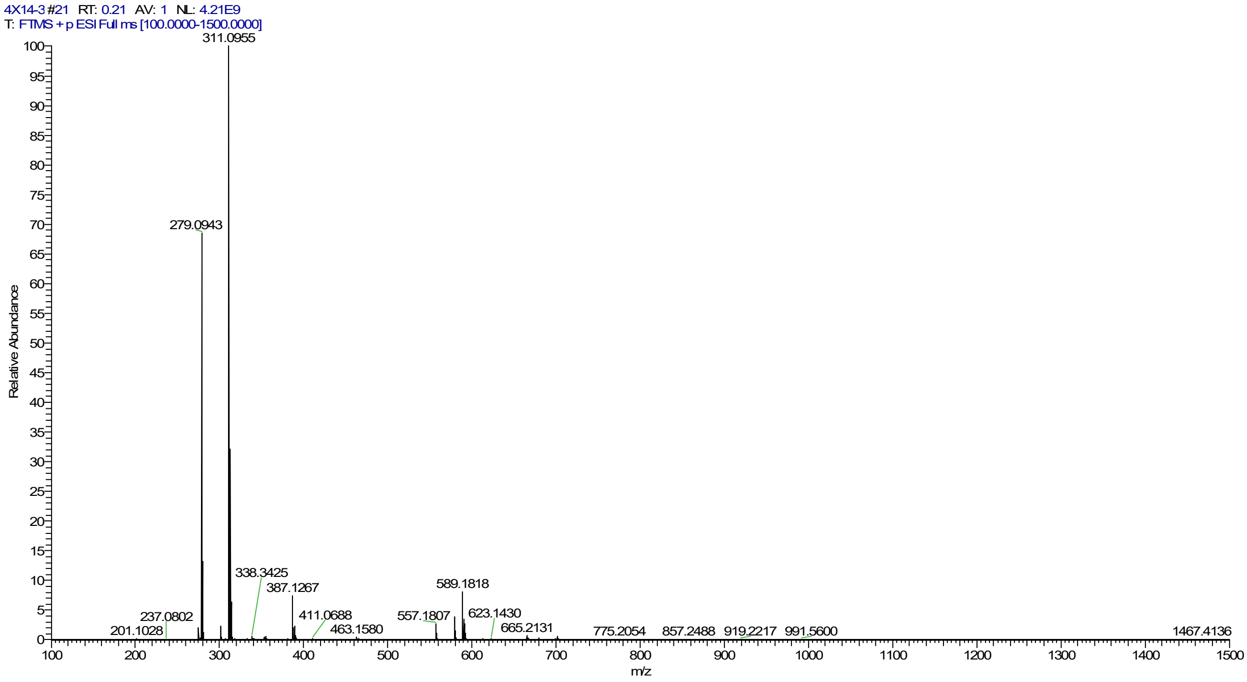


**Figure S45. Mass spectrum of 4a**

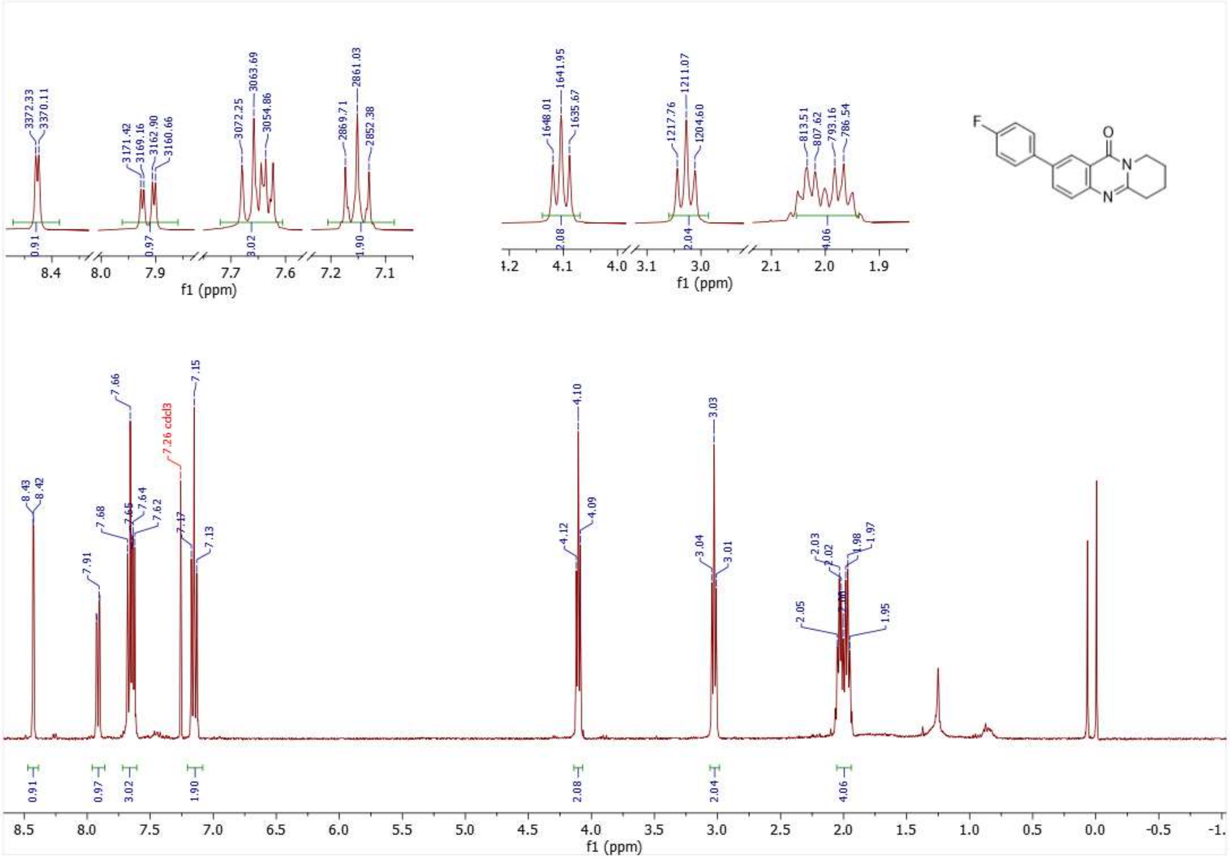


**Figure S46. ¹H NMR spectrum of 4b**

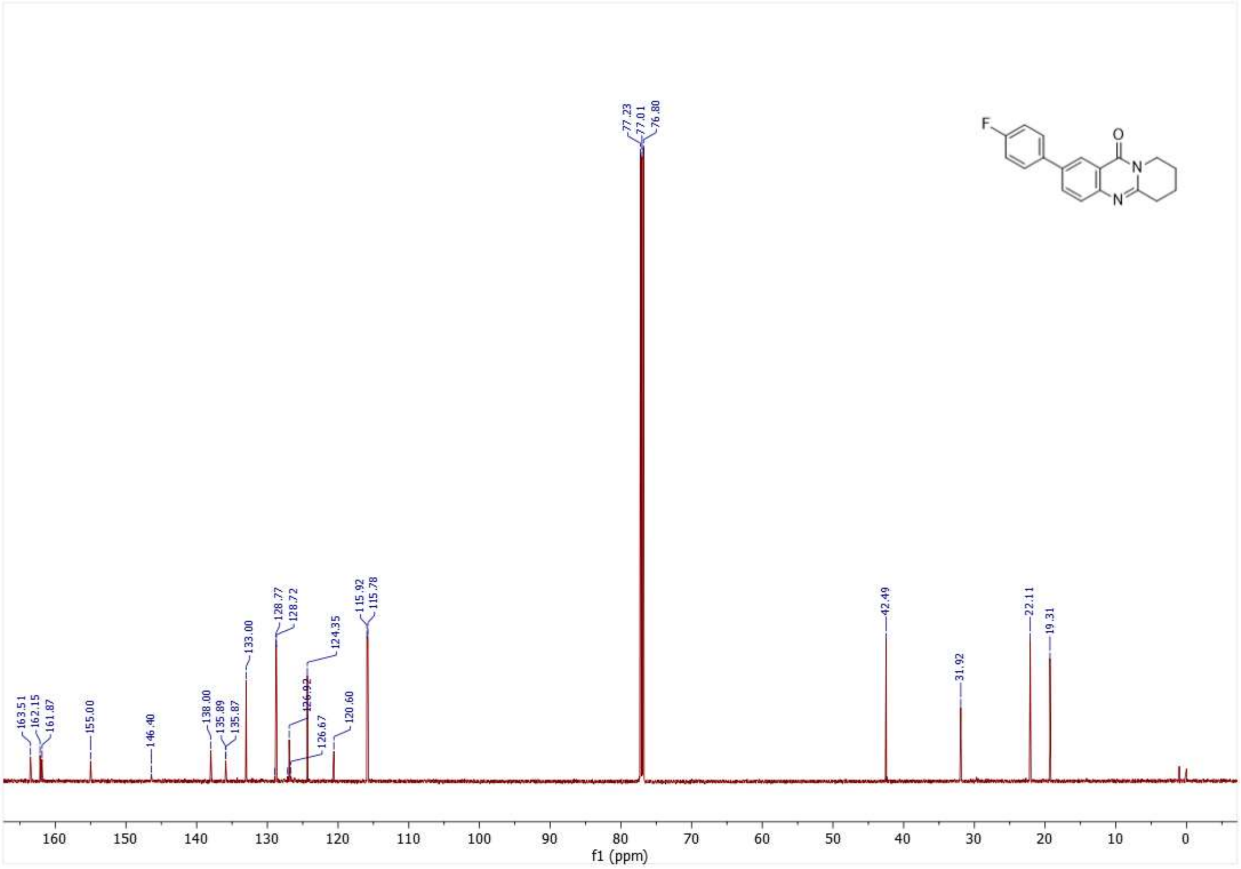


**Figure S47.** $^{13}$**C NMR spectrum of 4b**

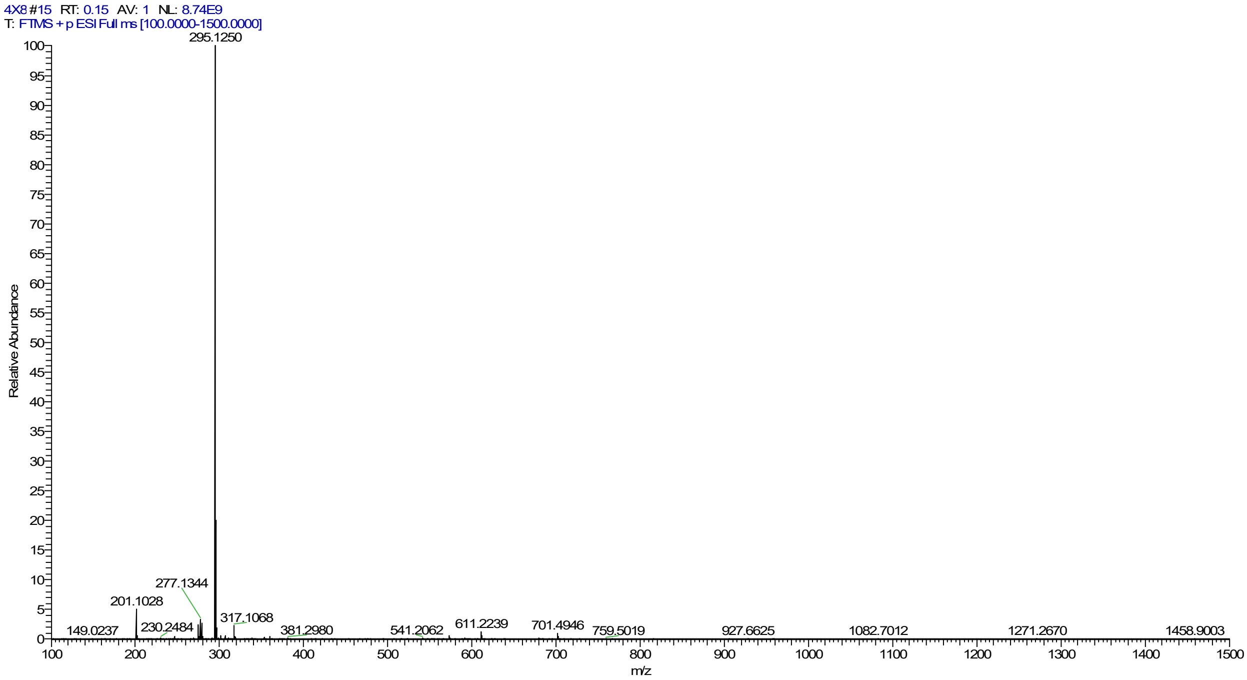


**Figure S48. Mass spectrum of 4b**

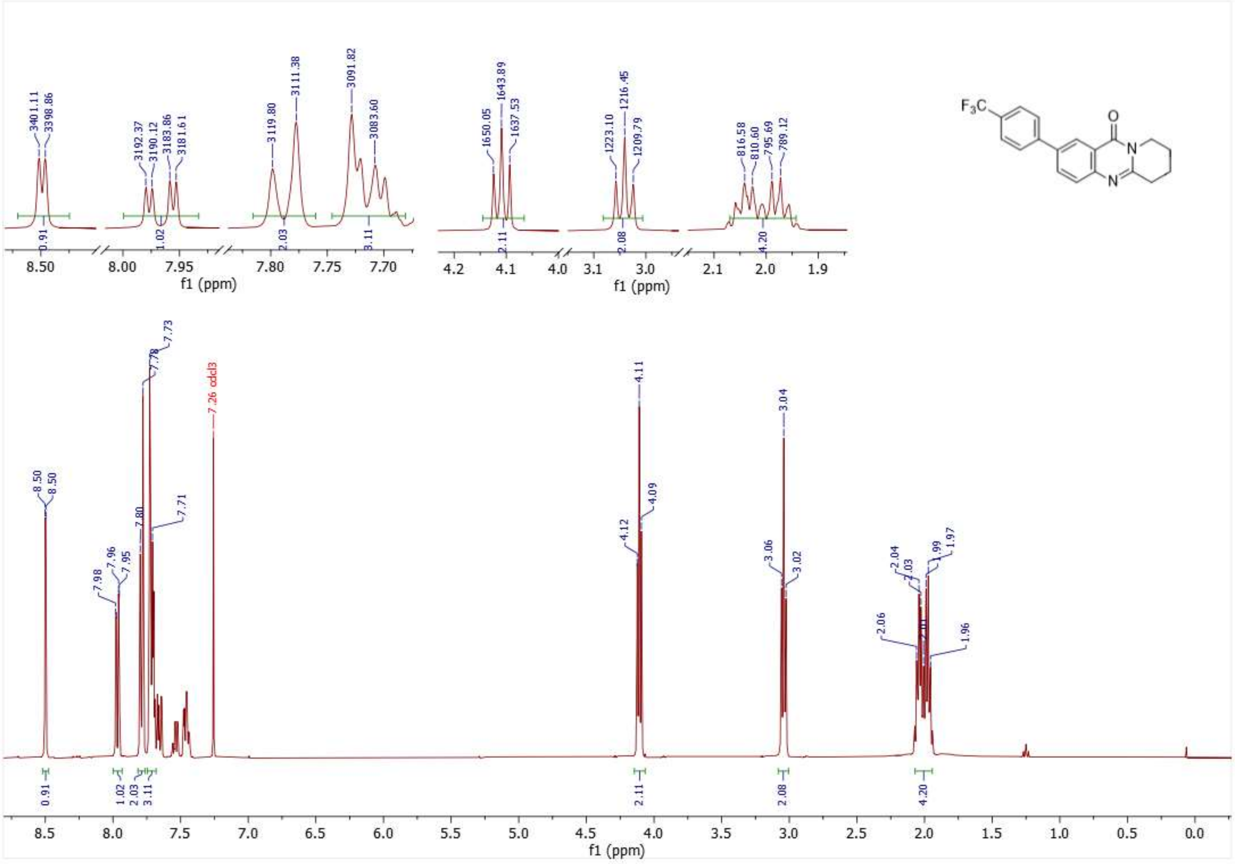

**Figure S49.** $^{1}H$ **NMR spectrum of 4c**

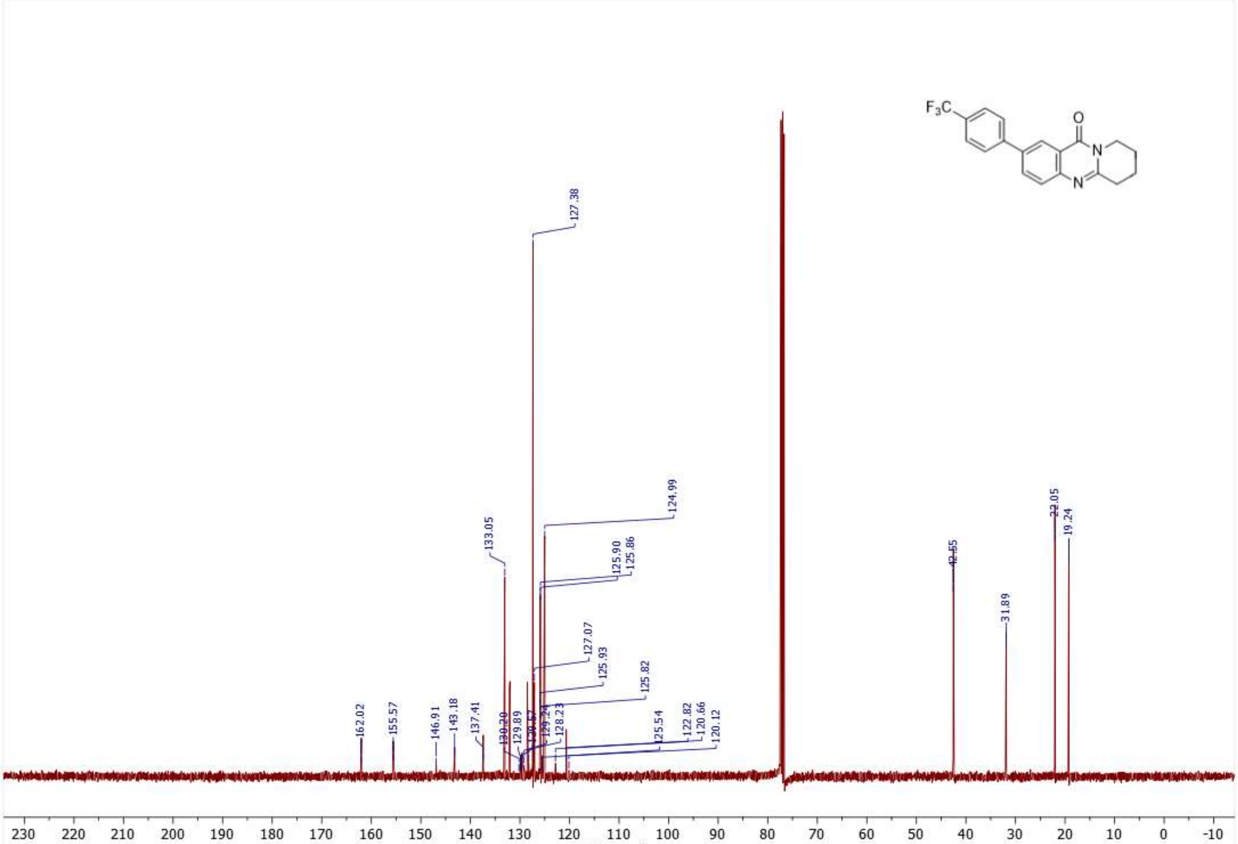

**Figure S50.** $^{13}C$ **NMR spectrum of 4c**

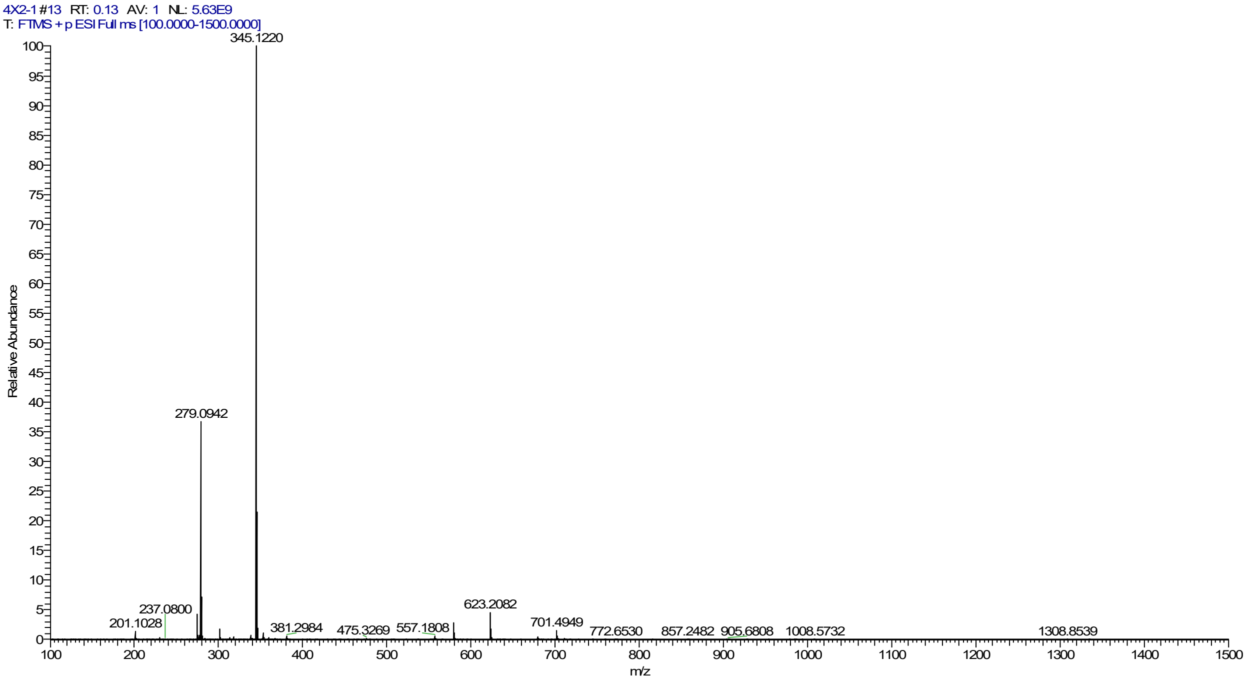


**Figure S51. Mass spectrum of 4c**

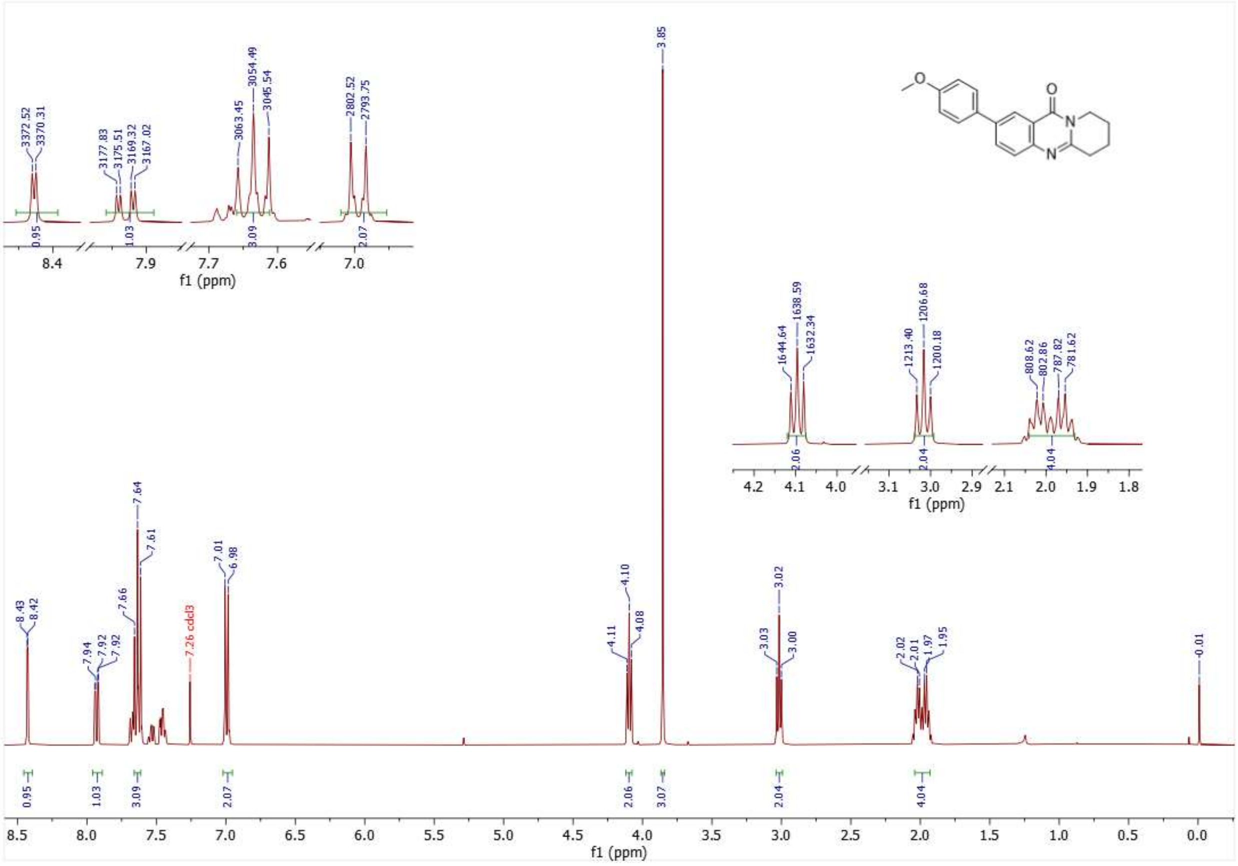


**Figure S52. $^{1}$H NMR spectrum of 4d**

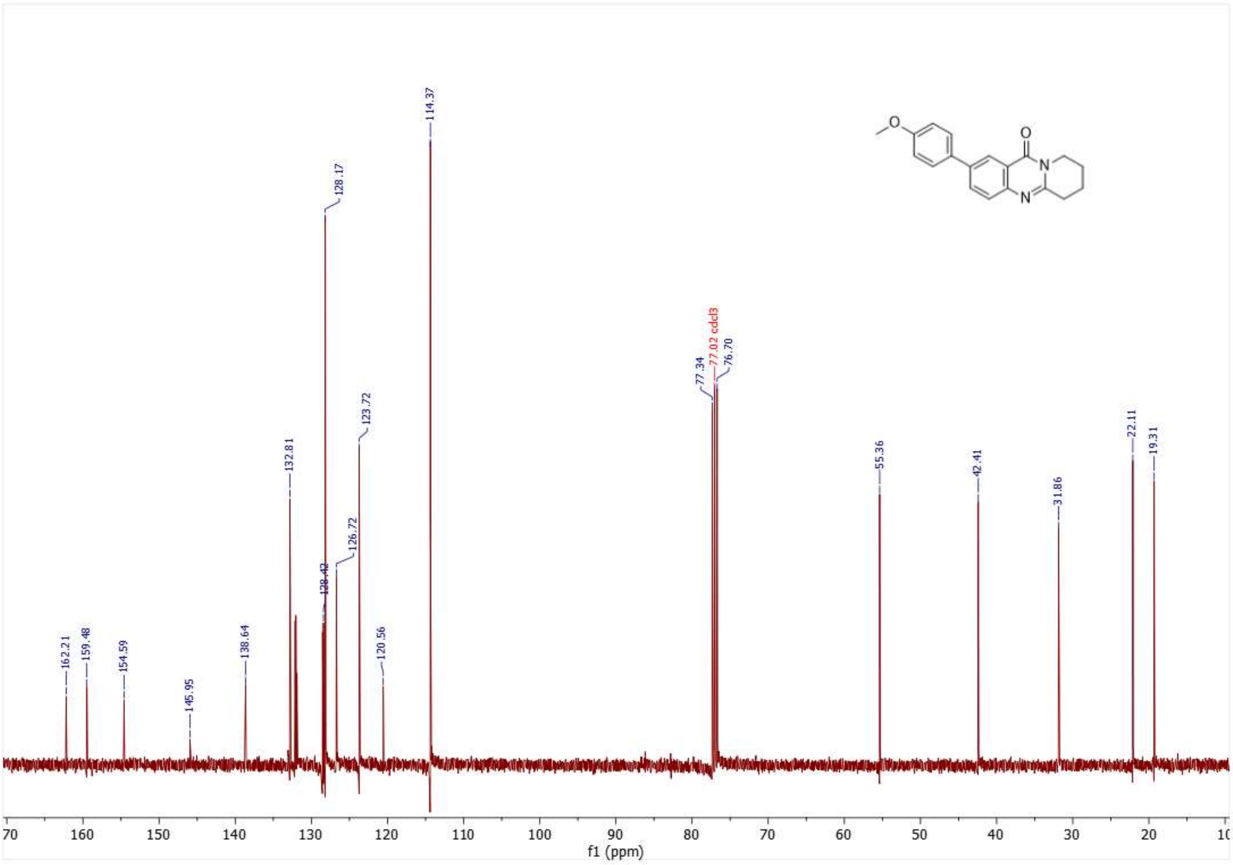


**Figure S53.** $^{13}$**C NMR spectrum of 4d**

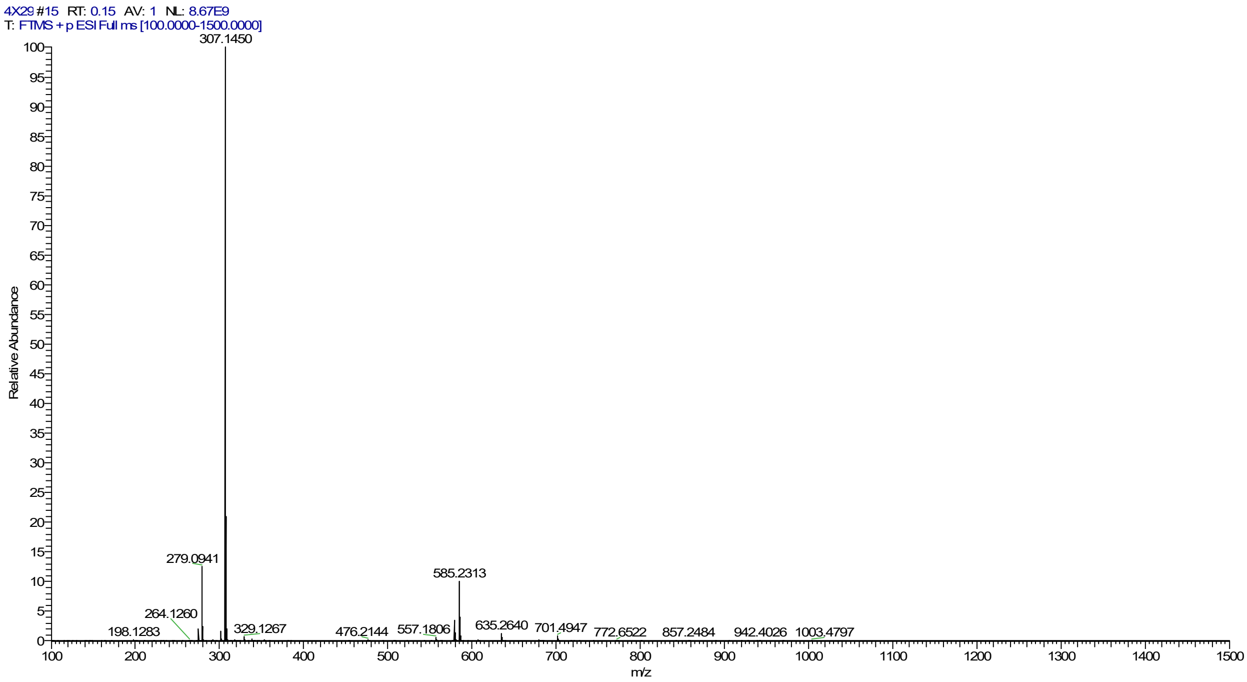


**Figure S54. Mass spectrum of 4d**

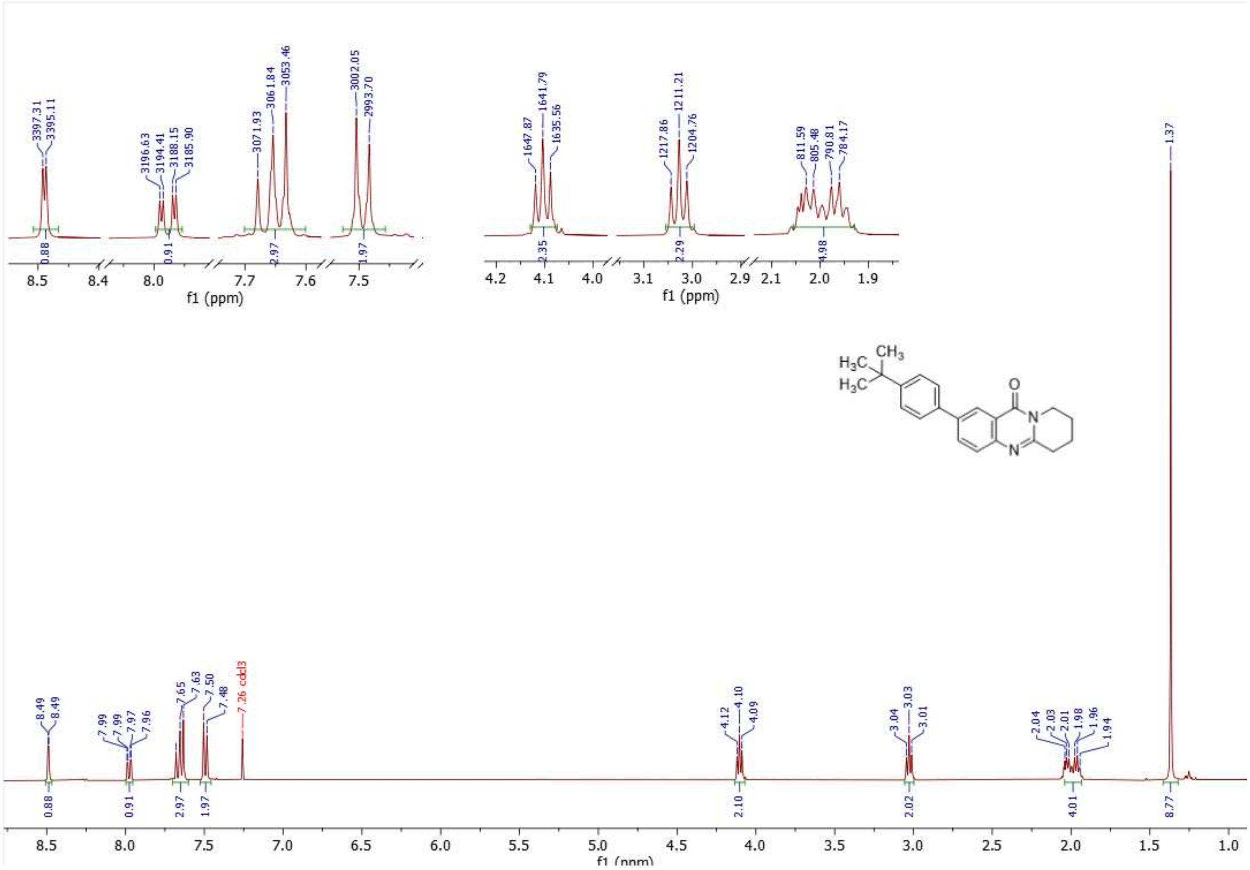

**Figure S55. $^{1}$H NMR spectrum of 4e**

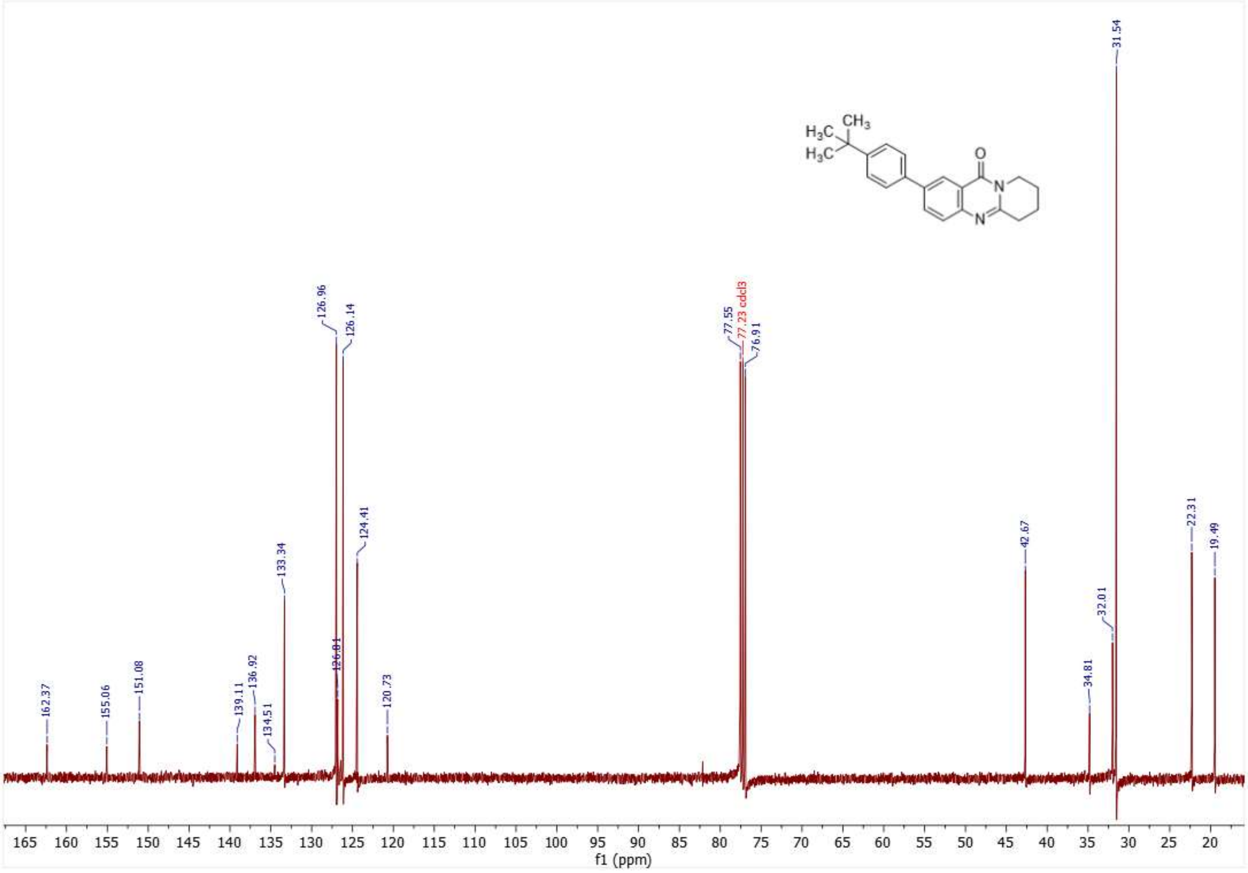

**Figure S56. $^{13}$C NMR spectrum of 4e**

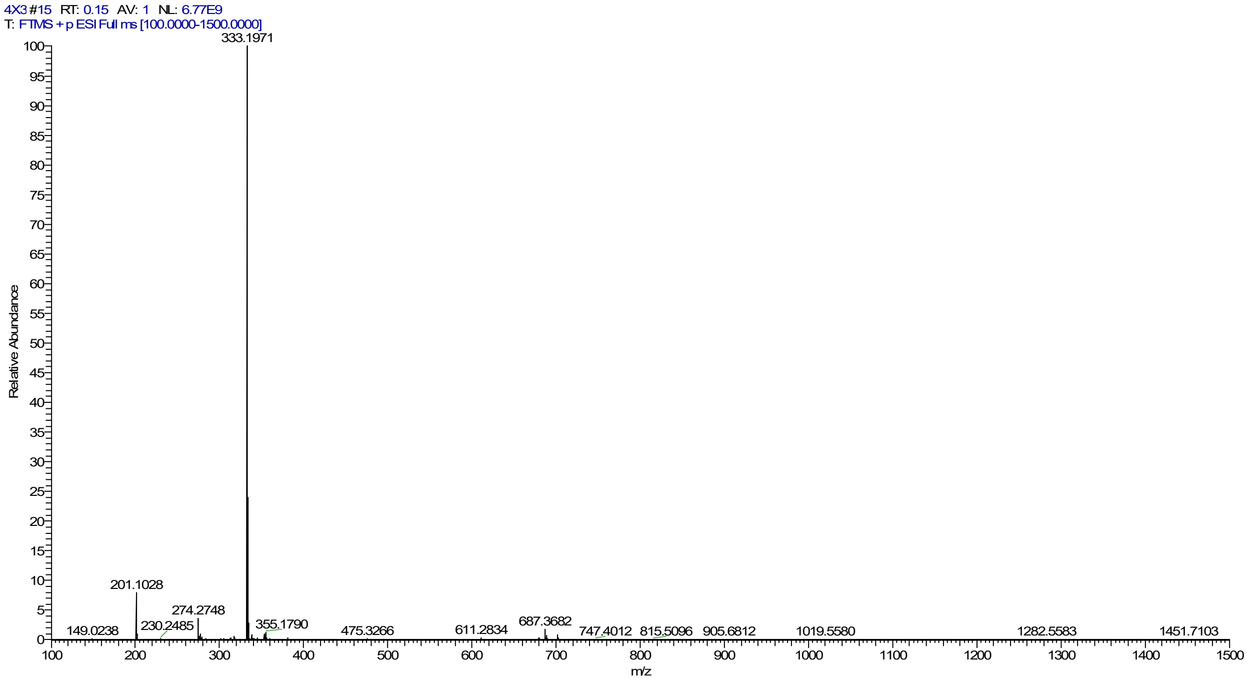


**Figure S57. Mass spectrum of 4e**

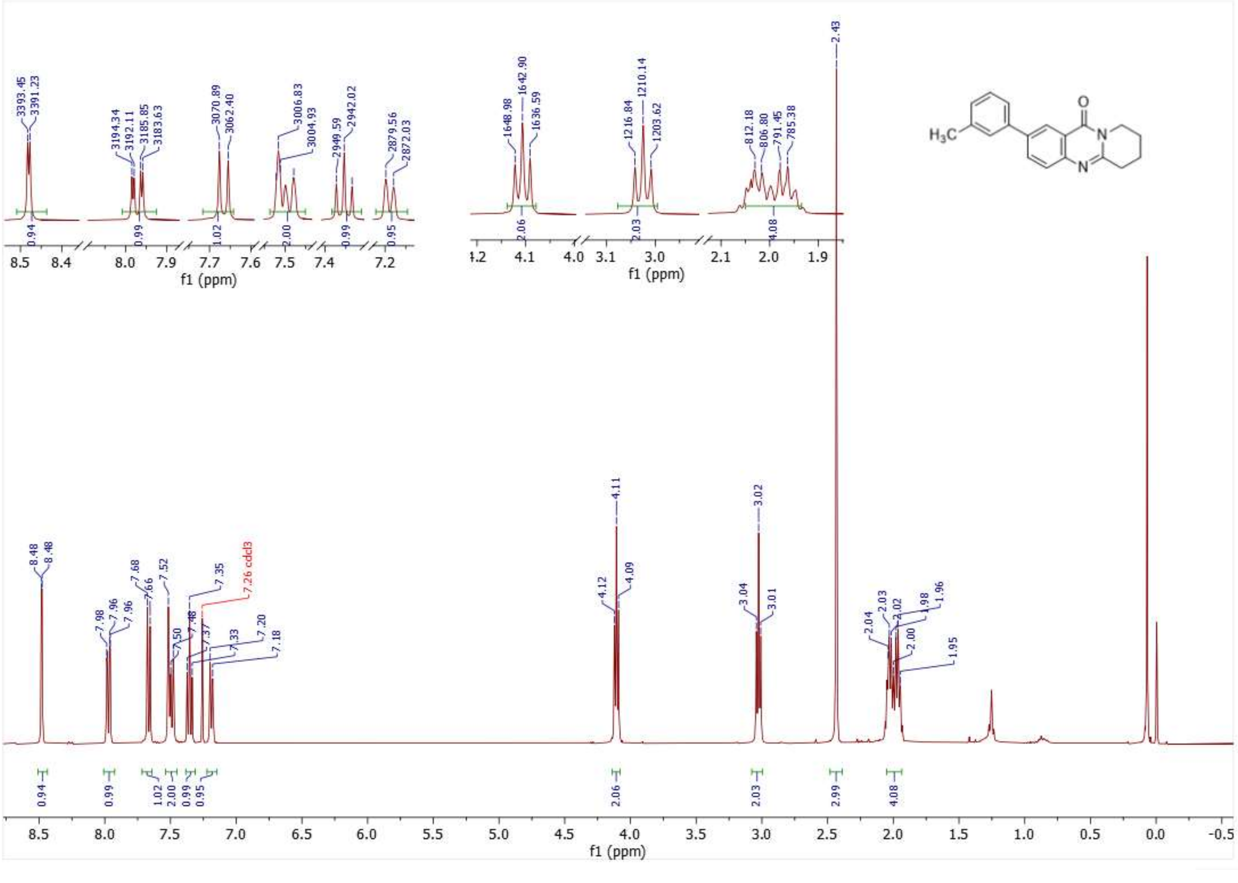


**Figure S58. $^1$H NMR spectrum of 4f**

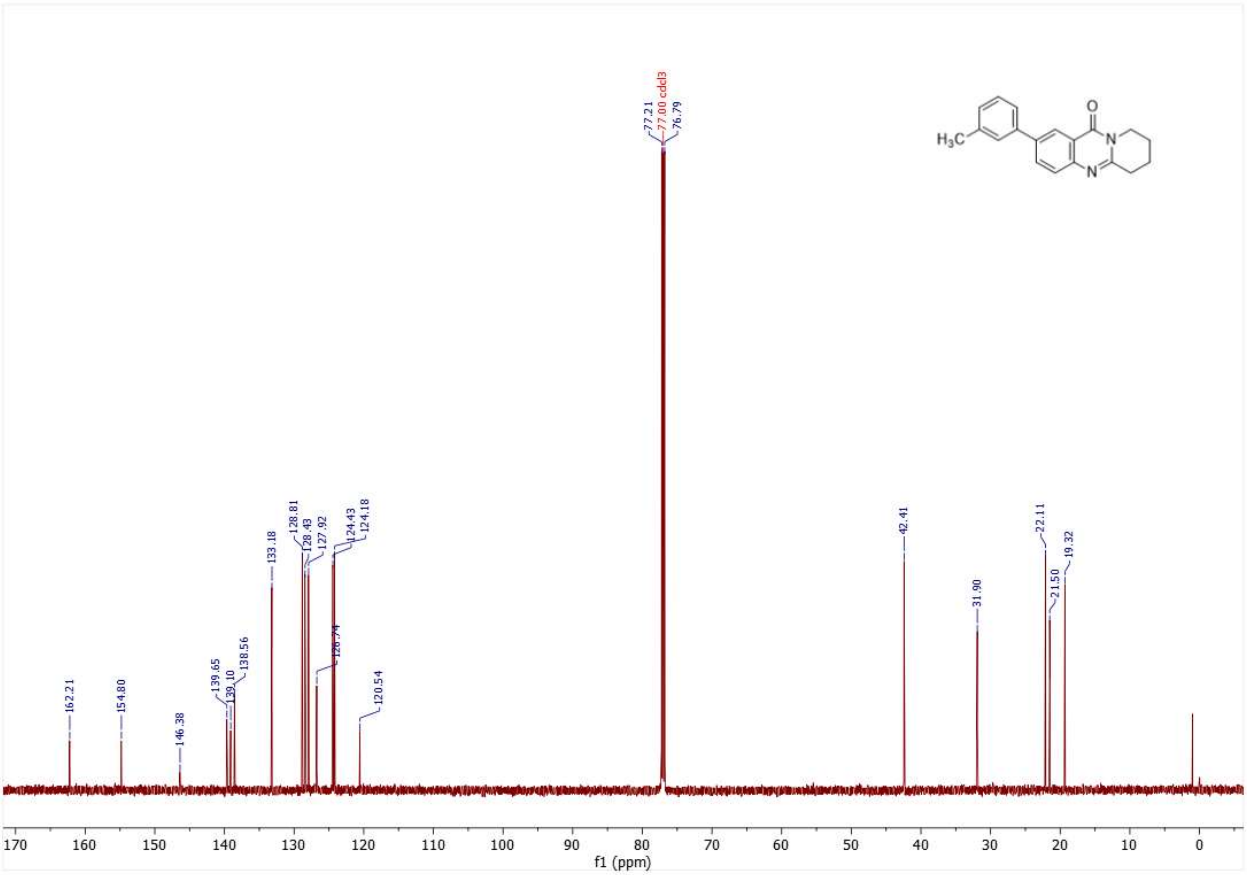


**Figure S59.** $^{13}$**C NMR spectrum of 4f**

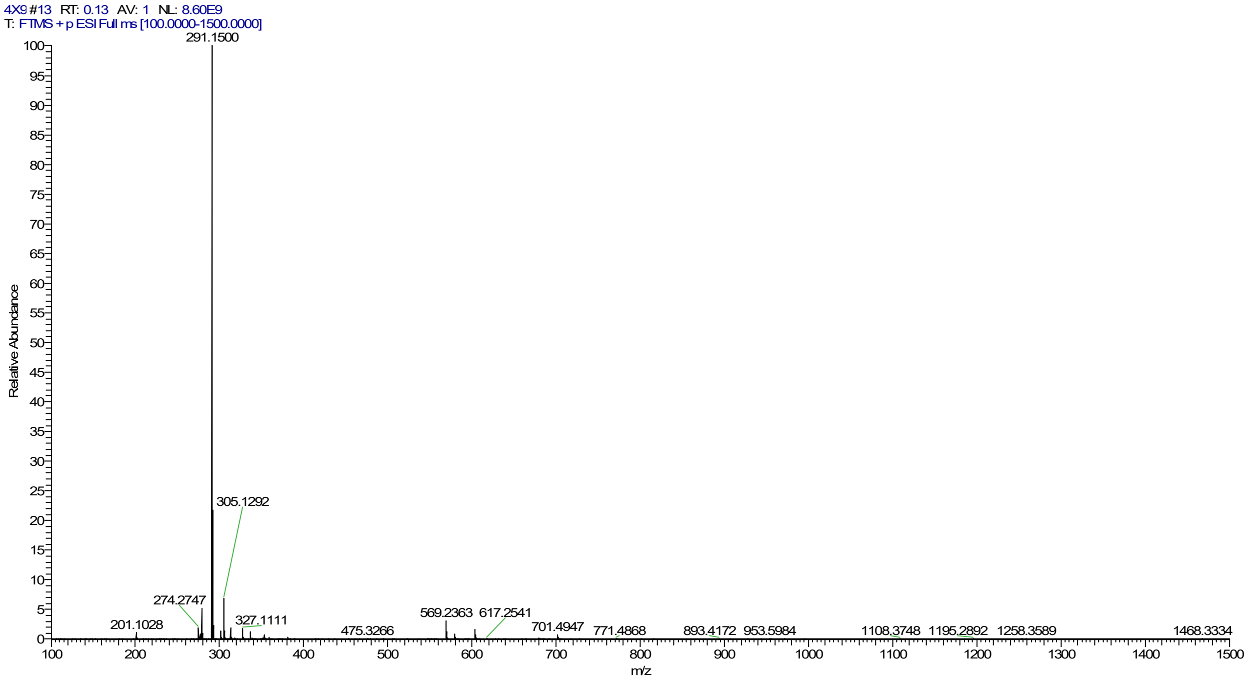


**Figure S60. Mass spectrum of 4f**

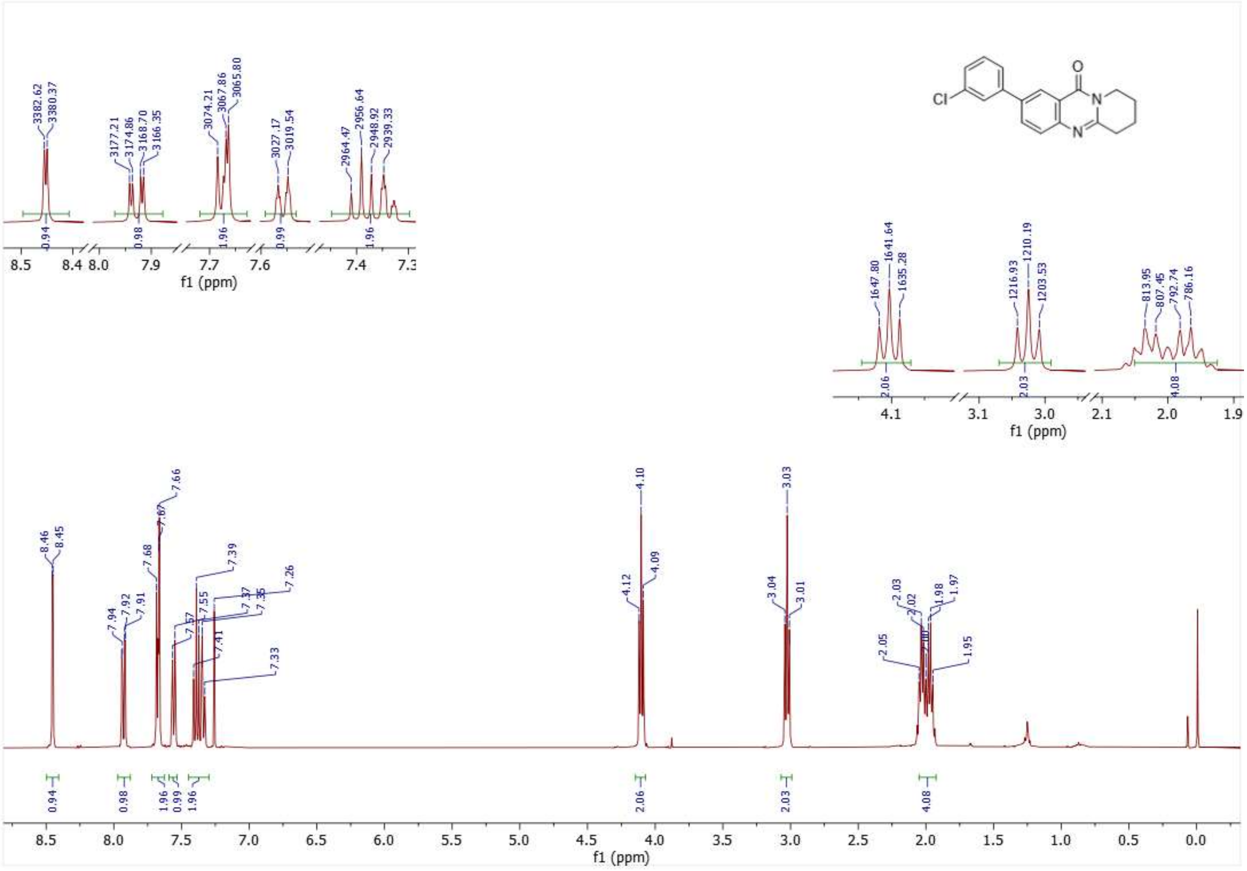

**Figure S61.** $^{1}$**H NMR spectrum of 4g**

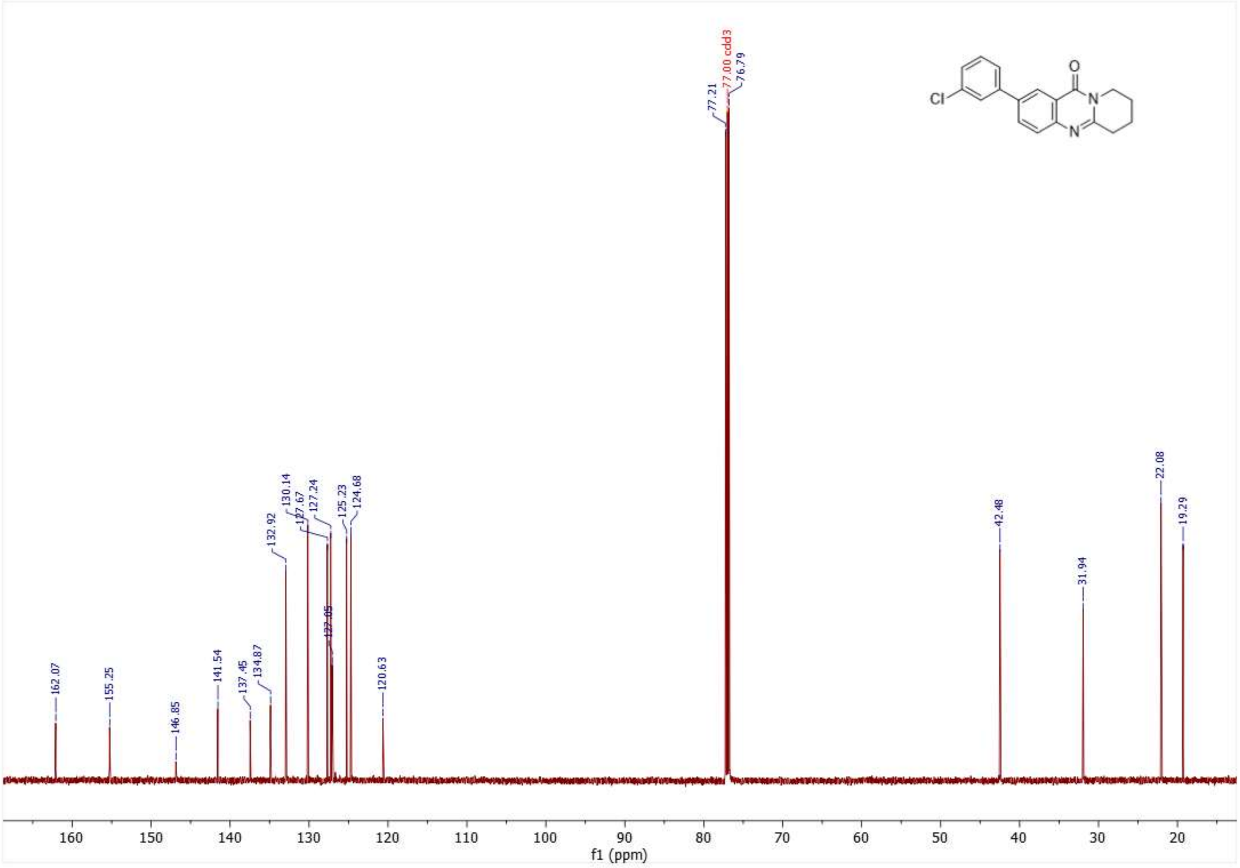

**Figure S62.** $^{13}$**C NMR spectrum of 4g**

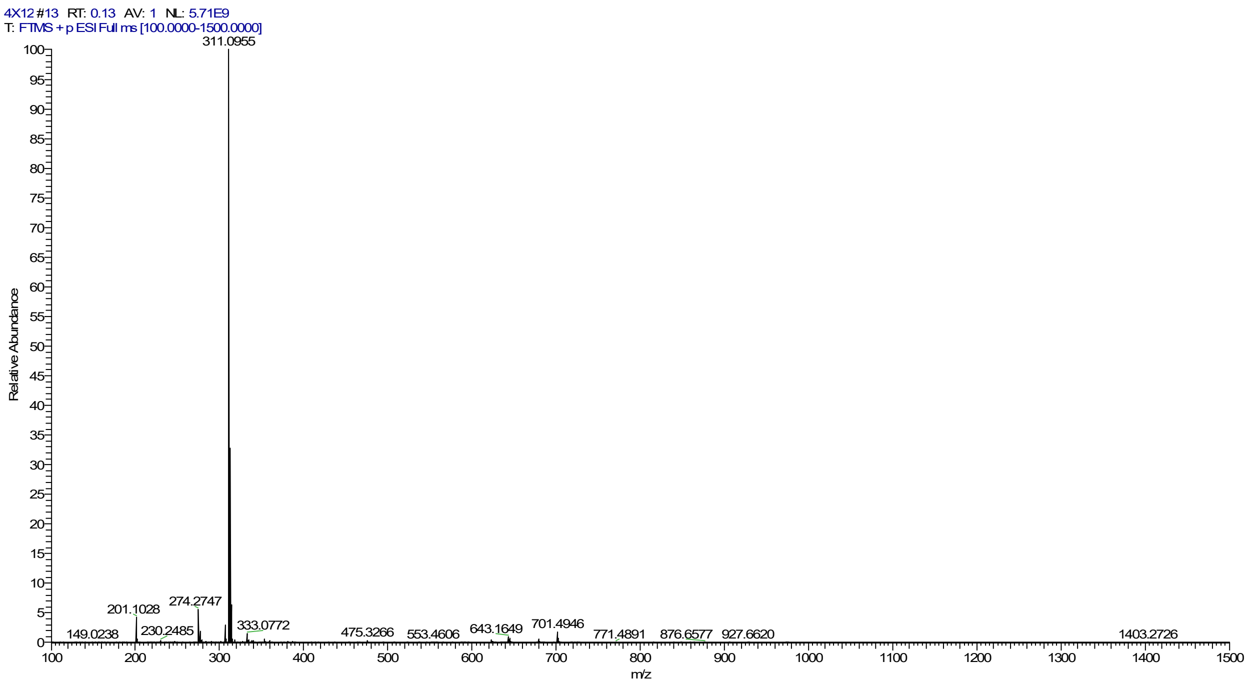


**Figure S63. Mass spectrum of 4g**

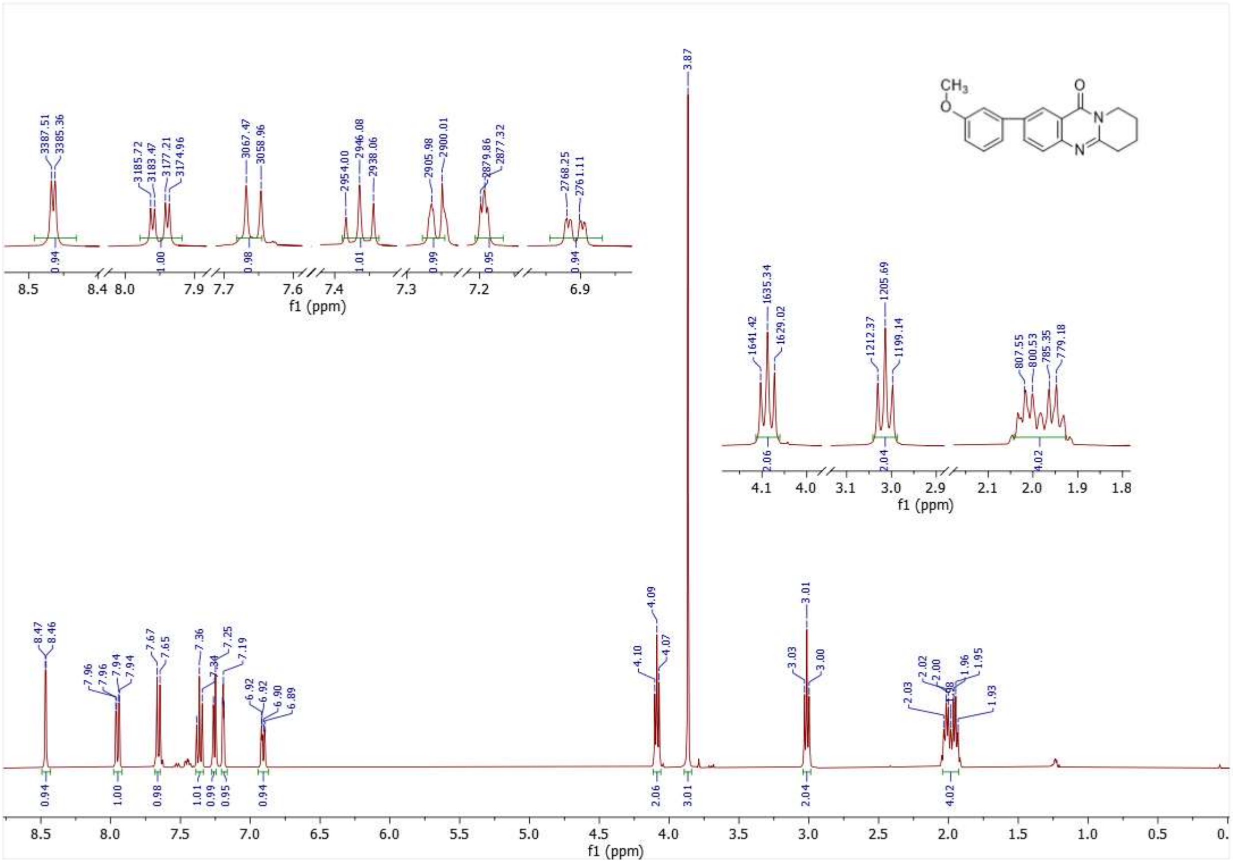


**Figure S64. $^{1}$H NMR spectrum of 4h**

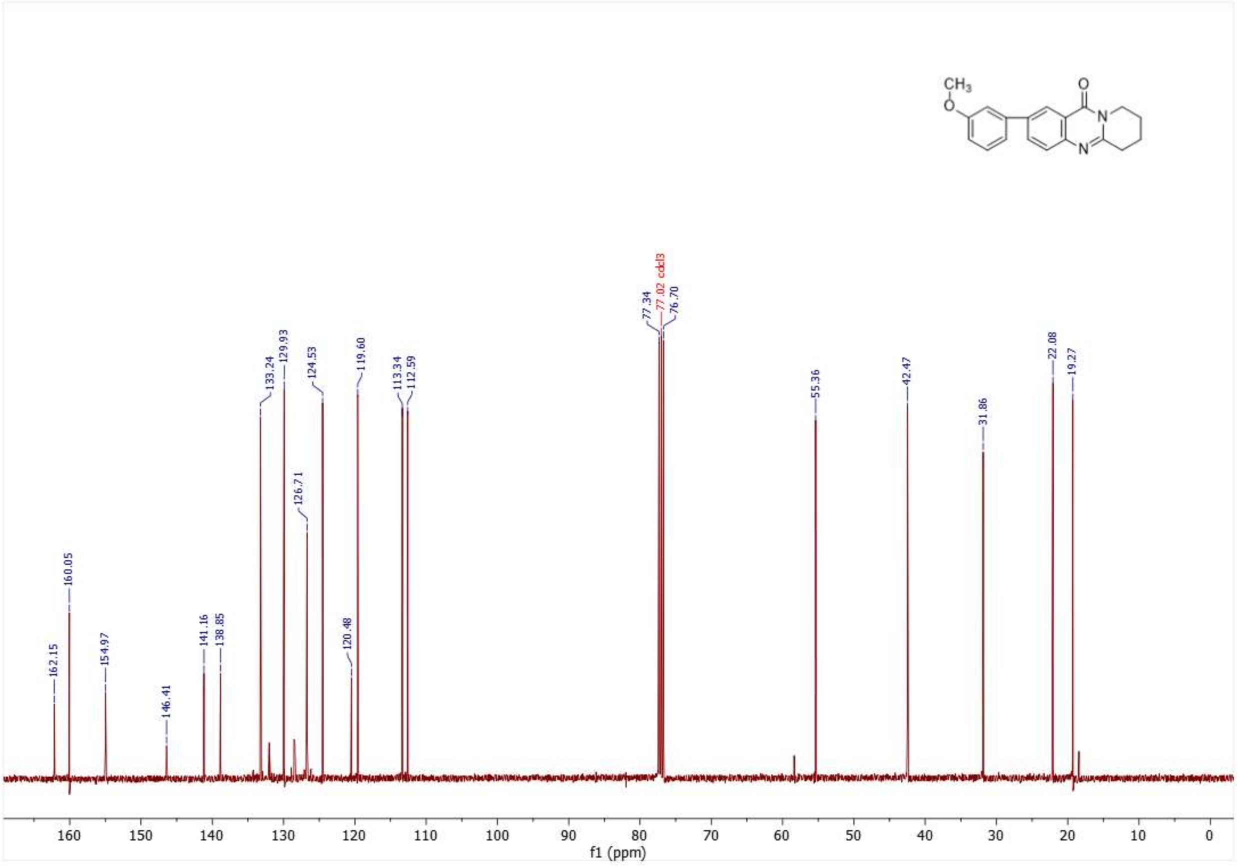


**Figure S65.** $^{13}$**C NMR spectrum of 4h**

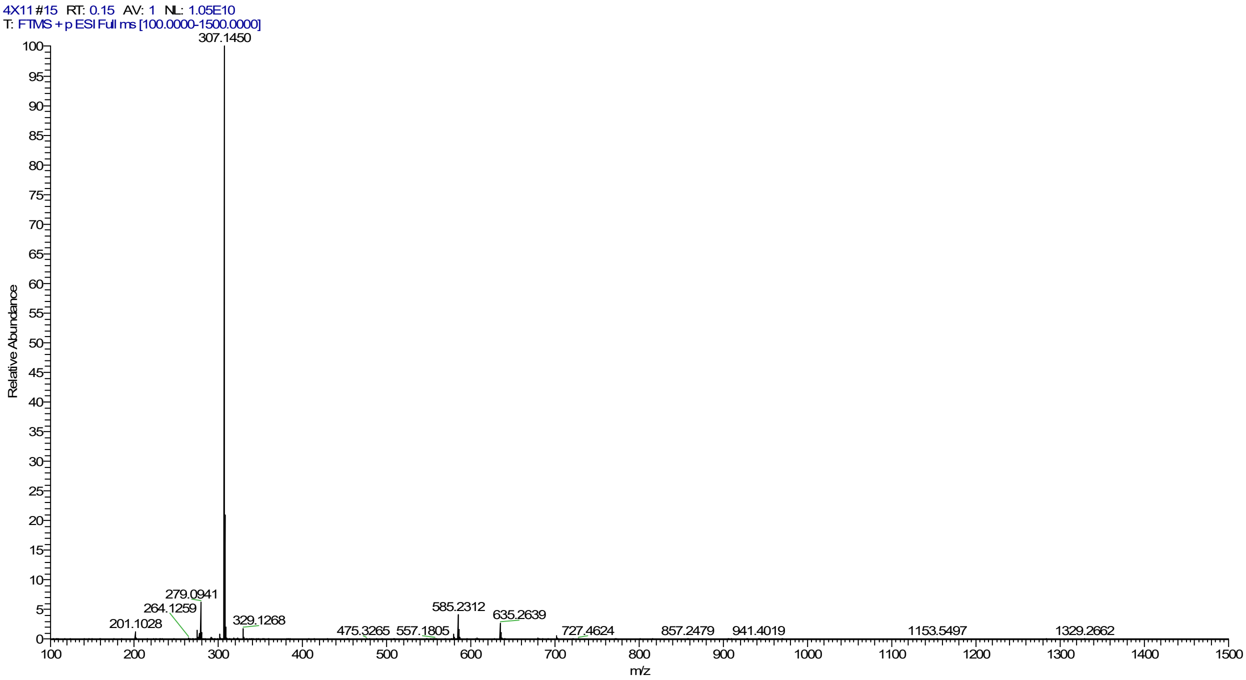


**Figure S66. Mass spectrum of 4h**

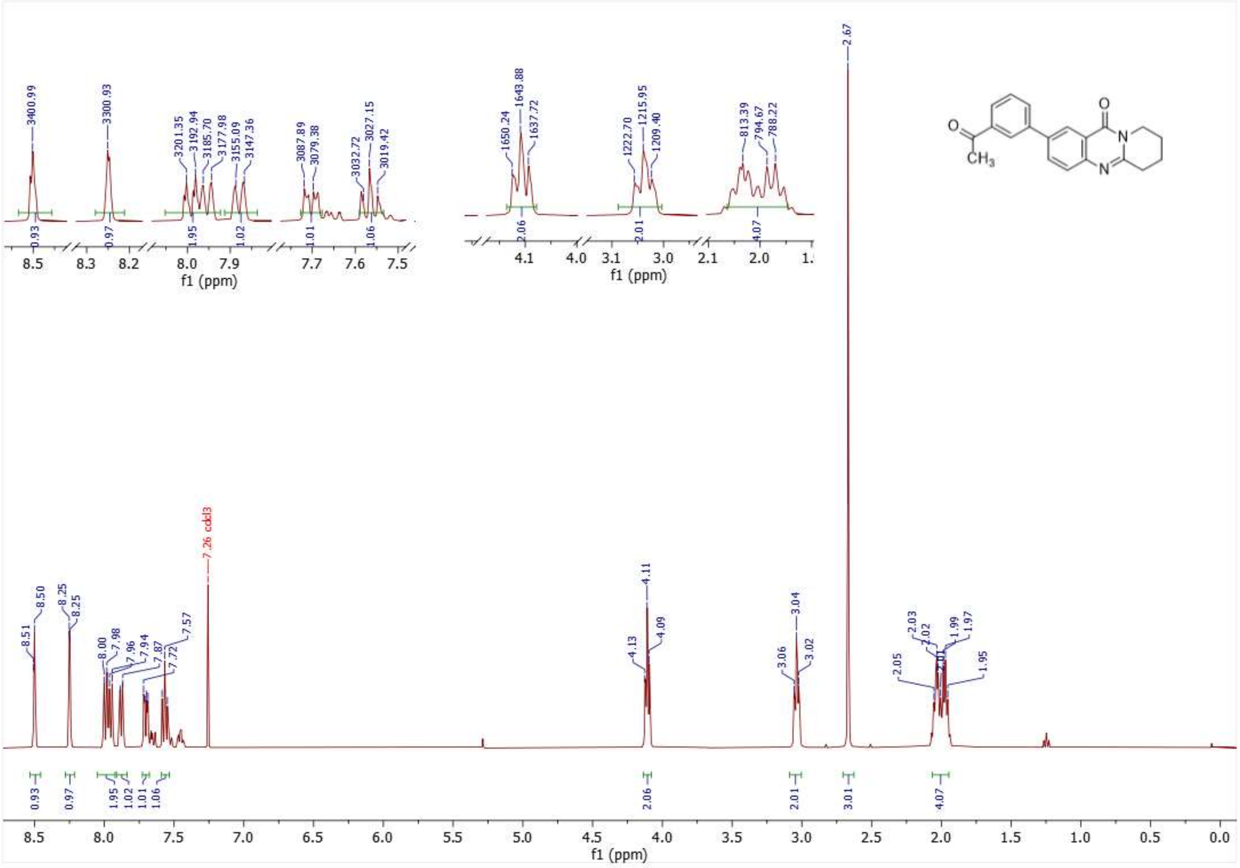


**Figure S67. ¹H NMR spectrum of 4i**

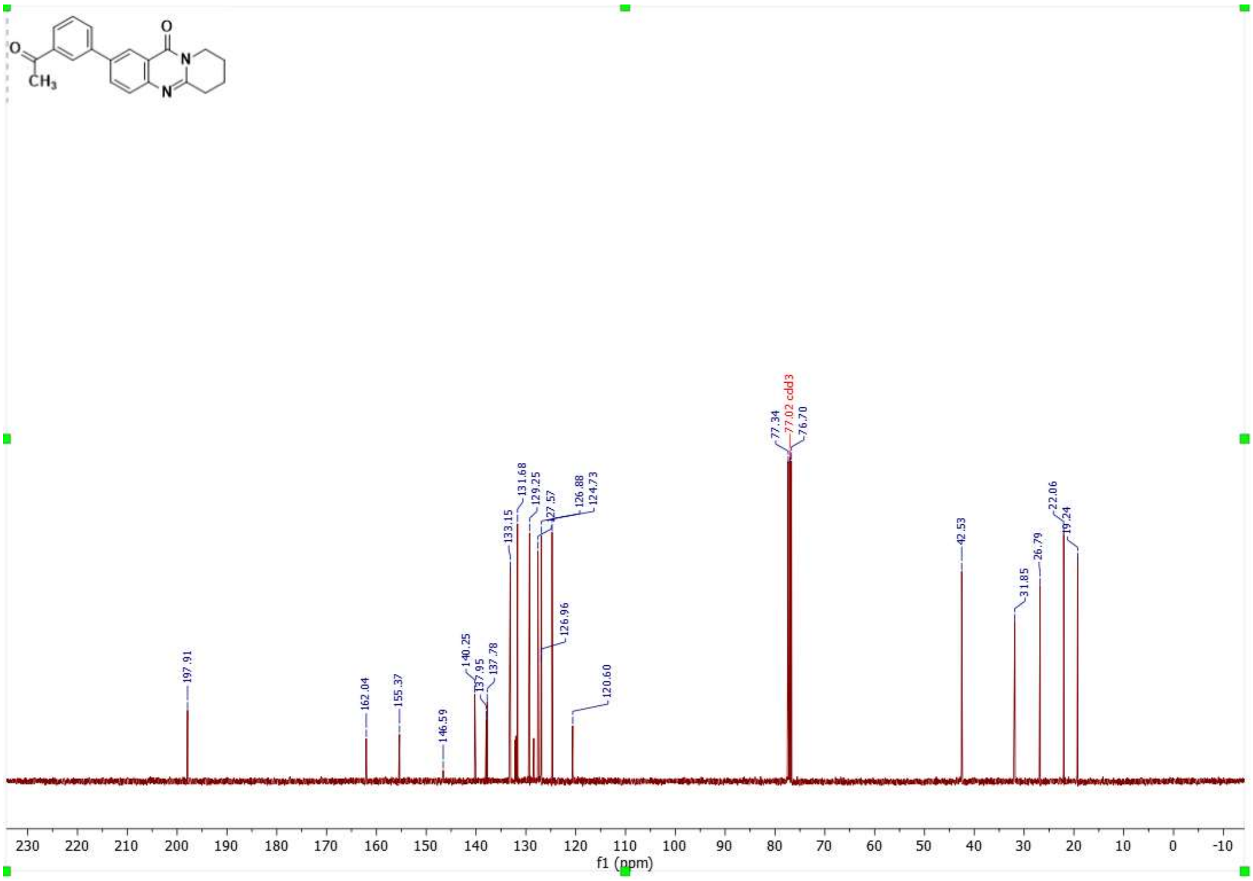


**Figure S68. ¹³C NMR spectrum of 4i**

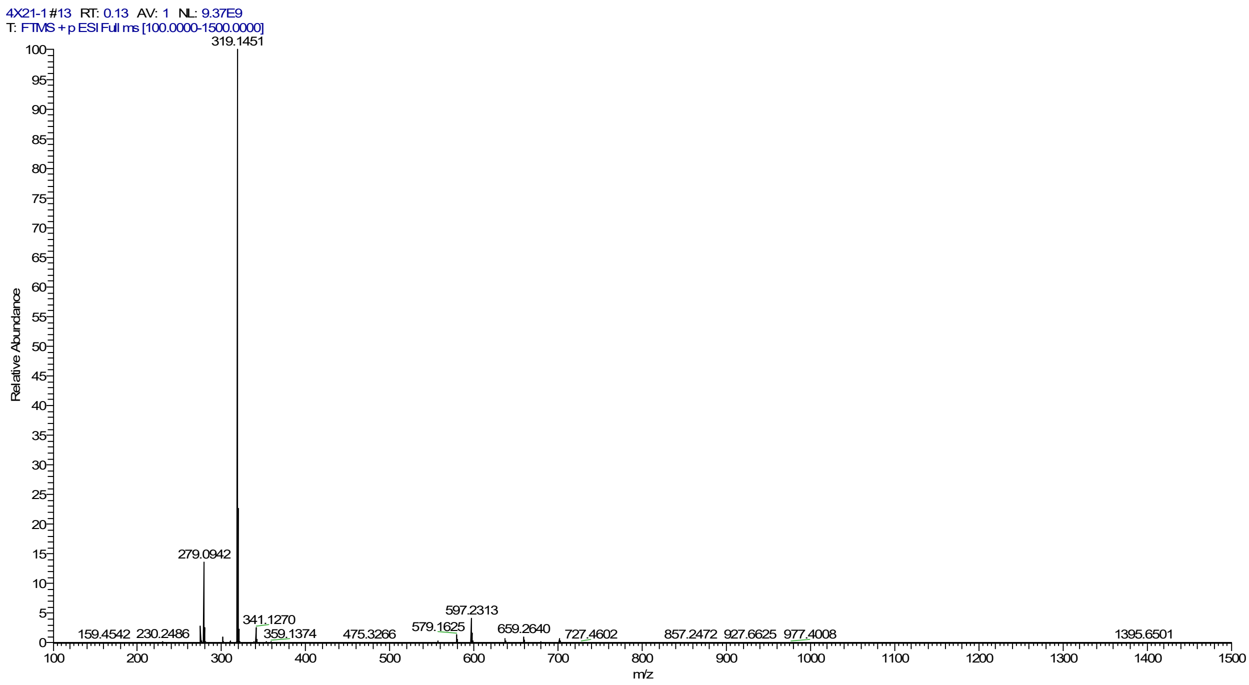

**Figure S69. Mass spectrum of 4i**

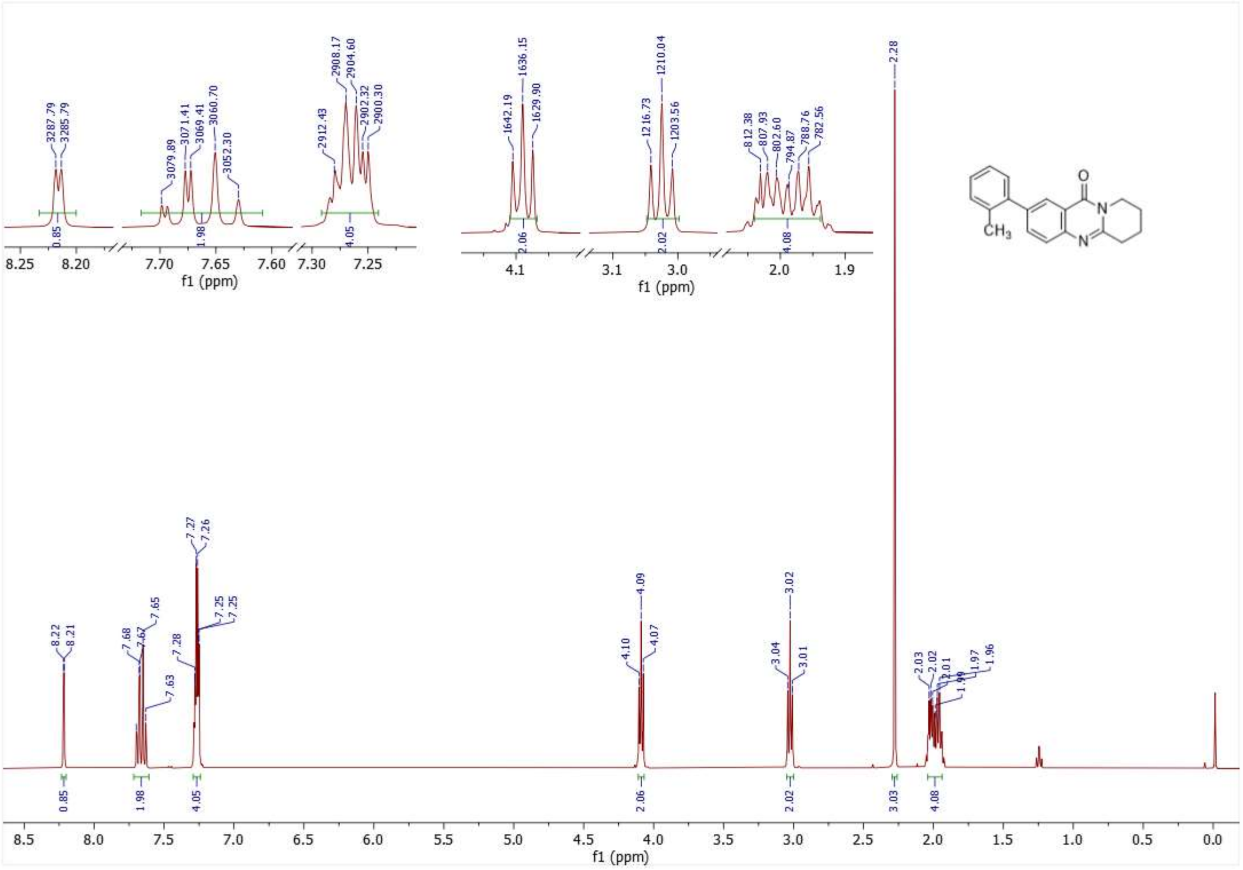

**Figure S70. $^{1}$H NMR spectrum of 4j**

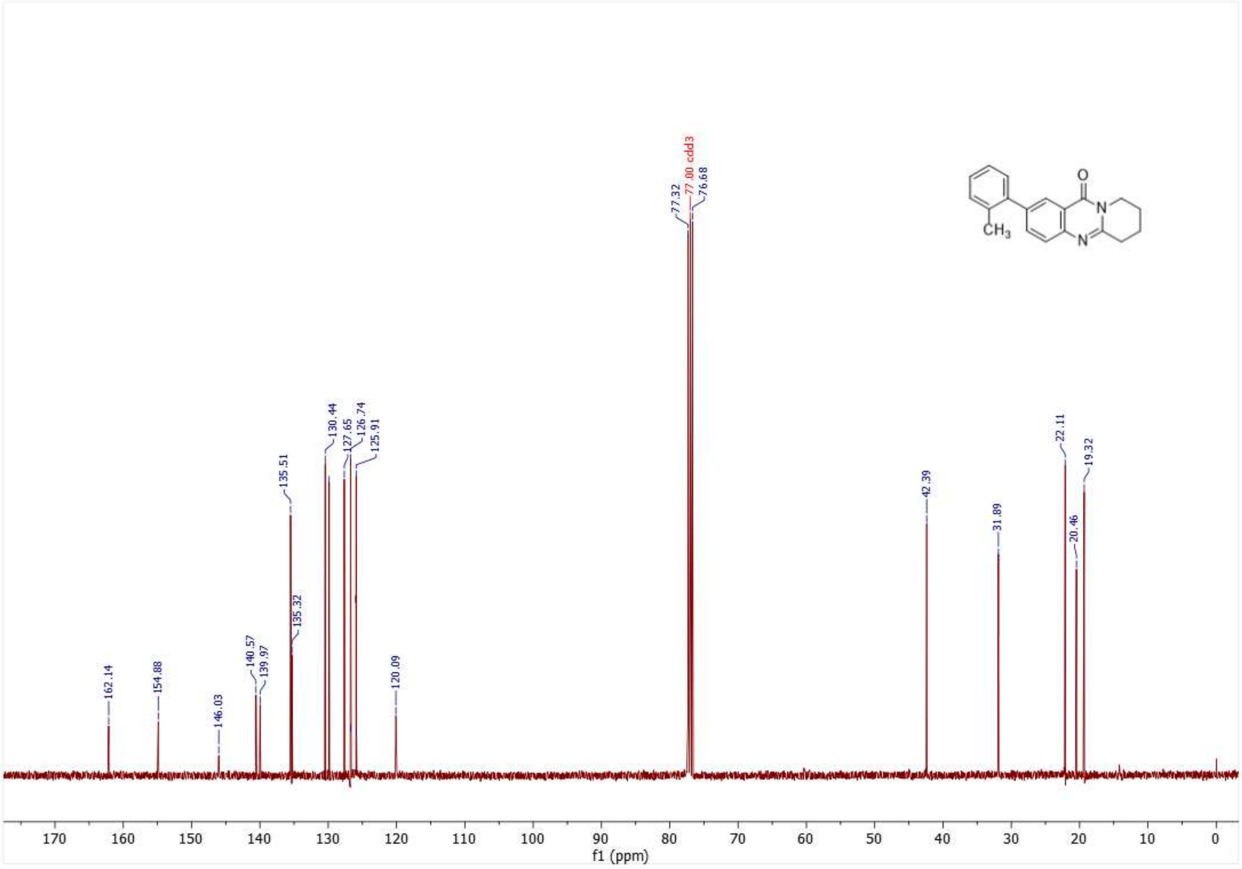


**Figure S71.** $^{13}$**C NMR spectrum of 4j**

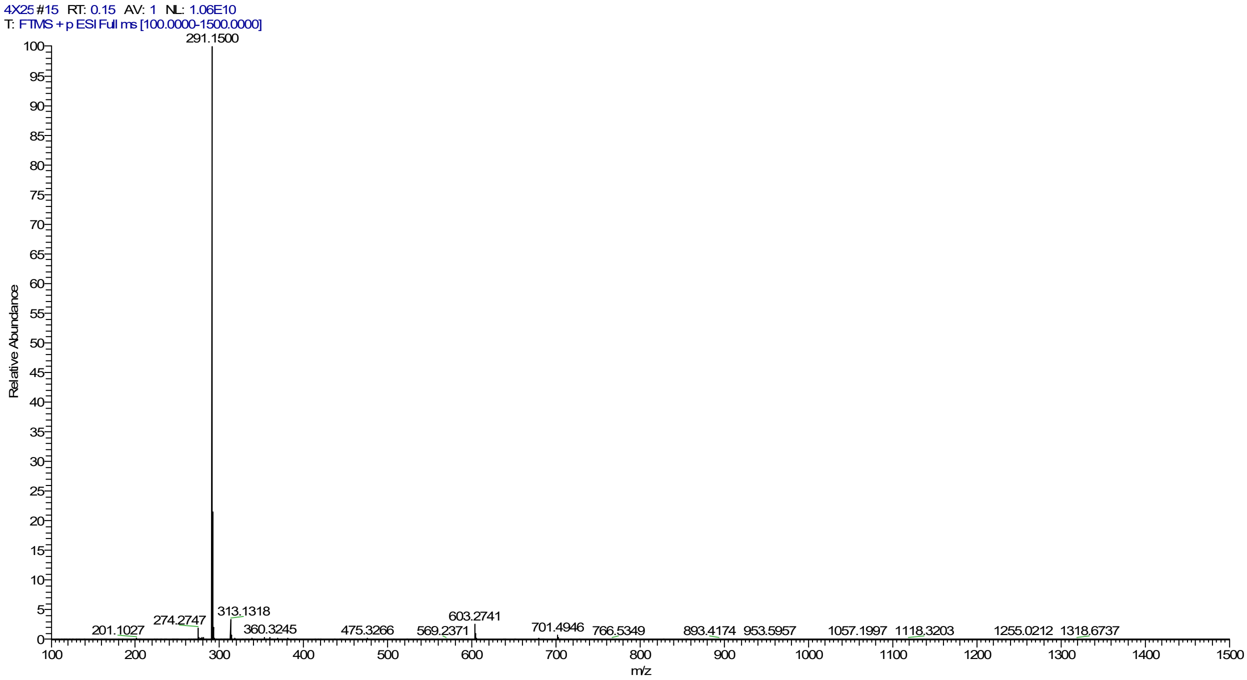


**Figure S72. Mass spectrum of 4j**

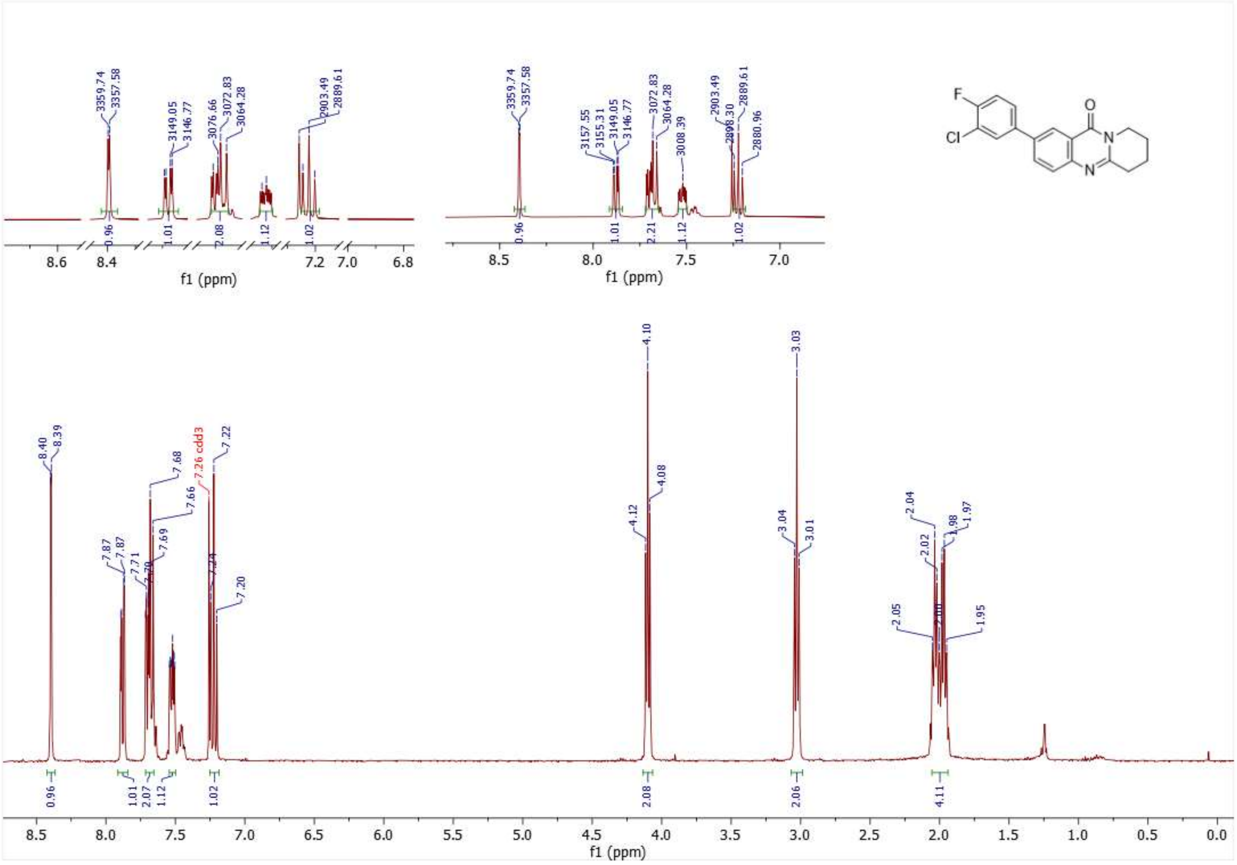

**Figure S73. $^{1}$H NMR spectrum of 4k**

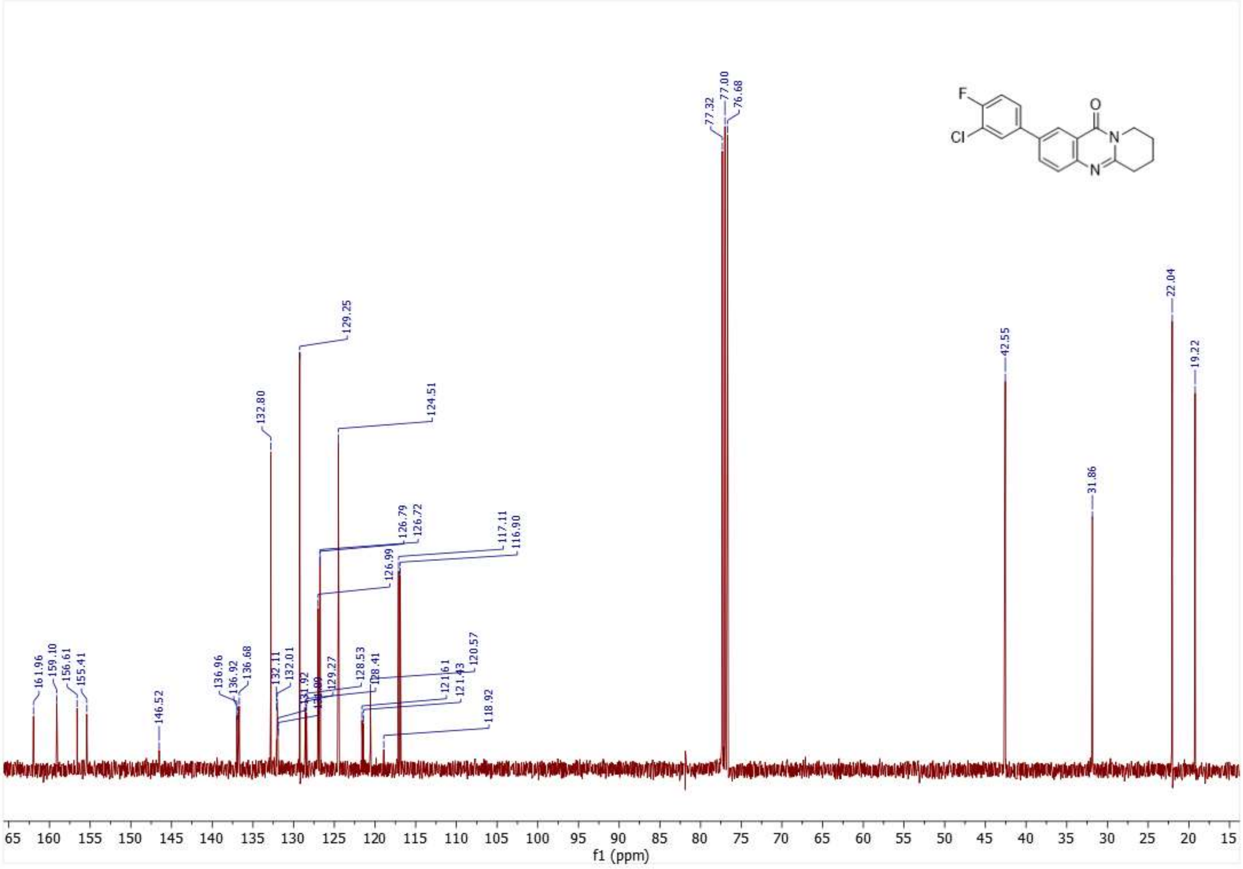

**Figure S74. $^{13}$C NMR spectrum of 4k**

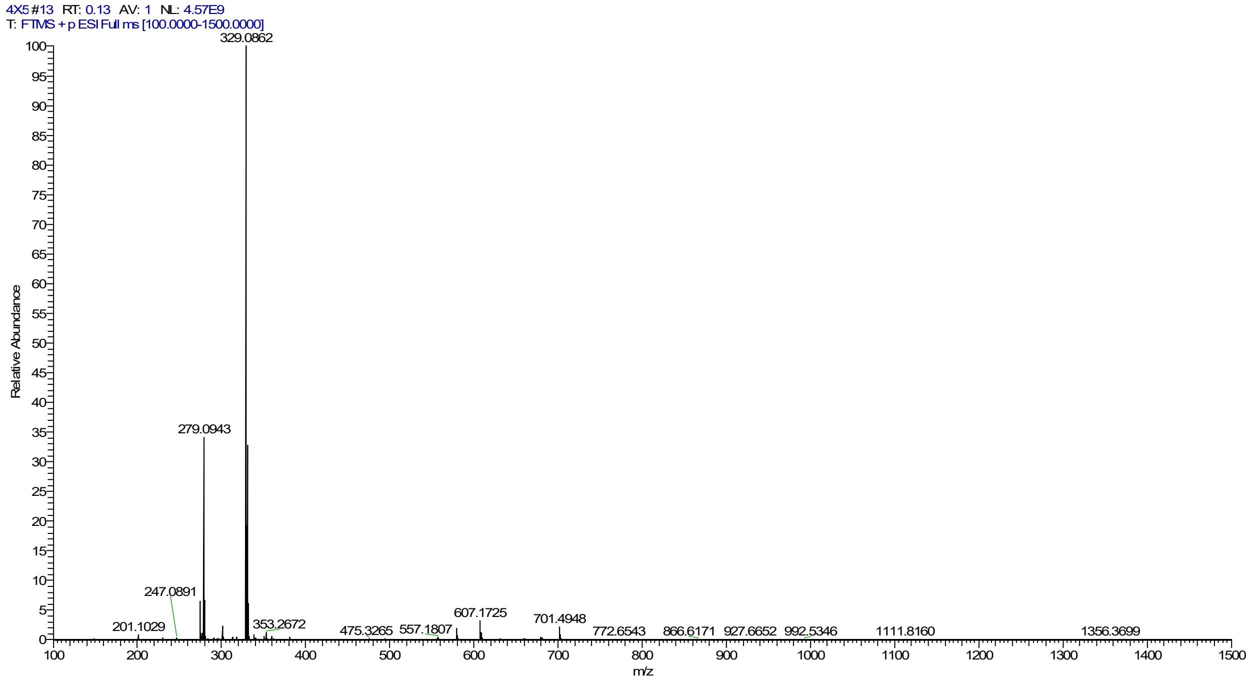


**Figure S75. Mass spectrum of 4k**

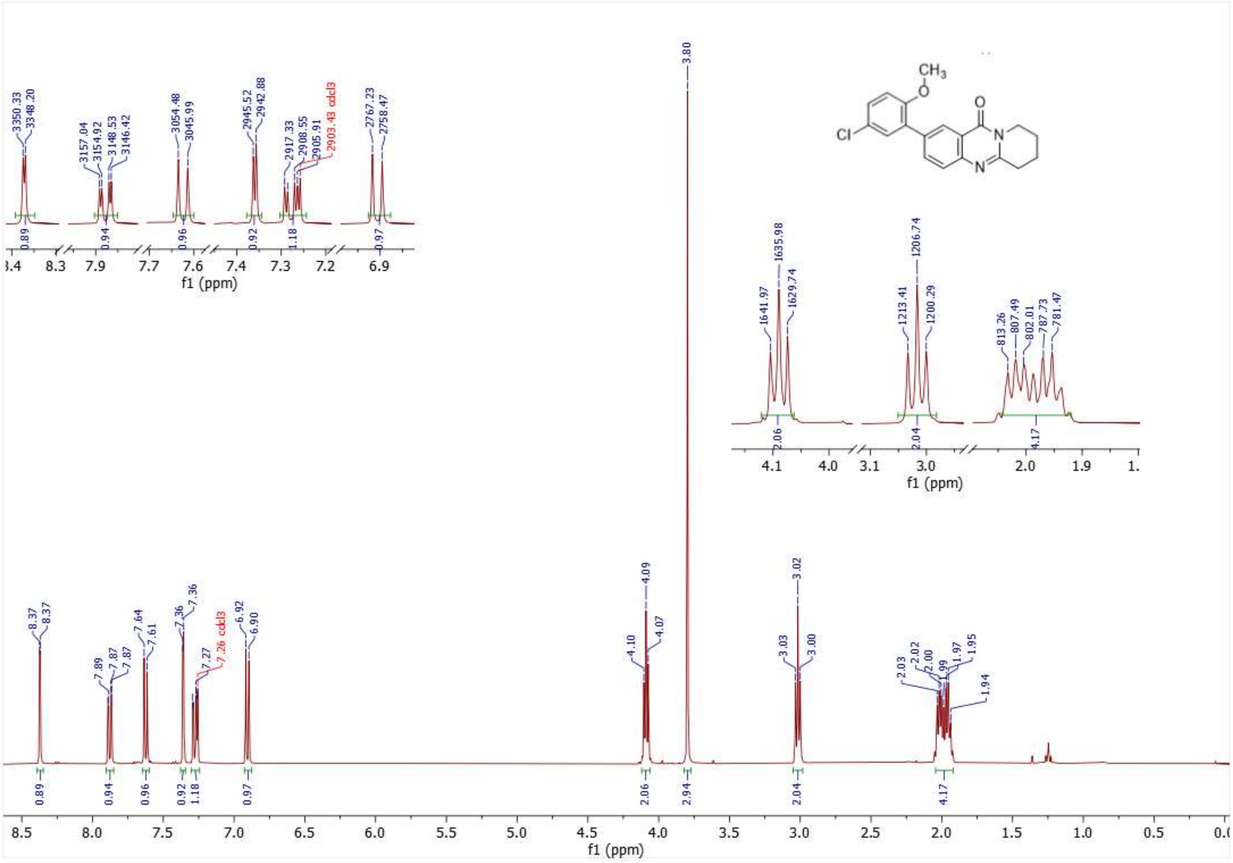


**Figure S76. $^{1}$H NMR spectrum of 4l**

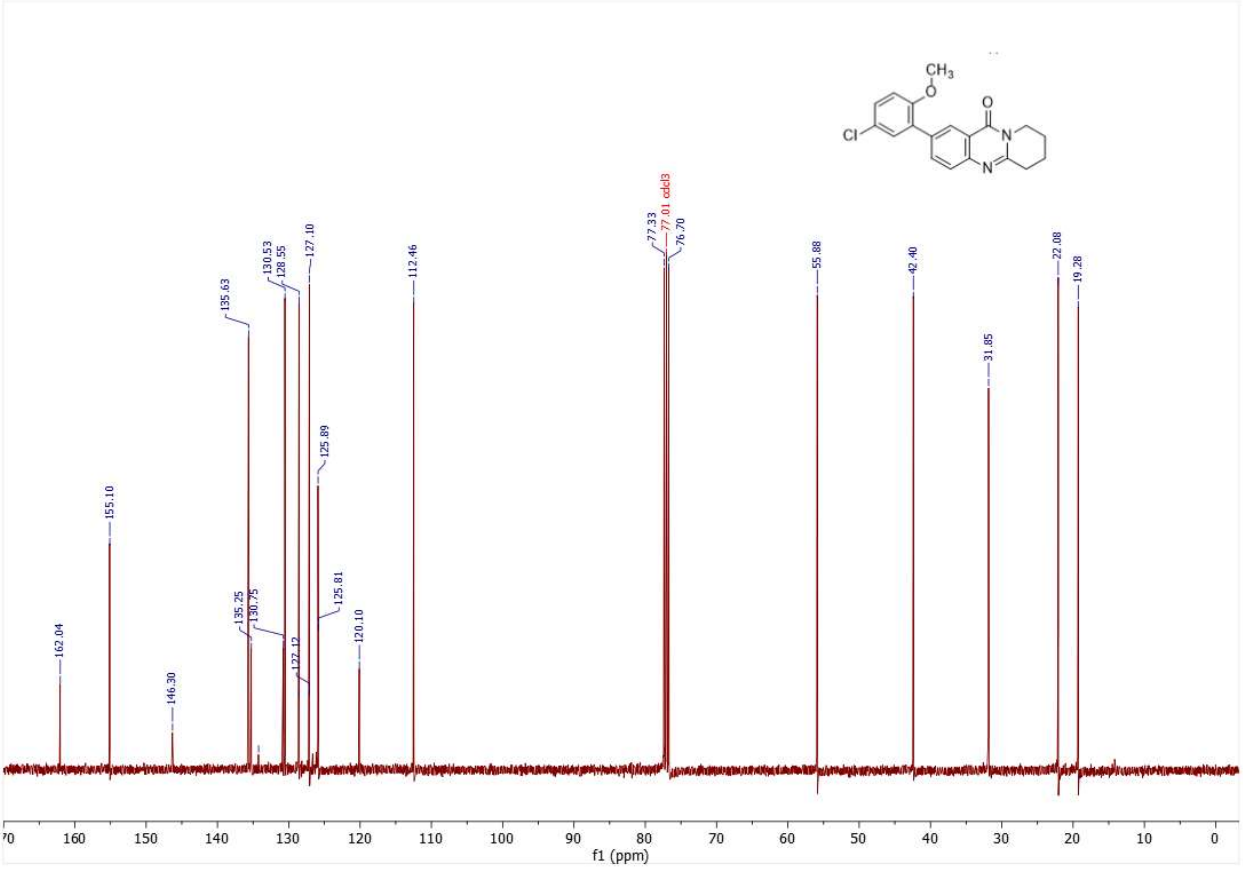


Figure S77. $^{13}$C NMR spectrum of 4l

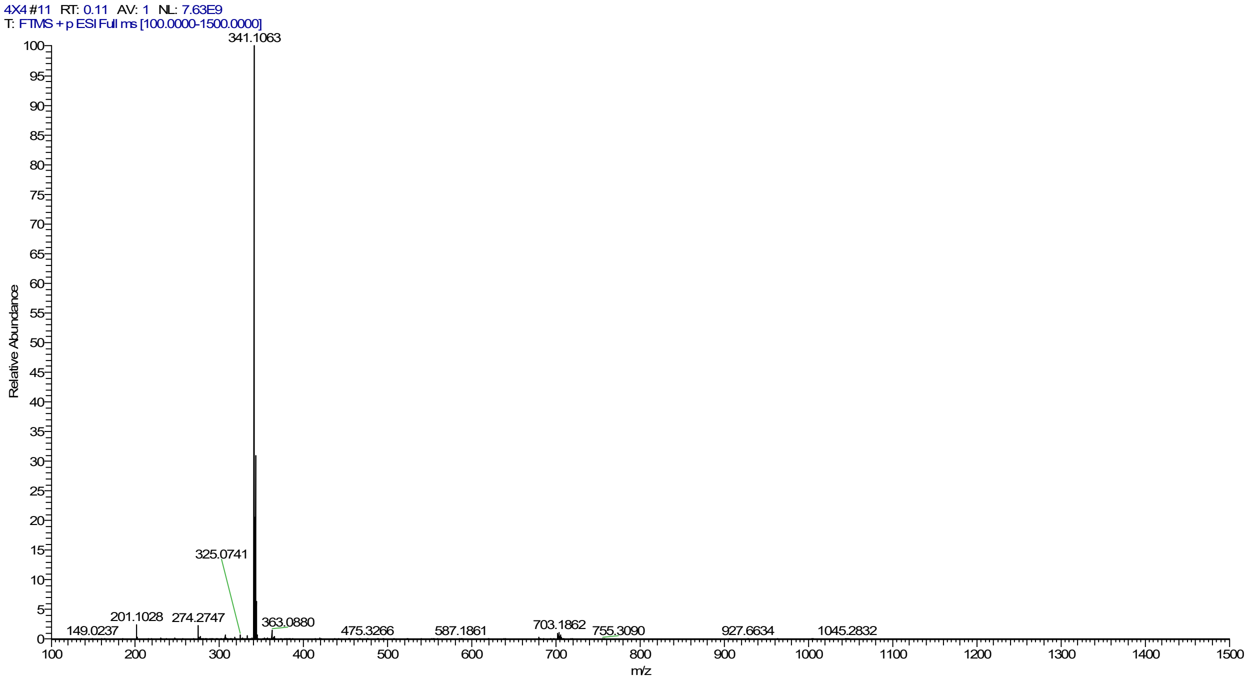


Figure S78. Mass spectrum of 4l

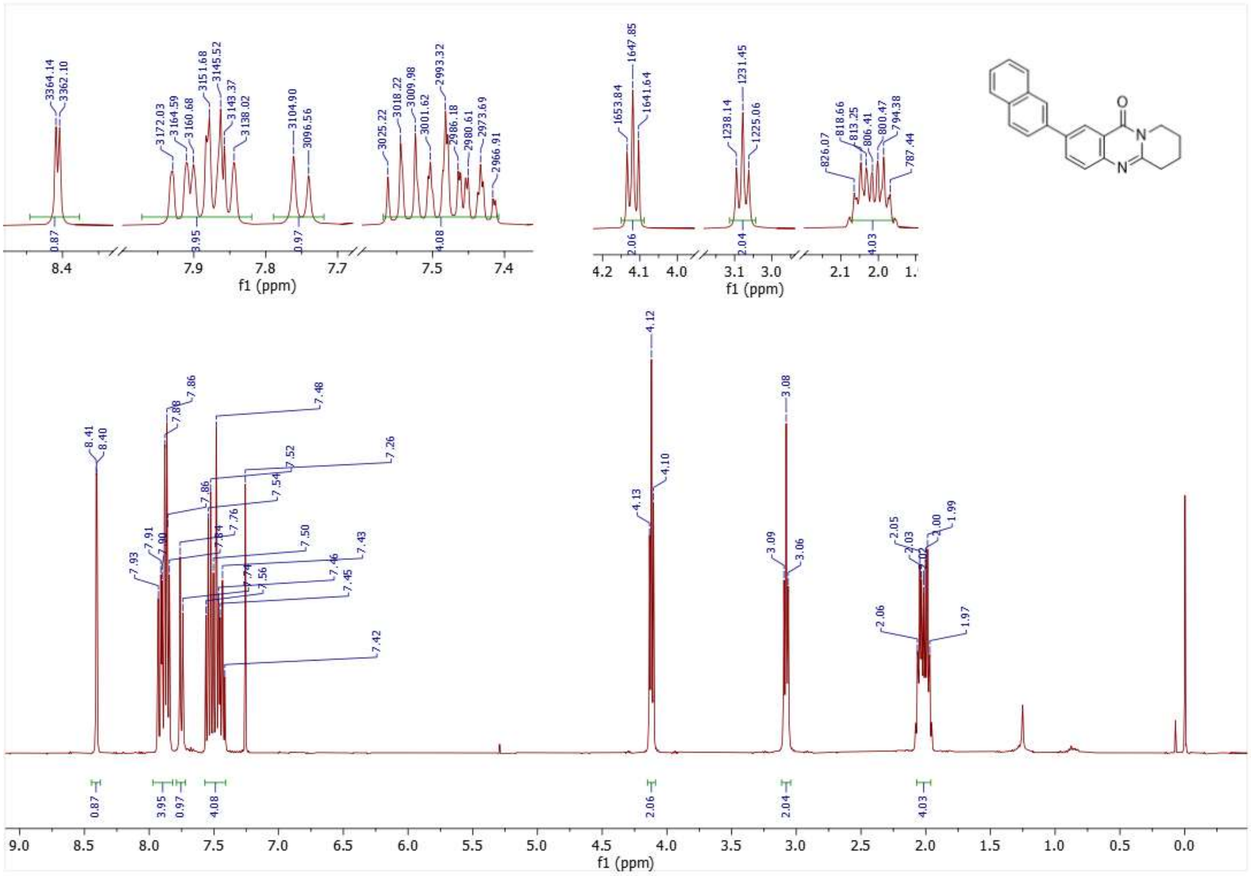

**Figure S79. ¹H NMR spectrum of 4m**

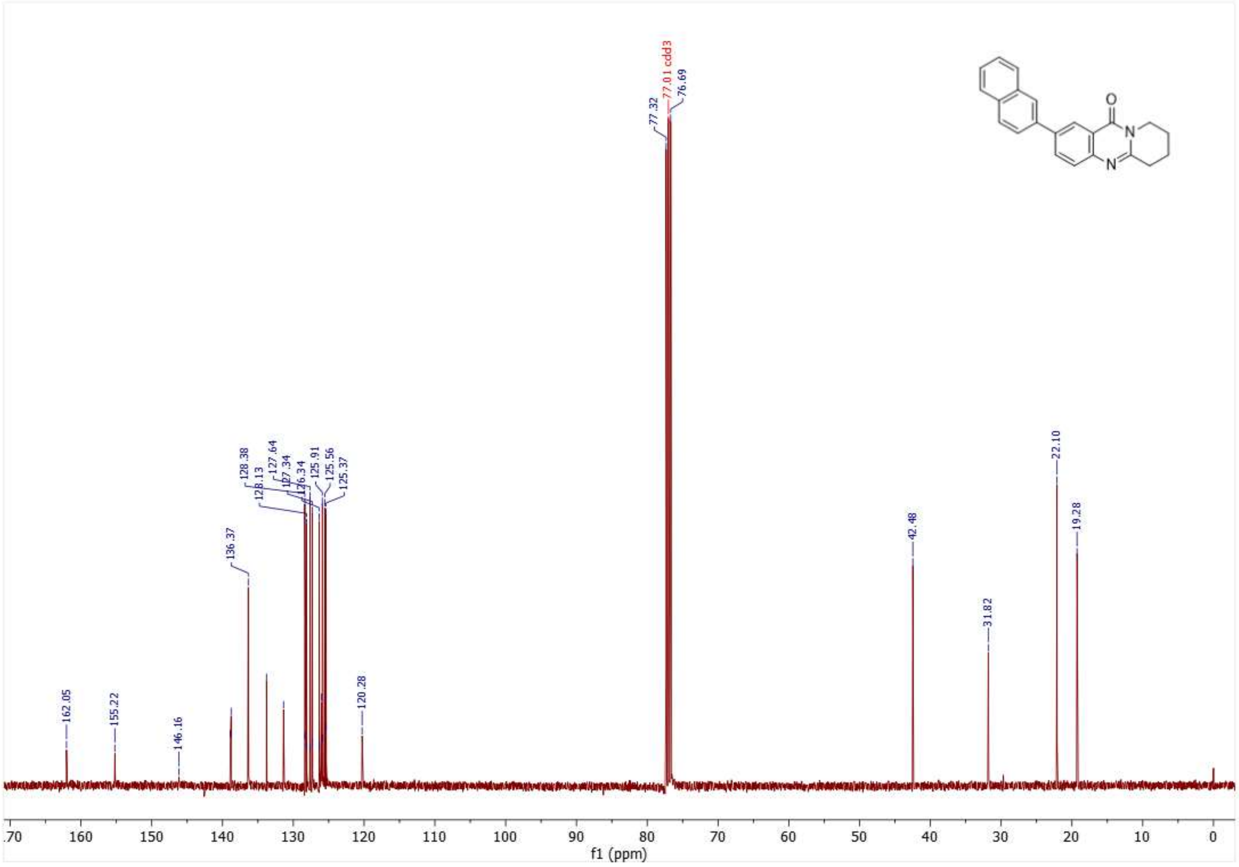

**Figure S80. ¹³C NMR spectrum of 4m**

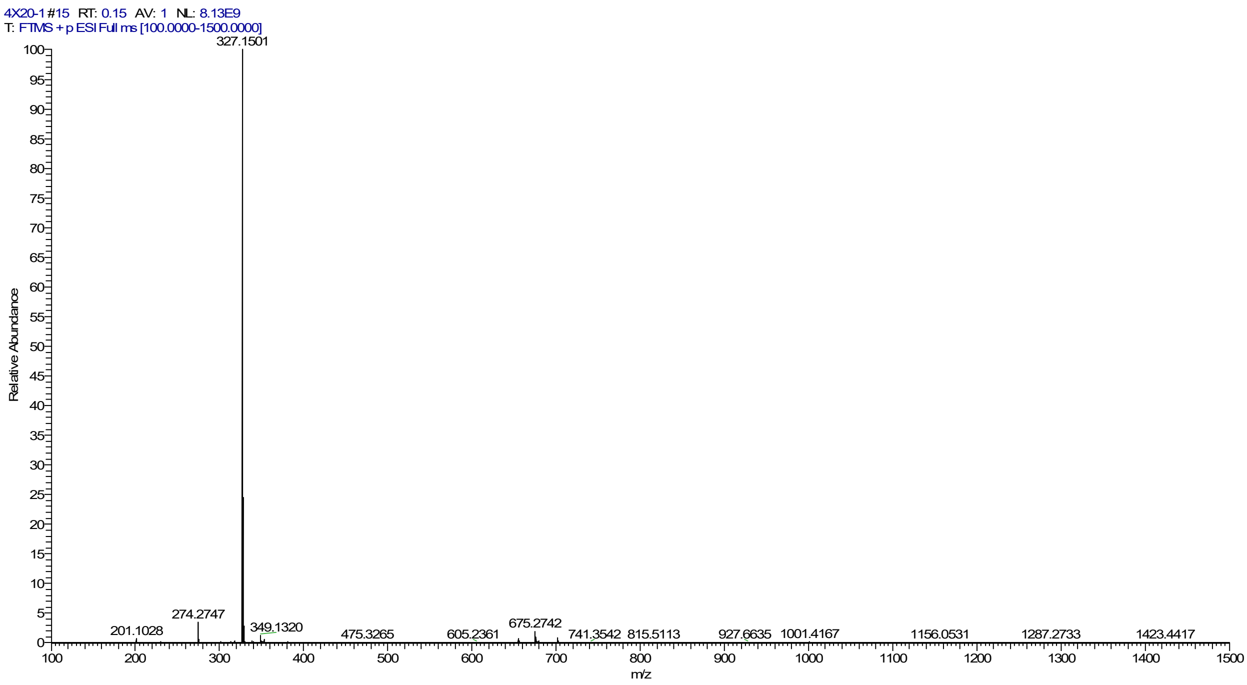


**Figure S81. Mass spectrum of 4m**

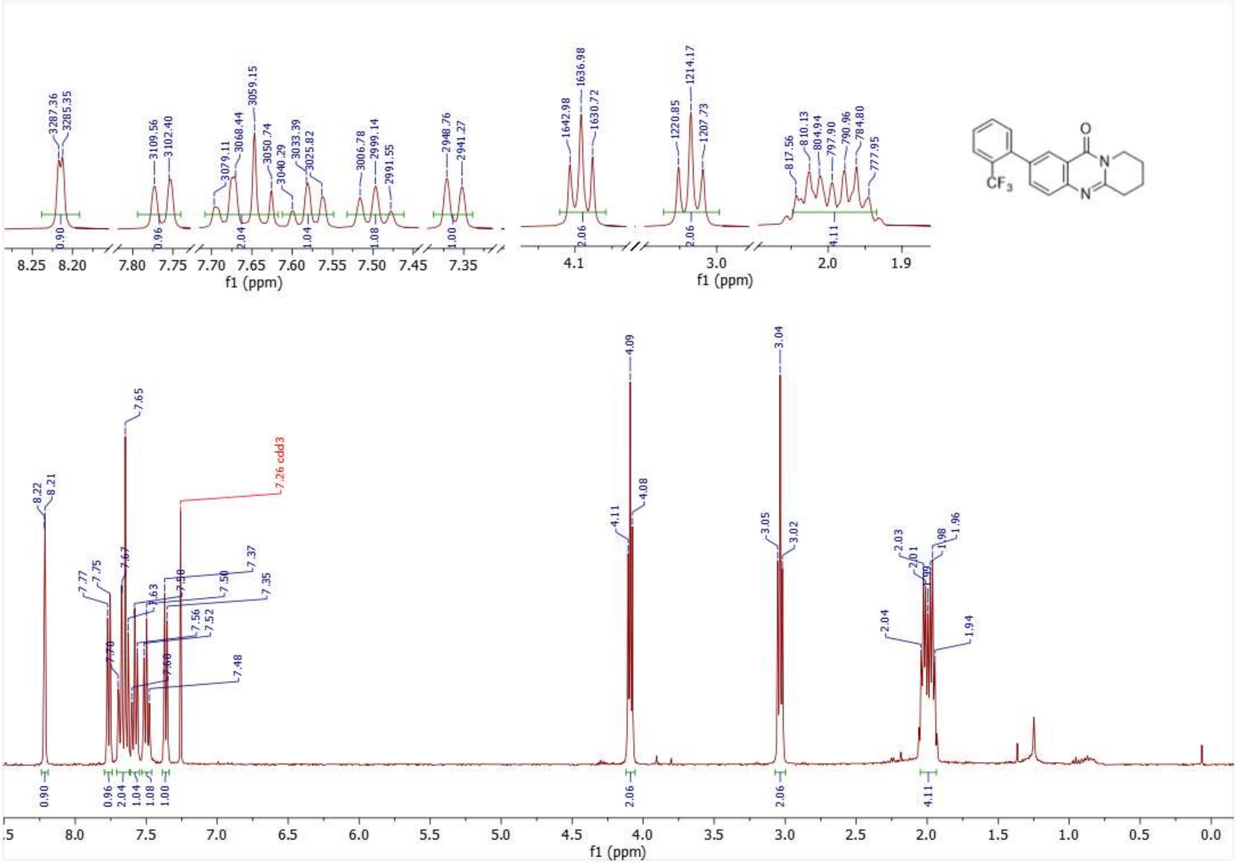


**Figure S82. $^{1}$H NMR spectrum of 4n**

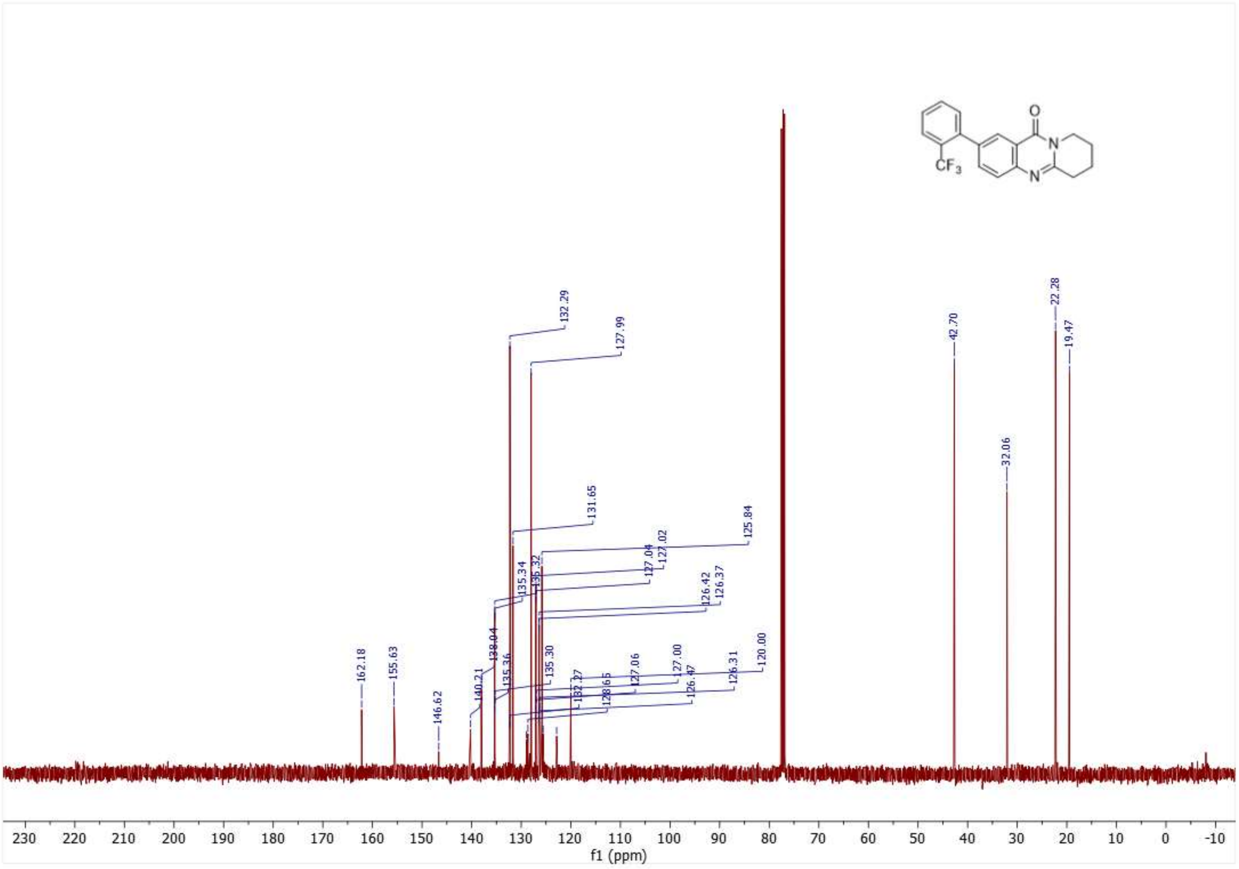

**Figure S83. $^{13}$C NMR spectrum of 4n**

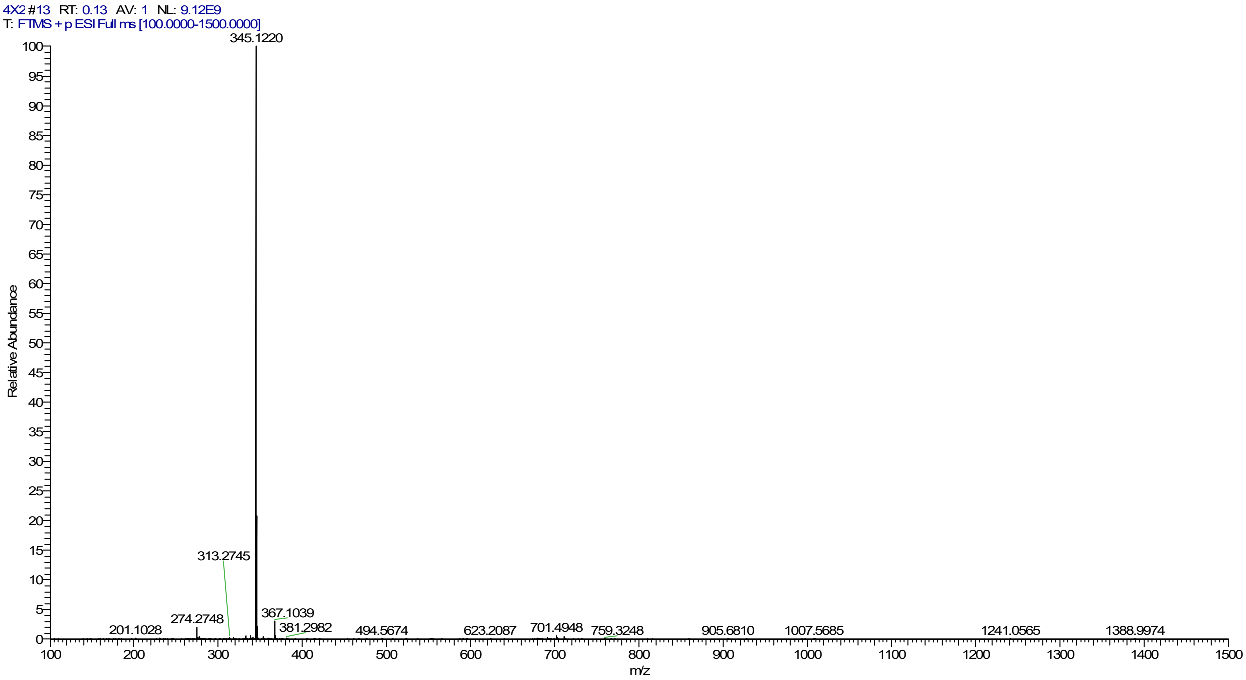


**Figure S84. Mass spectrum of 4n**
